# Automatic MS/MS Data Mining Strategy for Rapid Screening of Polyether Toxins Derived from *Gambierdiscus* Species

**DOI:** 10.1021/acs.analchem.4c06440

**Published:** 2025-03-04

**Authors:** Xiaowan Liu, Chenchen Xu, Jiajun Wu, Yock Haw Foo, Jin Zhou, Bin Wu, Leo Lai Chan

**Affiliations:** † The State Key Laboratory of Marine Pollution, City University of Hong Kong, Kowloon 999077, Hong Kong SAR, China; ‡ Ocean College, Zhejiang University, Zhoushan 321000, China; § Shenzhen Key Laboratory for the Sustainable Use of Marine Biodiversity, Research Centre for the Oceans and Human Health, City University of Hong Kong Shenzhen Research Institute, Shenzhen 518057, China; ∥ College of Computer Science and Technology, Zhejiang University, Hangzhou 310000, China; ⊥ Asian School of Environment, Nanyang Technological University, Singapore 637616, Singapore; # Shenzhen International Graduate School, Tsinghua University, Shenzhen 518055, China

## Abstract

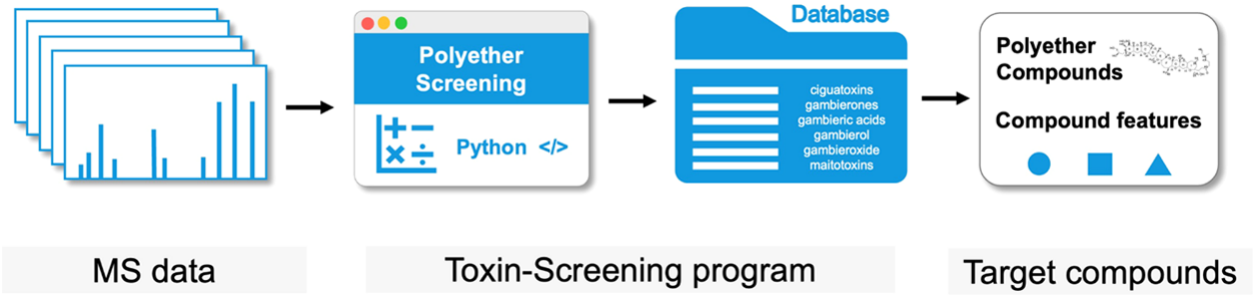

*Gambierdiscus* is a primary producer of diverse polyether toxins that can biomagnify and transform within marine food webs, posing major risks to marine organisms and human health. Currently, many toxins derived from *Gambierdiscus* remain unidentified. Existing toxin analysis methodologies primarily rely on known toxins, limiting the representation of toxin diversity and complexity and potentially underestimating associated risks. Herein, we present a Toxin-Screening program for high-throughput screening of polyether compounds by analyzing MS^2^ fragmentation patterns of detected ions and identifying Pacific Ocean ciguatoxins (P-CTXs) and gambierones through specific ion recognition. Using the Toxin-Screening program, eight P-CTXs purified from fish and *Gambierdiscus* spp., alongside two commercial gambierones standards, were successfully extracted from 5027 MS^2^ spectra and annotated. This method was subsequently applied to profile polyether compounds in three *Gambierdiscus caribaeus* strains, revealing only ten polyether compounds shared among the strains, while strain-specific compounds dominated. All *G. caribaeus* strains were found to produce gambierone, with levels notably varying among the strains. Several polyether compounds containing one or two SO_3_ groups suggest a potential novel toxin family that warrants further investigation.

## Introduction

Ciguatera food poisoning (CFP) is a nonbacterial foodborne illness originally prevalent in tropical and subtropical regions.^1^ Patients with CFP may experience gastrointestinal, cardiological, and neurological disorders lasting from weeks to years,^2^ with severe cases potentially leading to fatal outcomes.^2^ More than 50,000 cases of intoxication are estimated annually.^3^ Moreover, advancements in transportation, climate change, and eutrophication have led to a yearly increase in CFP incidents, posing major risks to public health and the global fishery economy.^4^ The causative agents of CFP are toxins originating from dinoflagellate genera, notably *Gambierdiscus* and *Fukuyoa*.^5^ Most of these toxins are lipophilic compounds that bioaccumulate in marine animal tissues and undergo oxidative modification, forming various analogs.^6,7^ Extensive chemical studies conducted on *Gambierdiscus* species and contaminated seafood have identified a total of 68 toxins, including 45 ciguatoxins (CTXs), nine gambierones, four gambieric acids, one gambierol, one gambieroxide, and eight maitotoxins (MTXs) (Table S1).^5,8−12^ The comprehensive detection and precise quantification of toxins in marine samples and seafood are crucial to effectively assess and mitigate CFP risks. Both biological methods (i.e., animal assays, cell-based assays, receptor-binding assays, or immunoassays) and chemical methods (i.e., nuclear magnetic resonance (NMR) or mass spectrometry (MS)) are used for toxin detection.^13^ Although biological methods enable rapid toxicity detection, they also offer limited toxin profiling. In addition, the trace levels of toxins are often below the NMR detection thresholds. Therefore, high-performance liquid chromatography coupled with tandem high-resolution mass spectrometry (HPLC–HRMS/MS) has become the preferred approach for detecting and characterizing these compounds.

The high–performance liquid chromatography–tandem mass spectrometry (HPLC–MS/MS) analytical method commonly employs multiple reaction monitoring (MRM) modes to quantify known toxins, offering excellent sensitivity and specificity.^14^ However, this approach heavily depends on the availability of reference standards, resulting in many toxins being overlooked and associated risks being underestimated.^15^ Recent years have seen remarkable progress in natural product discovery, HRMS data, and computational tools to promote compound discovery. Notable strategies include Global Natural Products Social Molecular Networking (GNPS), feature-based molecular networking, MS^2^ latent Dirichlet allocation, and SIRIUS.^16−19^ The molecular networking approach allows for the sorting of spectra based on specific criteria, highlighting those spectra that are likely to be of interest.^20,21^ GNPS, a spectral correlation and visualization approach, has successfully identified analogs of dinophysistoxins from *Prorocentrum lima* and pectenotoxins from *Dinophysis* species.^22,23^ Toxins derived from *Gambierdiscus* are a series of complex molecules (molecular weight >700 Da) comprising contiguous cyclic ether rings aligned in a ladder-like structure.^13^ Sequential dehydration events are a typical feature in MS^2^ fragmentation of polyether compounds and serve as an essential prerequisite for identifying *Gambierdiscus* toxins.^11^ However, these ladder-polyether toxins have limited shared fragment ions, and existing molecular networking approaches have not consistently clustered them together using reliable criteria, such as a cosine score of above 0.7 and more than six matched peaks.^24,25^ MS^2^ fragmentation patterns are widely recognized as pivotal in characterizing *Gambierdiscus* toxins. For instance, toxins, such as C-CTX-3, C-CTX-4, 12,13-dihydro-44-methylgambierone, sulfo-gambierone, and MTX-4, have been structurally characterized through MS^2^ fragmentation analysis.^11,26−28^ However, manually interpreting the massive data required for high-throughput toxin screening is time-consuming and challenging, underscoring the importance of developing complementary strategies leveraging computational tools and fragmentation features to assist the discovery and structural elucidation of these polyether toxins.

**Figure 1:**
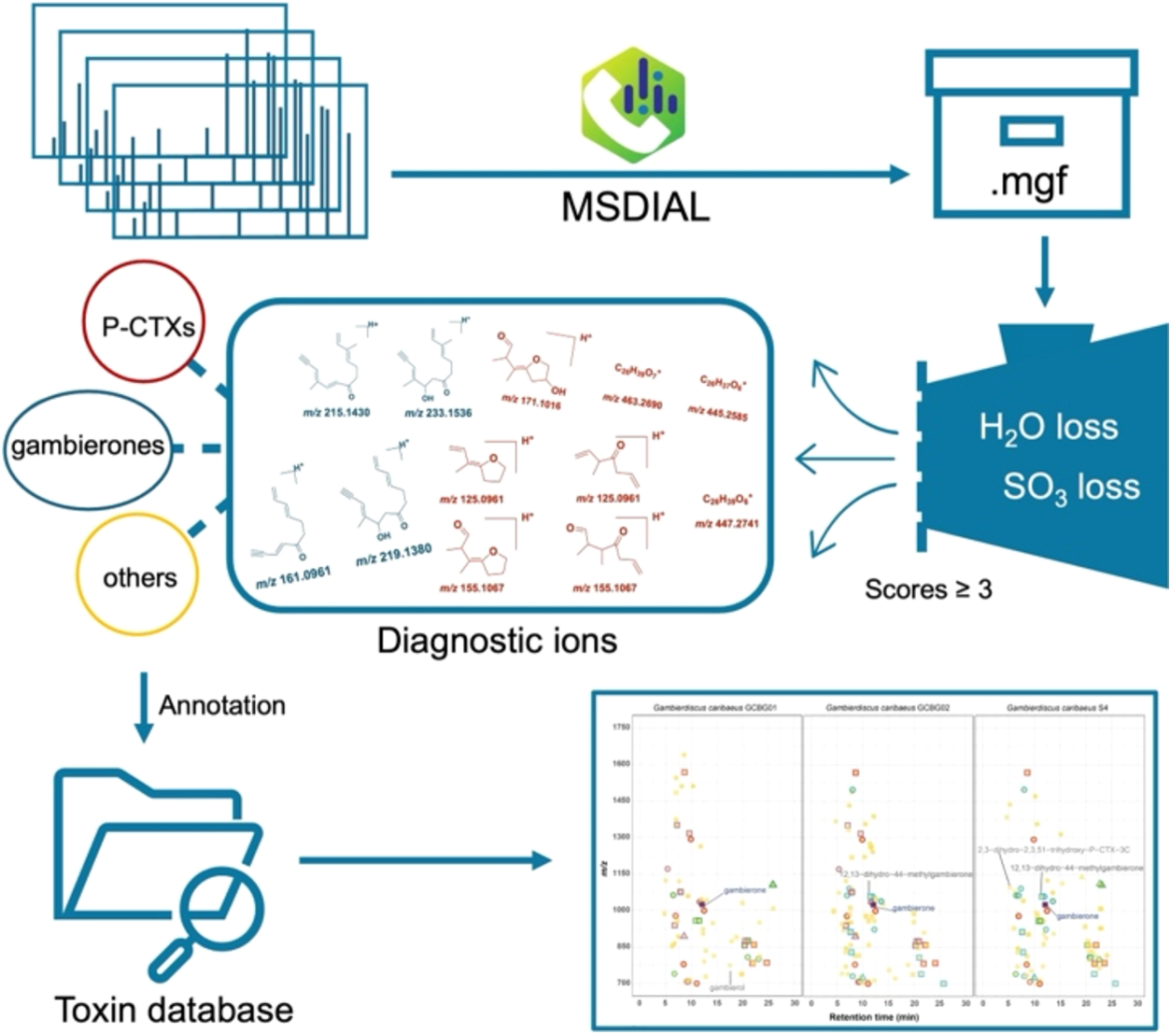
Schematic workflow of the Toxin-Screening program.

Herein, we propose a strategy named toxin screening (Figure 1) for the nuanced detection of polyether compounds, utilizing tailored algorithms that significantly enhance specificity and accuracy. Polyether compounds were extracted from extensive mass spectral data sets and subsequently annotated using a *Gambierdiscus* toxin data set comprising proton, ammonium, and sodium precursor adducts. Additionally, polyether compounds with specific ions of gambierones and P-CTXs can be classified into both toxin families. Although MS^2^ data alone cannot fully resolve structural details of stereochemical information, our approach focuses on identifying and linking atoms based on bond multiplicities. Our method can expedite the identification of known toxins in untargeted workflows, especially when reference materials are unavailable, and it aids in describing and predicting the molecular properties of new polyether compounds for further structural characterization.

## Results

### Fragmentation Patterns of P-CTXs and Gambierones

To investigate the chromatographic features and MS^2^ fragmentation patterns of P-CTXs and gambierones, eight P-CTXs (i.e., P-CTX-1, P-CTX-2, P-CTX-3, P-CTX-4A, P-CTX-3C, 2,3-dihydroxy-CTX-3C, 49-*epi*-CTX-3C, and M-*seco*-CTX-3C) purified from fish and *Gambierdiscus* spp., along with two commercial standards of gambierones (i.e., gambierone and 44-methylgambierone), were utilized for fragmentation analysis. Positive electrospray ionization mode (ESI^+^) was selected for untargeted HPLC-HRMS/MS analysis, as it provides more detailed information compared to the negative ionization mode.

**Figure 2:**
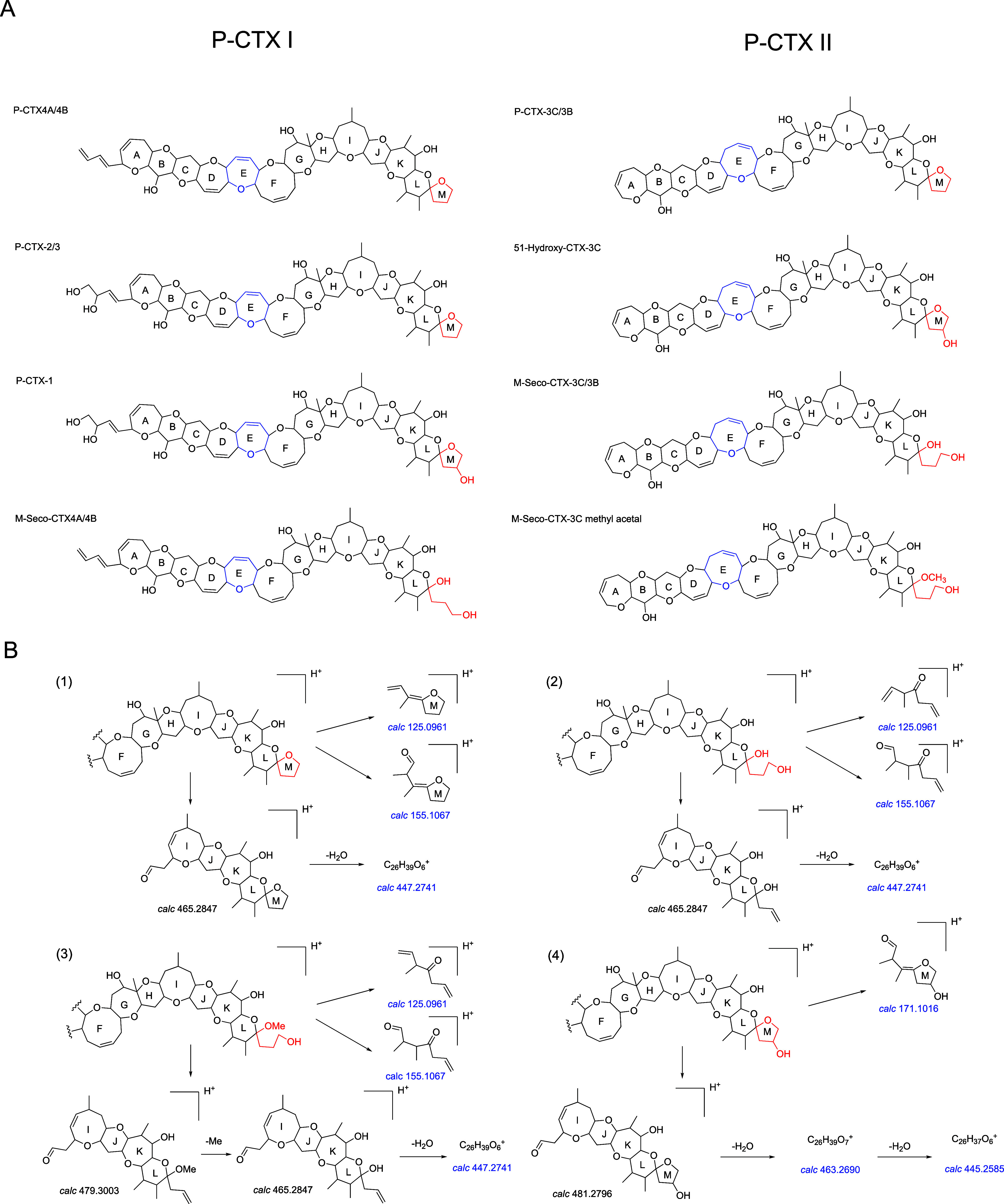
(A) Structures of two main groups of P-CTX congeners: P-CTX I and P-CTX II. (B) Proposed mechanism of the main fragmentation pathways for P-CTXs.

Among the various *Gambierdiscus*-associated toxins, CTXs are the most intensively investigated toxin family. The structure of CTXs varies according to geographic location, leading to classification as Pacific Ocean (P-CTX), Caribbean Sea (C-CTX), and Indian Ocean (I-CTX) ciguatoxins.^13^ P-CTXs are divided into two distinct subgroups: P-CTX I and P-CTX II. Both subgroups share a similar 13-ring structure but differ primarily in the size of the E-ring and the presence of a side chain at the A-ring.^10^ Specifically, P-CTX I has an aliphatic side chain on the A-ring and a seven-membered E-ring, whereas P-CTX II lacks these side chains and possesses an eight-membered E-ring (Figure 2A). Notably, both P-CTX types exhibit identical structural features from the F to M rings, resulting in the same ion fragments (i.e., mass-to-charge (*m*/*z*) = 125, 155, and 447) in their MS^2^ spectra (Figure S1). A thorough examination of the structure at the right-side terminal of all reported P-CTXs revealed four different side chains at the M-ring: the spiro-carbon M-ring (i.e., P-CTX-4A/4B, P-CTX-2/3, and P-CTX-3*C*/3B), M-*seco* hydroxyl terminal (i.e., M-*seco*-CTX-4*A*/4B and M-*seco*-CTX-3*C*/3B), M-*seco* methyl acetal terminal (i.e., M-*seco*-CTX-3C methyl acetal), and spiro-carbon M-ring with a hydroxy substituent (i.e., P-CTX-1 and 51-hydroxy-CTX-3C) (Figure 2B). For P-CTXs possessing the first three mentioned terminals, the cleavage of the L ring yields intense ions at *m*/*z* 125 and 155.^29^ Additionally, the cleavage of the H-ring, followed by the loss of H_2_O or methyl groups, generates the ion fragment at *m*/*z* 447 (Figures 2B and S1). For P-CTXs, such as P-CTX-1 and 51-hydroxy-CTX-3C, which have an acetal spiro-carbon M-ring with a hydroxy substituent, the cleavage of the L-ring forms the intense ion at *m*/*z* 171. The cleavage of the H-ring, accompanied by H_2_O losses, generates fragment ions at *m*/*z* 463 and 445 (Figures 2B and S1). Consequently, the fragment ions at *m*/*z* 125, 155, 447, 171, 445, and 463 can serve as distinctive markers for the detection of P-CTXs using nontarget HPLC–HRMS/MS methods.

**Figure 3:**
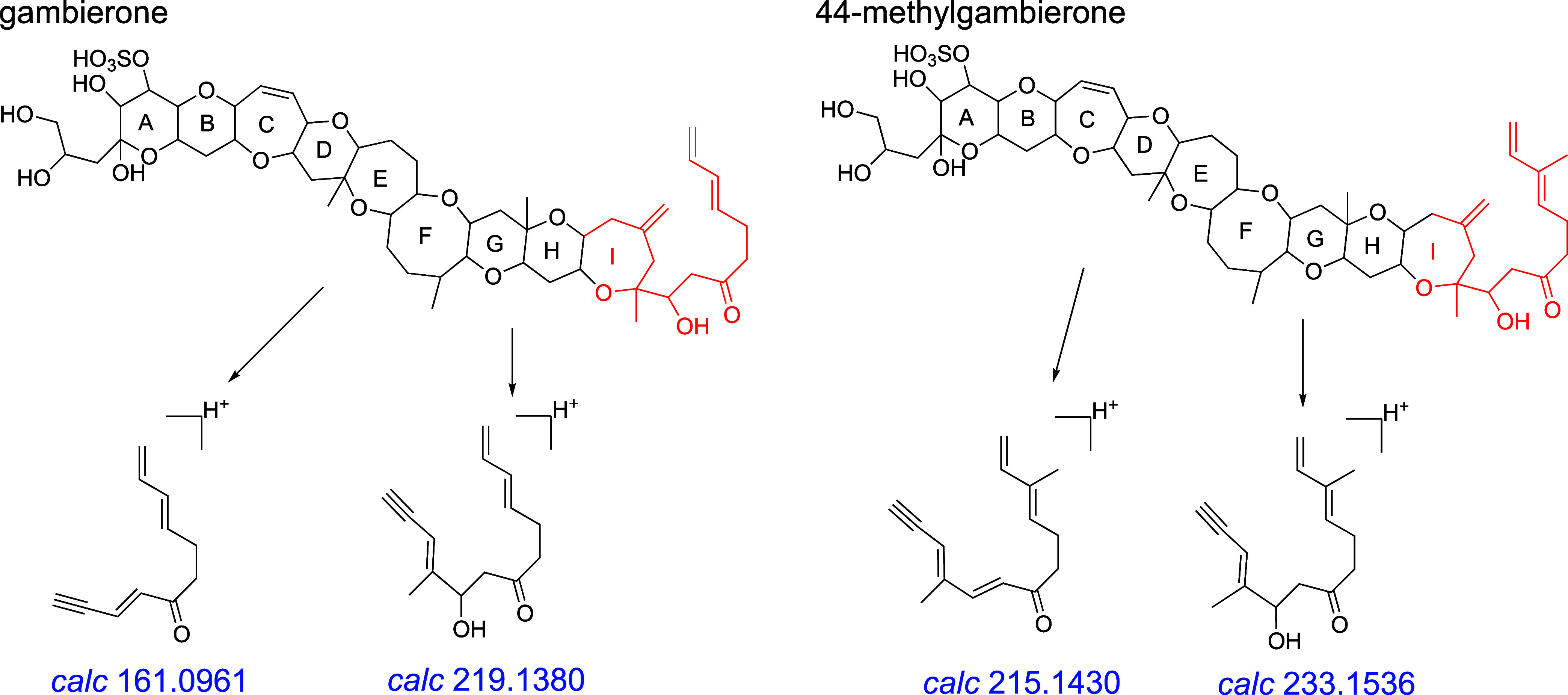
Proposed main fragmentation pathways for gambierone and 44-methylgambierone.

Gambierones, a class of sulfated ladder-polyether toxins, are widely distributed among diverse *Gambierdiscus* species, making them potential biomarkers for monitoring *Gambierdiscus* dominance in coral reef ecosystems.^30^ However, the exploration of gambierones remains in its preliminary stages, with only nine identified to date, six of which have been structurally characterized.^11,29,31^ Analysis of the MS^2^ spectra and fragmentation pathways of the standards revealed that the most intense peaks corresponded to the precursor ions and their series of polyenes derived from sequential dehydration and desulfation (Figure S1). Additionally, fragments (i.e., *m*/*z* 161, 219, 215, and 233) resulting from the cleavage of ring I were observed as prominent peaks (Figures 3 and S1). These typical features were also observed in other reported gambierones.^11,26^ Thus, in nontargeted HPLC–HRMS/MS analysis, fragment ions at *m*/*z* 161, 219, 215, and 233 could serve as potential target ions for the screening gambierones among polyether compounds.

In addition to characteristic ions, losses of H_2_O and SO_3_ were the major fragmentation behaviors observed in these polyether compounds, thereby providing a distinct profile in their MS^2^ spectra (Figure S1). During MS^1^ analysis, precursor ions for subsequent MS^2^ fragmentation typically involved three main types of adducts: protonated ([M + H] ^+^), ammonium ([M + NH_4_] ^+^), and sodium ([M + Na] ^+^) adducts. The relative intensity of these adducts for each polyether compound was influenced by the LC elution and MS interface conditions applied.^32,33^ Herein, ammonium adducts were most intense for P-CTX type I compounds (i.e., P-CTX-4A, P-CTX-1, P-CTX-2, and P-CTX-3), while protonated adducts predominated for P-CTX type II compounds (i.e., P-CTX-3C, 2,3-dihydroxy-3C, 49-*epi*-3C, and M-*seco*-3C) and gambierones (i.e., gambierone and 44-methylgambierone) (Figure S2). The effectiveness of MS/MS analyses was strongly associated with the intensity of the MS^1^ ions, as high-intensity ions were most likely to undergo subsequent MS^2^ fragmentation. To quantify H_2_O and SO_3_ losses in the screening of polyether compounds, the largest product/precursor ions of each compound within their MS^2^ spectra were identified and compared with those of other fragment ions. However, fragmentation ions were influenced by both precursor ion intensity and collision energy (CE) for sample analysis. Higher CE induced additional fragmentation ions but decreased the precursor ion intensity in MS^2^ spectra. While an appropriate CE was selected for sample analysis, it might not universally optimize every compound, potentially leading to the absence of precursor ions in some MS^2^ spectra. Therefore, based on the MS^2^ spectra of polyether compounds in previous studies,^11,25^ a variety of ion types was considered for screening of the largest product/precursor ions (Table 1). This finding, combined with the occurrence of H_2_O and SO_3_ losses, was integrated into a Python algorithm for screening polyether compounds.

**Table 1:** Ion Types of the Largest Product/Precursor Ions within the MS^2^ Spectra of the Detected Compounds

adduct type	ion types
protonated	[M + H]^+^, [M-H_2_O + H]^+^, [M-SO_3_ + H]^+^, [M-SO_3_–H_2_O + H]^+^
ammonium	[M + NH_4_]^+^, [M-H_2_O + NH_4_]^+^, [M-SO_3_ + NH_4_]^+^, [M-H_2_O–SO_3_ + NH_4_]^+^, [M + H]^+^, [M-H_2_O + H]^+^, [M-SO_3_+H]^+^, [M-SO_3_–H_2_O + H]^+^
sodium	[M + Na]^+^, [M-H_2_O + Na]^+^, [M-SO_3_ + Na]^+^, [M-H_2_O–SO_3_ + Na]^+^, [M + H]^+^, [M-H_2_O + H]^+^, [M-SO_3_ + H]^+^, [M-SO_3_–H_2_O + H]^+^

### Construction of Toxin-Screening Program

Based on the characteristic ions and fragmentation patterns of these polyether compounds described above, we developed the Toxin-Screening program, a tool designed to automate the high-throughput screening and annotation of targets from crude extracts. In this context, we established the following nomenclature: precursor ions were denoted as “*m*_p_,” the largest product/precursor ions within the MS^2^ spectra as “*m*_h_,” other peaks in the MS^2^ spectra as “*m*_i_,” neutral loss moieties as “*m*_n_,” and the precursor ions in the *Gambierdiscus* toxin database as “*m*.” Following the published compound confirmation criteria for time-of-flight (TOF)-HRMS, the allowable mass tolerance is limited to 0.01 Da and the mass error is maintained within 10 ppm, whichever was more accurate was applied.
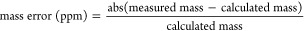


#### Step 1: Finding *m*_h_


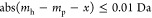


The first step entails determining the “*m*_h_” using the formula outlined above for subsequent calculations. A series of “*x*” values were applied sequentially within the formula based on the variation between the precursor and fragment ions (Table S2). If the program successfully identified an “*m*_h_” that fulfilled the formula, then the operation was terminated, and the process moved to the next step.

#### Step 2: Calculating H_2_O and SO_3_ Losses


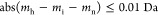


The second step involves calculating the occurrences of H_2_O and SO_3_ losses to screen for polyether compounds. A set of “*m*_n_” values (Table S3) was utilized in the formula, with each fragment ion that met the above criteria contributing one point to the score for the precursor ions. Ions with a cumulative score of three or more were selected for further analysis.

#### Step 3: Polyether Compound Classification

The third step utilizes diagnostic ions to precisely identify compounds such as P-CTXs and gambierones among the screened polyether compounds. For both toxin types, two criteria apply: a compound is classified into a specific toxin family if its MS^2^ fragments meet either criterion.

For P-CTXs, a compound is recognized if it contains either of the following sets of three diagnostic ions, with a mass error of less than 10 ppm: (1) *m*/*z* 125.0961 (C_8_H_13_O^+^), 155.1067 (C_9_H_15_O_2_^+^), 447.2741 (C_26_H_39_O_6_^+^), or (2) *m*/*z* 171.1016 (C_9_H_15_O_3_^+^), 445.2585 (C_26_H_37_O_6_^+^), and 463.2690 (C_26_H_39_O_7_^+^).

For gambierones, a compound is classified if it includes either of the following pairs of diagnostic ions, also with a mass error of less than 10 ppm: (1) *m*/*z* 161.0961 (C_11_H_13_O^+^) and 219.1380 (C_14_H_19_O_2_^+^), or (2) *m*/*z* 215.1430 (C_15_H_19_O^+^) and 233.1536 (C_15_H_21_O_2_^+^).

#### Step 4: Known Toxin Annotation

The final step involved toxin annotation using the *Gambierdiscus* toxin data set (Appendix I). Among the screened polyether compounds, precursor ions that matched ions in the data set with a mass error of less than 10 ppm were considered likely known toxins. Finally, precursor ions (*m*/*z* from 700 to 2000 Da) that exclusively accumulated in samples were screened.

### Validation of Toxin-Screening Program

**Figure 4:**
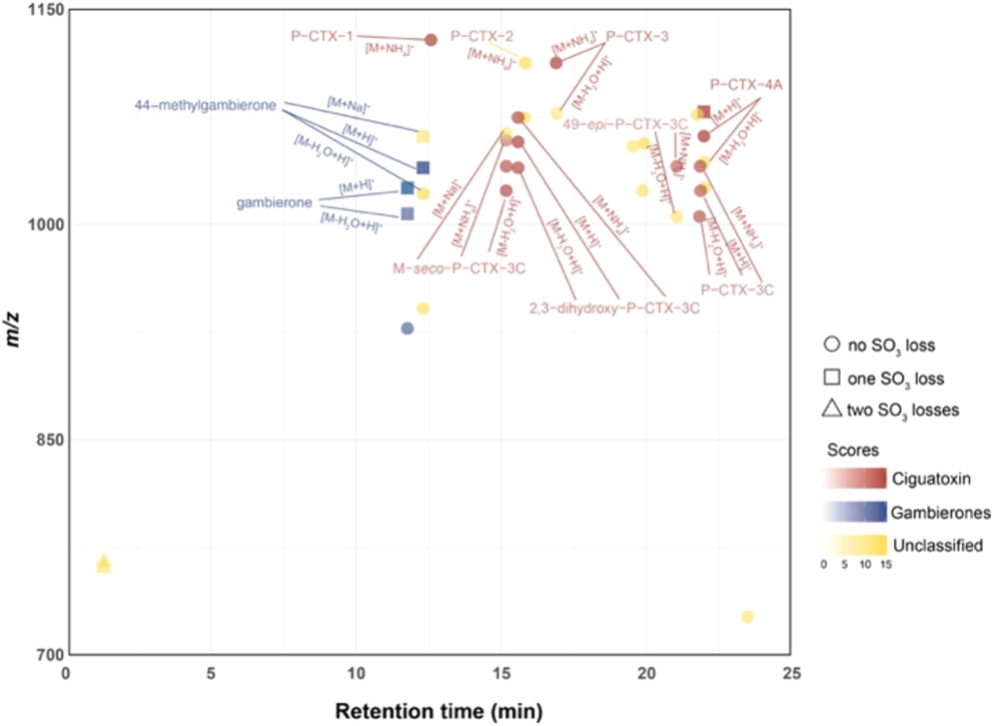
Toxin detection and annotation were carried out using the Toxin-Screening program.

Next, eight P-CTXs and two gambierones (Figure S3) were utilized to validate the Toxin-Screening program. Ten toxins were successfully extracted from 5027 MS^2^ spectra and annotated using the Toxin-Screening program (Figure 4 and Appendix II). However, several precursor ions, such as [M + Na]^+^ ions of 44-methylgambierone and M-*seco*-P-CTX-3C, [M + NH_4_]^+^ ion of P-CTX-2, and [M – H_2_O + H]^+^ ions of 44-methylgambierone, P-CTX-3, 49-*epi*-P-CTX-3C, and P-CTX-4A, could not be definitively classified into their respective toxin families owing to insufficient intensity of MS^2^ diagnostic ions for detection. Despite these limitations, these adducts can be recognized using their fragmentation patterns and annotated with the toxin database. Notably, the program enables the integration and visualization of toxin names, families, and atomic compositions (i.e., containing SO_3_ and NH_3_). Compared to the GNPS platform, our approach greatly enhances polyether compound discovery and simplifies the analytical process. The GNPS platform struggles to cluster these toxins effectively (Figure S4), requiring manual curation and expertise to distinguish them from extensive data sets. Importantly, the Toxin-Screening program cannot distinguish isomers, necessitating further analysis of additional properties, such as retention time, for precise toxin identification.

### Application of Toxin-Screening Program for Mapping the Polyether Fingerprint of *Gambierdiscus*

**Figure 5:**
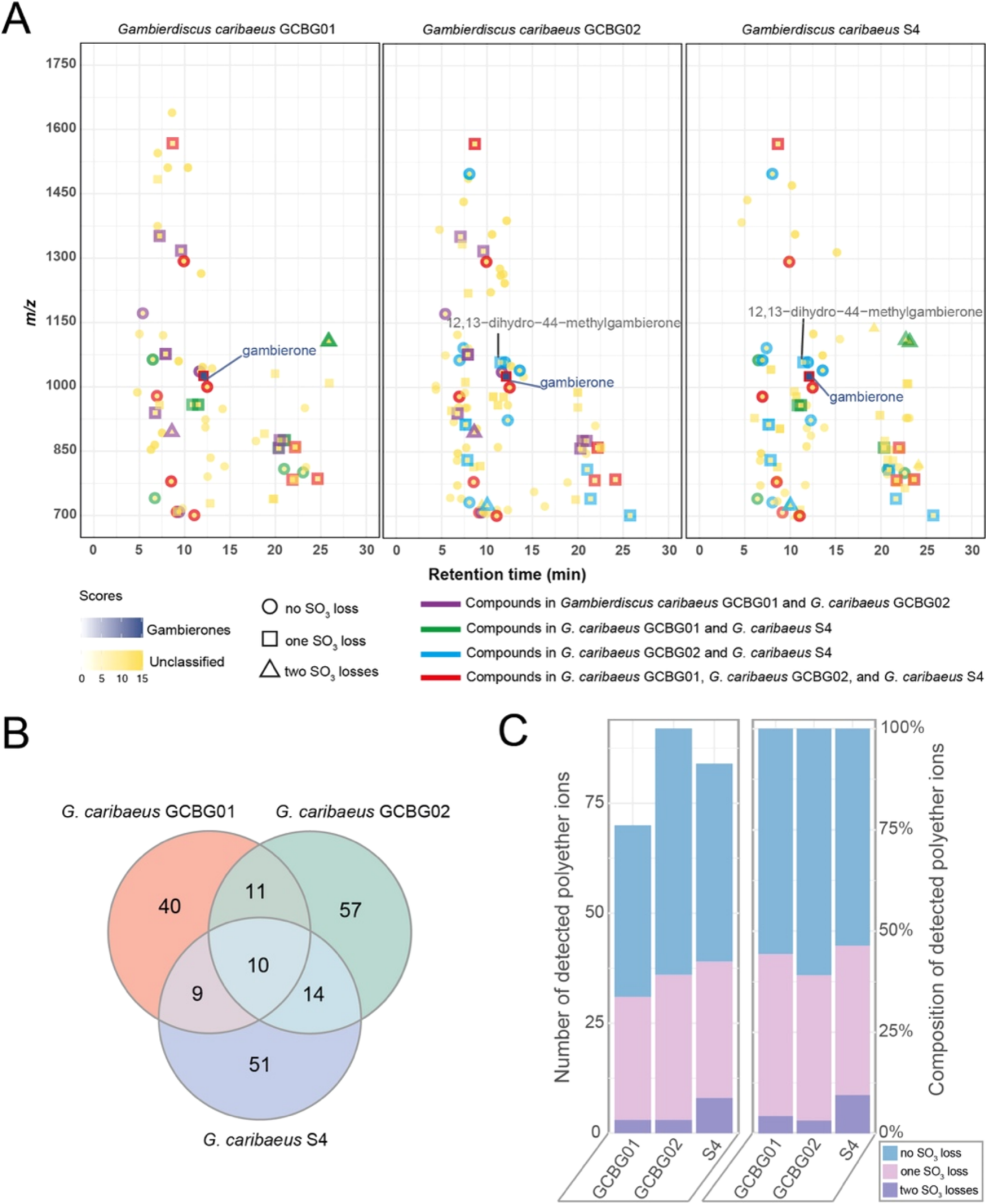
(A) Polyether compounds from three *G. caribaeus* strains. (B) Venn diagram showing the shared and specific polyether compounds across the three *G. caribaeus* strains. (C) Composition of polyether compounds in different *G. caribaeus* strains.

Polyether compounds from three *Gambierdiscus caribaeus* strains were investigated by using the Toxin-Screening program. To avoid duplicate analysis of compounds within a single sample, different adduct types were aligned based on identical retention times and MS^2^ fragments (Figures S5–S7 and Tables S4–S6). The results revealed that 70, 92, and 84 polyether compounds were screened in *G. caribaeus* GCBG01, *G. caribaeus* GCBG02, and *G. caribaeus* S4, respectively (Figure 5A–5C). These compounds exhibited similar polarity and *m*/*z* ratios across the three strains; for example, ions at *m*/*z* 1100–1700 Da eluted at 5–12.5 min, while ions at *m*/*z* 700–1100 eluted within both 5–12.5 and 20–25 min times windows (Figure 5A). Strain-specific compounds were predominant polyethers in *G. caribaeus* GCBG01, *G. caribaeus* GCBG02, and *G. caribaeus* S4 strains, representing 57, 62, and 61% of the total polyether compounds, respectively. Only ten shared compounds were detected across the three strains, with a mass tolerance of <0.01 Da and a retention time shift of <0.2 min (Figure 5B and Table S7). Additionally, the loss of SO_3_ was a distinct feature distinguishing gambierones and MTX analogs from other polyether compounds. For instance, gambieones typically contained one or two sulfate moieties, while MTXs always had two sulfate ester groups.^11,26,34^ Polyethers with one SO_3_ loss made up the second highest proportion in all three strains (*G. caribaeus* GCBG01, 40%; *G. caribaeus* GCBG02, 36%; *G. caribaeus* S4, 37%), whereas polyethers with two SO_3_ losses accounted for only 4, 3, and 10%, respectively (Figure 5C). Four polyether compounds possessing the SO_3_ group were detected in all strains, with precursor ions at *m*/*z* 783, 859, 1567, and 1025 (Table S7).

Putative gambierone was detected and annotated in three *G. caribaeus* strains. Five types of precursor adducts with scores above 4 were screened, including [M + NH_4_]^+^, [M + H]^+^, [M–H_2_O + H]^+^, [M–SO_3_–H_2_O + H]^+^, and [M–SO_3_–2H_2_O + H]^+^ (Figures S5–S7 and Tables S4–S6). Fragmentation features, such as sequential H_2_O losses, SO_3_ loss, and specific ions, were identified using the Toxin-Screening program. Additionally, their retention times, isotope distributions, and ion ratios were compared to those of the gambierone standard (Figure S8). Therefore, ions at *m*/*z* 1025 in all three *G. caribaeus* strains were proposed to be gambierone. Cellular gambierone production was evaluated across the three *G. caribaeus* strains. The highest production level of gambierone was observed in *G. caribaeus* GCBG01(119.38 ± 25.27 pg/cell), followed by *G. caribaeus* GCBG02 (72.48 ± 17.35 pg/cell) and *G. caribaeus* S4 (26.23 ± 2.51 pg/cell) (*p* < 0.05, One-way ANOVA with Duncan’s test) (Table S8). Ions at *m*/*z* 1041 and 1058 (retention time (RT) = 11.4 min) detected in *G. caribaeus* GCBG02 and *G. caribaeus* S4 were annotated as 12,13-dihydro-44-methylgambierone. However, they could not be classified into the gambierone family, as the diagnostic ions were not identified through the Toxin-Screening program (Figure 5A). To further characterize the “annotated 12,13-dihydro-44-methylgambierone,” we compared its RT, isotope distribution, precursor ions, MS^2^ fragment ions, and ion ratios with a previous study.^11^ Apparent differences in RT and ion ratios of precursors were observed (Figures S9 and S10), indicating that the ions at *m*/*z* 1041 and 1058 (RT = 11.4 min) were not the same precursor ions as 12,13-dihydro-44-methylgambierone. The full-scan mass spectra of *G. caribaeus* GCBG02 and *G. caribaeus* S4 showed three ions at *m*/*z* 1075, 1058, and 1041, which aligned to the same compound according to their matching RTs and MS^2^ fragments (Figures S10 and S11), suggesting that this compound is not an isomer of 12,13-dihydro-44-methylgambierone. The ion at *m*/*z* 1075 was the proposed ammonium adduct precursor ion ([M + NH_4_]^+^), while the ions at *m*/*z* 1058 and 1041 were likely protonated adducts of [M + H]^+^ and [M–NH_3_ + H]^+^, respectively (Figure S10). As shown in Figure 5A and Tables S5 and S6, the Toxin-Screening results suggested the presence of SO_3_ and NH_3_ groups in this compound, as confirmed via the MS^2^ data (Figure S11). Using the allowable elements and limits (C, 5–100; H, 0–200; O, 0–100; N, 0–; and S, 0–1) and a mass error <10 ppm, the empirical formula for this new compound was predicted (Table S9). This is the first known example of a polyether toxin containing both SO_3_ and NH_3_ groups. Further purification is required for NMR analysis to facilitate the structural elucidation of this compound.

## Discussion

The occurrence of CFP depends on the influx of CTXs from dinoflagellate genera, such as *Gambierdiscus*, into marine food webs. However, most causative CTXs have only been detected in fish, and the specific toxins potentially produced by *Gambierdiscus* species remain poorly understood.^6^ Bioassays and analytical chemistry approaches have demonstrated discrepancies in toxicity and polyether toxin profiles, both between species and among strains of the same species.^14^ Herein, we focused on *G. caribaeus*, a globally distributed species, to map polyether compound fingerprints. Substantial diversity in polyether compounds was observed across strains, even among those collected from the same geographical area and cultured under identical conditions. Only a limited number of polyether compounds (Figure 5B) were shared among these strains. Additionally, the production levels of gambierone, a shared polyether toxin, varied substantially among the three strains (Table S8). These findings indicate that the information provided by phylogenetic approaches may not accurately reflect the toxin production in *Gambierdiscus*. Additionally, unclassified polyether compounds with one SO_3_ loss comprised over one-third of the total polyethers, indicating new skeletons similar to those of gambierones that require further investigation. A review of all reported *G. caribaeus* strains indicated that most have the capacity to produce polyether toxins (Figure S12).^14,30,35−42^ However, assessing the geographic impact on toxin production within this species is challenging, as current studies primarily focus on a limited number of toxins, leaving many polyether toxins uninvestigated. Therefore, innovative tools such as the Toxin-Screening program developed in this study are essential for comprehensively profiling polyether compounds in *Gambierdiscus*, thereby enhancing our understanding of toxin production and transformation.

The Toxin-Screening program markedly simplifies polyether compound discovery, enabling high-throughput screening of targets in diverse samples. It successfully identified known gambierones and P-CTXs, classifying these two polyether families based on diagnostic fragments (Figures 4 and 5). Precursor ions with low abundance may generate low-quality MS^2^ spectra in information-dependent acquisition (IDA) mode, resulting in weak intensities of specific ions.^43^ Therefore, unclassified and unannotated polyether compounds remain targets for further analysis. This method enables the exploration of polyether toxin profiles in various samples beyond dinoflagellates. Additionally, the flexible code framework allows researchers to adjust diagnostic ions, extending high-throughput screening to other polyether compounds such as yessotoxin, brevetoxins, dinophysistoxin, okadaic acid, and azaspiracids. However, the limited range of standards analyzed may restrict the diagnostic ions to a subset of P-CTXs and gambierones. Additionally, the structural characterization of new polyether compounds relies heavily on mass data from known and well-characterized analogs, posing challenges for the further identification of screened targets. Future advancements may expand by integrating a broad array of substructures for fragment annotation.

## Summary

Herein, we propose an automatic MS/MS data mining strategy for the high-throughput screening of polyether compounds in the dinoflagellate genus *Gambiediscus*. Our approach facilitates the automatic extraction and visualization of polyether compound features, enabling the identification of potential new targets. Using this method, we conducted a comprehensive investigation of the polyether compound profile of three *G. caribaeus* strains, revealing a high abundance of polyether compounds within this species. Strain-specific polyethers were predominant, with polyethers exhibiting one SO_3_ loss accounting for over one-third of the total polyether compounds. Additionally, these strains demonstrated varying levels of gambierone production. To further improve this program, additional toxin standards and the acquisition of high-quality MS^2^ spectra are essential.

## Materials and Methods

### Algal Cultivation

*G. caribaeus* strains (GCBG01, GCBG02, and S4) were isolated from Weizhou island, Beibu Gulf, China. *Gambierdiscus balechii* 1123M1M10 was collected from Marakei Island, Republic of Kiribati, as reported in our previous study.^44^ They were cultured in a modified K medium prepared with artificial seawater at a salinity of 32 PSU and incubated at 22 °C ± 1 °C under a 12 h/12 h (light/dark) cycle with a light intensity of 70–90 mol photon m^–2^ s^–1^. For the study, 110 mL of algal cultures were prepared in three replicates for each strain under the above-mentioned conditions.

### Sample Preparation

Algal cells (2.8–6.4 × 10^4^ cells, Table S10) were collected via filtration using a 47 mm Isopore membrane (3 μm pore size; Millipore, Dublin, Ireland). The cell pellet was transferred to 15 mL centrifuge tubes (Corning, Gilbert, AZ) and resuspended in 8 mL of methanol (Merck, Darmstadt, Germany). Cell lysis was performed using an ultrasonic processor (Sonicator Q700, QSONICA, CT) at 30% amplitude for 2 min in pulse mode (5 s on, 5 s pause). Supernatants were collected after centrifugation at 12,000*g*, 4 °C for 10 min. The extraction process was repeated twice, and the combined extracts were dried under a gentle stream of high-purity nitrogen at 40 °C. The dried extracts were then subjected to C18 solid-phase extraction (SPE) cartridges. The SPE cartridges were preconditioned with sequential solvent addition: 10 mL of 100% methanol, 10 mL of 80% methanol, 10 mL of 40% methanol, and 10 mL of 20% methanol. Samples were redissolved in 2 mL of 20% methanol and loaded onto preconditioned cartridges. Then, the cartridges were eluted with four fractions: 10 mL of 20% methanol, 10 mL of 40% methanol, 10 mL of 80% methanol, and 10 mL of 100% methanol. All fractions were dried under a gentle stream of high-purity nitrogen and redissolved in 200 μL of methanol for HPLC–MS/MS analysis.

### Toxin Standards

P-CTX-1 was purified from moray eels as previously published.^19^ P-CTX-2 and P-CTX-3 were obtained from Prof. Richard Lewis (University of Queensland, Australia).^45^ P-CTX-3C, 2, 3-dihydroxy-CTX-3C, 49-*epi*-CTX-3C, M-*seco*-3C, and P-CTX-4A were provided by Dr Mireille Chinain from the Institut Louis Malardé’s bank of standards (ILM, French Polynesia). Gambierone and 44-methylgambierone were purchased from Laboratorio CIFGA S.A. (Lugo, Spain).

### Liquid Chromatography–Tandem Mass Spectrometry (LC–MS/MS) Analysis

The UPLC-Q-TOF system consisted of an Agilent 1290 Infinity LC system (Agilent, Palo Alto, CA) coupled with a Sciex X500R QTOF mass spectrometer system (AB Sciex, Foster City, CA), equipped with an ESI source operating in the IDA mode. Chromatographic separation was performed on a Phenomenex Kinetex C18 column (2.1 × 100 mm^2^, 1.7 μm). Gradient elution at a flow rate of 0.2 mL/min was performed using (A) Mill-Q water containing 0.02% formic acid (Merck, Darmstadt, Germany) and 2 mM ammonium acetate (Sigma-Aldrich, MO) and (B) 95% acetonitrile containing 0.02% formic acid and 2 mM ammonium acetate. The gradient elution started at 10% B, increased to 100% B over 20 min, was held for 4 min, and then returned to 10% B for 1 min. The column was equilibrated at initial gradient conditions for 5 min before each injection, with an injection volume of 5 μL. For the MS/MS experiment, IDA acquisition in positive ionization mode for nontarget analysis followed our previous study.^10^ The scan included a TOF-MS full-scan analysis (0.25 s) and up to 10 dependent MS/MS analyses (0.1 s for each MS/MS analysis) per cycle in the *m*/*z* range of 100–2000 for MS^1^ and 50–2000 for MS^2^. Source settings for the IDA mode were as follows: CE 35 V with collision energy spread (CES) 15 V; nebulizer gas (gas 1) at 30 psi; heater gas (gas 2) at 40 psi; curtain gas at 25 psi; ion source temperature at 500 °C; ion spray voltage floating at 5500 V; delustering potential at 80 V; and full MS collision energy at 10 V.

The instrumental method for the target analysis of gambierone followed our previous report.^10^ Their separation and quantification were performed using an Agilent 1290 Infinity ultrahigh pressure liquid chromatography (Agilent, Palo Alto, CA) interfaced with a 5500 QTRAP mass spectrometer (AB Sciex, Foster City, CA). Gambierone was detected using MRM in negative ESI mode to ensure optimal sensitivity and selectivity. The calibration curve (gambierone: *y* = 16,577*x* + 13893, *R*^2^ = 0.9986) was generated using a standard solution with six concentrations ranging from 0 to 100 ng/mL. The limits of detection (LOD) and quantification (LOQ) of analytical methods were determined based on signal-to-noise (S/N) ratios of 3:1 and 10:1, respectively. The LOD for gambierone was 0.09 ng/mL, and the LOQ was 0.3 ng/mL.

### Data Processing and Toxin-Screening Application

Mass data were processed using MSDIAL 4.9 to extract features and generate .mgf files. The detailed parameters for the MSDIAL 4.9 setting are listed in Table S11. Toxin-Screening, coded in Python, incorporates a *Gambierdiscus* toxin database (Appendix I), sequential dehydration and desulfonation features, and diagnostic ions of toxins. The .mgf files were input into Toxin-Screening for automatic toxic screening and annotation. The Toxin-Screening command and the user manual are available in the Supporting Information section. Results were further visualized using R version 4.2.

## Supporting Information Available

*Gambierdiscus* toxin data set, along with the original data from the validation and application processes of Toxin-Screening program (ZIP)
MS^1^ and MS^2^ data for the screened polyether compounds; additionally, MSDIAL parameters for data set processing, and user manual and code of the Toxin-Screening program (PDF)
