# Supplementary material for: Automatic MS/MS Data Mining Strategy for Rapid Screening of Polyether Toxins Derived from Gambierdiscus Species
Source: Anal Chem. 2025 Mar 4;97(10):5643–52. doi: 10.1021/acs.analchem.4c06440 (PMC11923951; doi:10.1021/acs.analchem.4c06440)
Supplement: Supplementary file 2 — ac4c06440_si_002.pdf [file ac4c06440_si_002.pdf]

SUPPORTING INFORMATION

An automatic MS/MS data mining strategy for rapid screening of polyether toxins derived from *Gambierdiscus* species

Xiaowan Liu [1], Chenchen Xu [4], Jiajun Wu [1,3], Yock Haw Foo [5], Jin Zhou [6], Bin Wu [2,] *, Leo Lai Chan [1,3,] *

[1] The State Key Laboratory of Marine Pollution, City University of Hong Kong, Hong Kong SAR 999077, China

[2] Ocean College, Zhejiang University, Zhoushan 321000, China

[3] Shenzhen Key Laboratory for the Sustainable Use of Marine Biodiversity, Research Centre for the Oceans and Human Health, City University of Hong Kong Shenzhen Research Institute, Shenzhen 518057, China

[4] College of Computer Science and Technology, Zhejiang University, Hangzhou 310000, China

[5] Asian School of Environment, Nanyang Technological University, Singapore 637616, Singapore

[6] Shenzhen International Graduate School, Tsinghua University, Shenzhen 518055, PR China

* (Email: wubin@zju.edu.cn; leochan@cityu.edu.hk)

Table of Contents

Table S1. Toxin derived from *Gambierdiscus* species. 3

Table S2. The differences between the precursor and fragment ions 4

Table S3. The information on neutral loss moieties....5

Table S4. The information of aligned adducts in *Gambierdiscus caribaeus* GCBG01 5

Table S5. The information of aligned adducts in *Gambierdiscus caribaeus* GCBG02 6

Table S6. The information of aligned adducts in *Gambierdiscus caribaeus* S4....6

Table S7. The information of ten shared polyether compounds across three *G.caribaeus* strains. 7

Table S8. The cellular level of gambierone in three *Gambierdiscus* strains....7

Table S9 The empirical formula of ion at *m/z* 1058 (11.4 min) prediction....7

Table S10. MSDIAL 4.9 parameters for LC-HRMS/MS datasets processing....8

Figure S1. Fragment ion spectra (MS^2, MS/MS) of $[M + NH_4]^+$ ions of P-CTX-4A (P-CTX I type), P-CTX-2 (P-CTX I type), P-CTX-3 (P-CTX I type), P-CTX-1 (P-CTX I type), and M-*seco*-CTX3C (P-CTX II type), $[M + H]^+$ ions of P-CTX-3C (P-CTX II type), 49-*epi*-3C (P-CTX II type), 2,3-dihydroxy-3C (P-CTX II type), gambierone, and 44-methylgambierone. Diagnostic ions were highlighted in cyan....10

Figure S2. Full-scan mass spectra (MS^1) of P-CTX-4A, P-CTX-1, P-CTX-2, P-CTX-3, P-CTX-3C, 2,3-dihydroxy-3C, 49-*epi*-3C, M-*seco*-3C, gambierone, and 44-methylgambierone. The ions with the highest intensity were shown in red colour.....11

Figure S3. Extraction ion chromatograms (XICs) of gambierone (100 ng/mL), 44-methylgambierone (100 ng/mL), P-CTX-1 (20 ng/mL), M-*seco*-3C (100 ng/mL), 2,3-dihydroxy-3C (100 ng/mL), P-CTX-2 (80 ng/mL), P-CTX-3 (80 ng/mL), 49-*epi*-3C (100 ng/mL), P-CTX-3C (100 ng/mL), and P-CTX-4A (100 ng/mL). 11

Figure S4. Molecular network (MN) of eight P-CTXs (i.e., P-CTX-1, P-CTX-2, P-CTX-3, P-CTX-4A, P-CTX-3C, 2,3-dihydroxy-CTX-3C, 49-*epi*-CTX-3C, and M-*seco*-CTX-3C) and two gambierones (i.e., gambierone, 44-methylgambierone). MN was created with the following parameters: cosine score of 0.7, minimum of 6 common fragment ions and a TopK set at 1000. Each node contained the *m/z* of the precursor ion and P-CTXs and gambierones were highlighted by pink and purple, respectively. 12

Figure S5. The MS^1 and MS^2 spectra of aligned adducts in *Gambierdiscus caribaeus* GCBG01 19

Figure S6. The MS^1 and MS^2 spectra of aligned adducts in *Gambierdiscus caribaeus* GCBG02 31

Figure S7. The MS^1 and MS^2 spectra of aligned adducts in *Gambierdiscus caribaeus* S4. 37

Figure S8. Extraction ion chromatograms (XICs) and MS^1 spectra of gambierone standard and putative gambierone from *G.caribaeus* GCBG01, *G.caribaeus* GCBG02, and *G.caribaeus* S4. 39

Figure S9. Extraction ion chromatograms (XICs) of ions at *m/z* 1041 from *G.balechii* 1123M1M10, *G.caribaeus* S4, and *G.caribaeus* GCBG02. 39

Figure S10. MS^1 spectra of 12,13-dihydro-44-methylgambierone from *G.balechii* 1123M1M10, and annotated 12,13-dihydro-44-methylgambierone from *G.caribaeus* S4 and *G.caribaeus* GCBG02. 40

Figure S11. MS^2 spectra of ions at *m/z* 1075, 1058, and 1041 from *G.caribaeus* GCBG02. 40

Figure S12. Distribution, toxicity assessment, and detection of polyether toxins in *Gambierdiscus caribaeus*. Species reported in the references are marked in red, while those described in this study are highlighted in yellow. 41

User manual and code of the Toxin-Screening program 41

Table S1. Toxin derived from *Gambierdiscus* species.

Type	Compound	Molecular formula	[M+H] +	[M+NH4] +	[M+Na] +
CTX1B (15)	P-CTX-1	$C_{60}H_{86}O_{19}$	1111.5836	1128.6101	1133.5655
	52-*epi*-P-CTX-1	$C_{60}H_{86}O_{19}$	1111.5836	1128.6101	1133.5655
	54-*epi*-P-CTX-1	$C_{60}H_{86}O_{19}$	1111.5836	1128.6101	1133.5655
	54-*epi*-52-*epi*-P-CTX-1	$C_{60}H_{86}O_{19}$	1111.5836	1128.6101	1133.5655
	54-deoxy-50-hydroxy-P-CTX-1	$C_{60}H_{86}O_{19}$	1111.5836	1128.6101	1133.5655
	P-CTX-2	$C_{60}H_{86}O_{18}$	1095.5887	1112.6152	1117.5706
	P-CTX-3	$C_{60}H_{86}O_{18}$	1095.5887	1112.6152	1117.5706
	3,4-dihydro-3-hydroxy-7-*oxo*-P-CTX-1	$C_{60}H_{88}O_{21}$	1145.5891	1162.6156	1167.571
	3,4-dihydro-4-hydroxy-7-*oxo*-P-CTX-1	$C_{60}H_{88}O_{21}$	1145.5891	1162.6156	1167.571
	7-*oxo*-P-CTX-1	$C_{60}H_{86}O_{20}$	1127.5786	1144.6051	1149.5605
	7-hydroxy-P-CTX-1	$C_{60}H_{88}O_{20}$	1129.5942	1146.6207	1151.5761
	CTX-4B	$C_{60}H_{84}O_{16}$	1061.5832	1078.6097	1083.5651
	52-*epi*-P-CTX-4B	$C_{60}H_{84}O_{16}$	1061.5832	1078.6097	1083.5651
	M-*seco*-CTX-4A	$C_{60}H_{86}O_{17}$	1079.5938	1096.6203	1101.5757
	M-*seco*-CTX-4B	$C_{60}H_{86}O_{17}$	1079.5938	1096.6203	1101.5757
CTX3C (16)	P-CTX-3C	$C_{57}H_{82}O_{16}$	1023.5676	1040.5941	1045.5495
	49-*epi*-P-CTX-3C	$C_{57}H_{82}O_{16}$	1023.5676	1040.5941	1045.5495
	51-hydroxy-P-CTX-3C	$C_{57}H_{82}O_{17}$	1039.5625	1056.5890	1061.5444
	2,3-dihydroxy-P-CTX-3C	$C_{57}H_{84}O_{18}$	1057.5731	1074.5996	1079.555
	A-*seco*-2,3-dihydro-51-hydroxy-P-CTX-3C	$C_{57}H_{86}O_{18}$	1059.5887	1076.6152	1081.5706
	2,3-dihydro-2,3,51-trihydroxy-P-CTX-3C	$C_{57}H_{84}O_{19}$	1073.5680	1090.5945	1095.5499
	M-*seco*-2,3-dihydro-2-hydroxy-49-*O*-methyl-P-CTX-3C	$C_{58}H_{88}O_{18}$	1073.6044	1090.6309	1095.5863
	2,3-dihydro-2-hydroxy-P-CTX-3C	$C_{57}H_{84}O_{17}$	1041.5782	1058.6047	1063.5601
	2,3-dihydro-3-hydroxy-P-CTX-3C	$C_{57}H_{84}O_{17}$	1041.5782	1058.6047	1063.5601
	M-*seco*-P-CTX-3C	$C_{57}H_{84}O_{17}$	1041.5782	1058.6047	1063.5601
	M-*seco*-P-CTX-3B	$C_{57}H_{84}O_{17}$	1041.5782	1058.6047	1063.5601
	2,3-dihydro-51-hydroxy-2-*oxo*-P-CTX-3C	$C_{57}H_{82}O_{18}$	1055.5574	1072.5839	1077.5393
	51-hydroxy-2-*oxo*-P-CTX-3C	$C_{57}H_{82}O_{18}$	1055.5574	1072.5839	1077.5393
	51-hydroxy-3-*oxo*-P-CTX-3C	$C_{57}H_{82}O_{18}$	1055.5574	1072.5839	1077.5393
	M-*seco*-P-CTX-3C methyl acetal	$C_{58}H_{86}O_{17}$	1055.5938	1072.6203	1077.5757
	M-*seco*-40-O-methyl-P-CTX-3C	$C_{58}H_{86}O_{17}$	1055.5938	1072.6203	1077.5757
C-CTXs (8)	C-CTX-1	$C_{62}H_{92}O_{19}$	1141.6306	1158.6571	1163.6125
	C-CTX-2	$C_{62}H_{92}O_{19}$	1141.6306	1158.6571	1163.6125
	C-CTX-3	$C_{62}H_{94}O_{19}$	1143.6462	1160.6727	1165.6281
	C-CTX-4	$C_{62}H_{94}O_{19}$	1143.6462	1160.6727	1165.6281
	C-CTX-5	$C_{62}H_{90}O_{19}$	1139.6149	1156.6414	1161.5968
	50,51-didehydro-C-CTX-3	$C_{62}H_{92}O_{19}$	1141.6306	1158.6571	1163.6125

	17-hydroxy-C-CTX-1	$C_{62}H_{92}O_{20}$	1157.6255	1174.6520	1179.6074
	17-hydroxy-50,51-didehydro-C-CTX-3	$C_{62}H_{92}O_{20}$	1157.6255	1174.6520	1179.6074
I-CTXs (6)	I-CTX-1	$C_{62}H_{92}O_{19}$	1141.6306	1158.6571	1163.6125
	I-CTX-2	$C_{62}H_{92}O_{19}$	1141.6306	1158.6571	1163.6125
	I-CTX-3	$C_{62}H_{92}O_{20}$	1157.6255	1174.6520	1179.6074
	I-CTX-4	$C_{62}H_{92}O_{20}$	1157.6255	1174.6520	1179.6074
	I-CTX-5	$C_{62}H_{90}O_{19}$	1139.6149	1156.6414	1161.5968
	I-CTX-6	$C_{62}H_{90}O_{20}$	1155.6098	1172.6363	1177.5917
Gambieric acids (4)	gambieric acid A	$C_{59}H_{92}O_{16}$	1057.6458	1074.6723	1079.6277
	gambieric acid B	$C_{60}H_{94}O_{16}$	1071.6615	1088.6880	1093.6434
	gambieric acid C	$C_{65}H_{100}O_{19}$	1185.6932	1202.7197	1207.6751
	gambieric acid D	$C_{66}H_{102}O_{19}$	1199.7088	1216.7353	1221.6907
Gambieroxide (1)	gambieroxide	$C_{60}H_{90}O_{22}S$	1195.5718	1212.5983	1217.5537
Gambierol (1)	gambierol	$C_{43}H_{64}O_{11}$	757.4522	774.4787	779.4341
Gambierones (9)	gambierone	$C_{51}H_{76}O_{19}S$	1025.4775	1042.5040	1047.4594
	44-methylgambierone	$C_{52}H_{78}O_{19}S$	1039.4931	1056.5196	1061.4750
	29-methylgambierone	$C_{52}H_{78}O_{19}S$	1039.4931	1056.5196	1061.4750
	sulfo-gambierone	$C_{51}H_{76}O_{22}S_2$	1105.4343	1122.4608	1127.4162
	dihydro-sulfo-gambierone	$C_{51}H_{78}O_{22}S_2$	1107.4499	1124.4764	1129.4318
	desulfo-hydroxyl-gambierone	$C_{51}H_{78}O_{17}$	963.5312	980.5577	985.5131
	38-dehydroxy-44-methylgambierone	$C_{52}H_{76}O_{18}S$	1021.4825	1038.5090	1043.4644
	38-dehydroxy-12,13-dihydro-44-methylgambierone	$C_{52}H_{78}O_{18}S$	1023.4982	1040.5247	1045.4801
	12,13-dihydro-44-methylgambierone	$C_{52}H_{80}O_{19}S$	1041.5088	1058.5353	1063.4907
**Type**	**Compound**	**Molecular formula**	**$[M+2H]^{2+}$**	**$[M+2NH_4]^{2+}$**	**$[M+2Na]^{2+}$**
Maitotoxins (8)	Maitotoxin 1	$C_{164}H_{258}O_{68}S_2$	1690.3122	1698.8255	1701.3032
	Maitotoxin disodium salt	$C_{164}H_{256}Na_2O_{68}S_2$	1712.2942	1720.8074	1723.2851
	Maitotoxin 4	$C_{157}H_{241}NO_{68}S_2$	1646.7473	1655.2605	1657.7382
	Maitotoxin 5	$C_{161}H_{252}O_{68}S_2$	1669.2888	1677.8020	1680.2797
	Maitotoxin 6	$C_{164}H_{256}O_{66}S$	1657.3235	1665.8367	1668.3144
	Maitotoxin 7	$C_{165}H_{258}O_{67}S$	1672.3288	1680.8420	1683.3197
	desulfo-MTX-1	$C_{164}H_{258}O_{65}S$	1650.3339	1658.8471	1661.3248
	didehydro-demethyl-desulfo-MTX-1	$C_{163}H_{254}O_{65}S$	1642.3182	1650.8315	1653.3092

Table S2. The differences between the precursor and fragment ions

x	Loss moiety	Description
0	-	$m_h = m_p$
18.0106	H_2O	m_p, m_h, same adduct type, loss one H_2O
79.9568	SO_3	m_p, m_h, same adduct type, loss one SO_3
97.9674	H_2O+SO_3	m_p, m_h, same adduct type, loss one H_2O and one SO_3

17.0265	NH_4-H	m_p: ammonium adduct, m_h: protonated adduct
35.0371	NH_4-H+H_2O	m_p: ammonium adduct, m_h: protonated adduct; loss one H_2O
96.9834	NH_4-H+SO_3	m_p: ammonium adduct, m_h: protonated adduct; loss one SO_3
114.9939	NH_4-H+H_2O+SO_3	m_p: ammonium adduct, m_h: protonated adduct; loss one H_2O and one SO_3
21.9820	Na-H	m_p: sodium adduct, m_h: protonated adduct
39.9925	Na-H+H_2O	m_p: sodium adduct, m_h: protonated adduct; loss one H_2O
101.9388	Na-H+SO_3	m_p: sodium adduct, m_h: protonated adduct; loss one SO_3
119.9493	Na-H+H_2O+SO_3	m_p: sodium adduct, m_h: protonated adduct; loss one H_2O and one SO_3

Table S3. The information on neutral loss moieties

Loss moiety	Calculated mass (Da)	Loss moiety	Calculated mass (Da)	Loss moiety	Calculated mass (Da)
H_2O	18.0106	NH_3+H_2O	35.0371	Na-H+H_2O	39.9926
$2H_2O$	36.0211	NH_3+$2H_2O$	53.0476	Na-H+$2H_2O$	58.0031
$3H_2O$	54.0317	NH_3+$3H_2O$	71.0582	Na-H+$3H_2O$	76.0137
$4H_2O$	72.0423	NH_3+$4H_2O$	89.0688	Na-H+$4H_2O$	94.0243
$5H_2O$	90.0528	NH_3+$5H_2O$	107.0793	Na-H+$5H_2O$	112.0348
$6H_2O$	108.0634	NH_3+$6H_2O$	125.0899	Na-H+$6H_2O$	130.0454
$7H_2O$	126.0740	NH_3+$7H_2O$	143.1005	Na-H+$7H_2O$	148.0560
$8H_2O$	144.0845	NH_3+$8H_2O$	161.1110	Na-H+$8H_2O$	166.0665
$9H_2O$	162.0951	NH_3+$9H_2O$	179.1216	Na-H+$9H_2O$	184.0771
$10H_2O$	180.1056	NH_3+$10H_2O$	197.1321	Na-H+$10H_2O$	202.0876
SO_3	79.9568	NH_3+SO_3	96.9833	Na-H+SO_3	101.9388
SO_3+H2O	97.9674	NH_3+SO_3+H2O	114.9939	Na-H+SO_3+H2O	119.9494
SO_3+$2H_2O$	115.9779	NH_3+SO_3+$2H_2O$	133.0044	Na-H+SO_3+$2H_2O$	137.9599
SO_3+$3H_2O$	133.9885	NH_3+SO_3+$3H_2O$	151.0150	Na-H+SO_3+$3H_2O$	155.9705
SO_3+$4H_2O$	151.9991	NH_3+SO_3+$4H_2O$	169.0256	Na-H+SO_3+$4H_2O$	173.9811
SO_3+$5H_2O$	170.0096	NH_3+SO_3+$5H_2O$	187.0361	Na-H+SO_3+$5H_2O$	191.9916
$2SO_3$	159.9136	NH_3+$2SO_3$	176.9401	Na-H+$2SO_3$	181.8956
$2SO_3$+H_2O	177.9242	NH_3+$2SO_3$+H_2O	194.9507	Na-H+$2SO_3$+H_2O	199.9062
$2SO_3$+$2H_2O$	195.9348	NH_3+$2SO_3$+$2H_2O$	212.9613	Na-H+$2SO_3$+$2H_2O$	217.9168
$2SO_3$+$3H_2O$	213.9453	NH_3+$2SO_3$+$3H_2O$	230.9718	Na-H+$2SO_3$+$3H_2O$	235.9273
$2SO_3$+$4H_2O$	231.9559	NH_3+$2SO_3$+$4H_2O$	248.9824	Na-H+$2SO_3$+$4H_2O$	253.9379
$2SO_3$+$5H_2O$	249.9665	NH_3+$2SO_3$+$5H_2O$	266.9930	Na-H+$2SO_3$+$5H_2O$	271.9485

Table S4. The information of aligned adducts in *Gambierdiscus caribaeus* GCBG01

	m/z	RT (min)	SO_3 loss	NH_3 loss	Proposed adduct type	Scores
1	864.4995	6.694	0	yes	$[M+NH_4]^+$	9
	846.4904	6.694	0	yes	$[M-H_2O+NH_4]^+$	8
	829.4646	6.694	0	no	$[M-H_2O+H]^+$	9
2	1351.5880	7.236	1	no	$[M+H]^+$	4
	1333.5770	7.259	1	no	$[M-H_2O+H]^+$	4
3	892.4955	7.498	0	yes	$[M+NH_4]^+$	10
	875.4673	7.498	0	no	$[M+H]^+$	10
	857.4580	7.505	0	no	$[M-H_2O+H]^+$	10
4	1076.5160	7.891	1	yes	$[M+NH_4]^+$	9
	1041.4790	7.891	1	no	$[M-H_2O+H]^+$	10
	1023.4670	7.891	1	no	$[M-2H_2O+H]^+$	8
5	894.4233	8.589	2	no	$[M+H]^+$	4
	814.4730	8.586	1	no	$[M-SO_3+H]^+$	6
	734.5092	8.597	0	no	$[M-2SO_3+H]^+$	7
6	1567.7420	8.673	1	yes	$[M+NH_4]^+$	4
	1550.7160	8.665	1	no	$[M+H]^+$	5
7	1292.8540	9.9	0	yes	$[M+NH_4]^+$	10
	1257.8150	9.9	0	no	$[M-H_2O+H]^+$	10

8	1510.7890	10.355	0	yes	$[M+NH_4]^+$	10
	1475.7500	10.355	0	no	$[M-H_2O+H]^+$	9
9	958.5256	11.495	1	no	$[M+H]^+$	7
	878.5662	11.495	0	no	$[M-SO_3+H]^+$	8
	860.5610	11.51	0	no	$[M-SO_3-H_2O+H]^+$	6
10	1045.7820	11.963	0	no	$[M+H]^+$	10
	1027.7740	11.970	0	no	$[M-H_2O+H]^+$	10
11	892.5516	20.481	1	yes	$[M+NH_4]^+/[M+H]^+$	3
	875.5229	20.481	1	no	$[M+H]^+/[M-NH_3+H]^+$	3
12	1042.5080	12.093	1	yes	$[M+NH_4]^+$	8
	1025.4820	12.086	1	no	$[M+H]^+$	14
	1007.4730	12.086	1	no	$[M-H_2O+H]^+$	15
	927.5153	12.086	0	no	$[M-SO_3-H_2O+H]^+$	9
	909.5059	12.093	0	no	$[M-SO_3-2H_2O+H]^+$	9

Table S5. The information of aligned adducts in *Gambierdiscus caribaeus* GCBG02

	m/z	RT (min)	SO_3 loss	NH_3 loss	Proposed adduct type	Scores
1	945.4172	6.071	1	yes	$[M+NH_4]^+$	5
	928.3914	6.06	1	no	$[M+H]^+$	4
	848.4337	6.058	0	no	$[M-SO_3+H]^+$	3
2	1122.6780	6.746	0	no	$[M+H]^+$	5
	1104.6650	6.738	0	no	$[M-H_2O+H]^+$	4
3	913.4258	7.64	1	yes	$[M+NH_4]^+$	7
	896.4001	7.639	1	no	$[M+H]^+$	8
	816.4419	7.639	0	no	$[M-SO_3+H]^+$	7
4	830.4612	7.835	1	no	$[M-H_2O+H]^+$	4
	768.5166	7.85	0	no	$[M-SO_3+H]^+$	3
	750.5039	7.842	0	no	$[M-SO_3-H_2O+H]^+$	6
5	1076.5150	7.888	1	yes	$[M+NH_4]^+$	10
	1041.4800	7.896	1	no	$[M-H_2O+H]^+$	9
	1023.4690	7.904	1	no	$[M-2H_2O+H]^+$	6
6	1497.8220	8.06	0	yes	$[M+NH_4]^+$	8
	1480.7930	8.06	0	no	$[M+H]^+$	9
7	894.4230	8.589	2	no	$[M+H]^+$	6
	814.4662	8.597	1	no	$[M-SO_3+H]^+$	6
	734.5099	8.597	0	no	$[M-2SO_3+H]^+$	7
8	1567.7410	8.65	1	yes	$[M+NH_4]^+$	8
	1550.7140	8.658	1	no	$[M+H]^+$	6
9	816.4818	9.267	1	no	$[M+H]^+$	6
	736.5250	9.275	0	no	$[M-SO_3+H]^+$	5
10	1292.8560	9.922	0	yes	$[M+NH_4]^+$	10
	1257.8160	9.912	0	no	$[M-H_2O+H]^+$	10
11	978.4783	10.684	1	no	$[M+H]^+$	10
	898.5219	10.684	0	no	$[M-SO_3+H]^+$	7
12	1058.5410	11.488	1	yes	$[M+H]^+$	4
	1041.5170	11.489	1	no	$[M-NH_3+H]^+$	9
13	1263.8280	11.818	0	no	$[M+H]^+$	7
	1245.8130	11.81	0	no	$[M-H_2O+H]^+$	7
14	977.5681	11.826	1	yes	$[M+NH_4]^+$	7
	960.5413	11.831	1	no	$[M+H]^+$	8
	880.5870	11.833	0	no	$[M-SO_3+H]^+$	6
	862.5744	11.833	0	no	$[M-SO_3-H_2O+H]^+$	6
15	923.5208	12.288	0	no	$[M+H]^+$	5
	905.5093	12.288	0	no	$[M-H_2O+H]^+$	4
	887.4998	12.288	0	no	$[M-2H_2O+H]^+$	6
16	738.4499	19.762	1	yes	$[M+NH_4]^+$	4
	721.4221	19.755	1	no	$[M+H]^+$	3
17	988.5882	19.988	1	yes	$[M+NH_4]^+$	8
	935.5395	19.988	1	no	$[M-2H_2O+H]^+$	7
	855.5797	19.988	0	no	$[M-SO_3-2H_2O+H]^+$	5
18	1042.5090	12.102	1	yes	$[M+NH_4]^+$	4
	1025.4820	12.102	1	no	$[M+H]^+$	15
	1007.4720	12.11	1	no	$[M-H_2O+H]^+$	15
	927.5144	12.11	0	no	$[M-SO_3-H_2O+H]^+$	10
	909.5049	12.11	0	no	$[M-SO_3-2H_2O+H]^+$	7

Table S6. The information of aligned adducts in *Gambierdiscus caribaeus* S4

	m/z	RT (min)	SO_3 loss	NH_3 loss	Proposed adduct type	Scores
1	846.3799	6.793	0	yes	$[M+NH_4]^+$	10

	829.3553	6.791	0	no	$[M+H]^+$	5
2	913.4265	7.644	1	yes	$[M+NH_4]^+$	6
	896.4008	7.644	1	no	$[M+H]^+$	7
	816.4420	7.644	0	no	$[M-SO_3+H]^+$	7
3	830.4631	7.834	1	no	$[M+H]^+$	4
	750.5045	7.84	0	no	$[M-SO_3+H]^+$	5
4	1497.8210	8.047	0	yes	$[M+NH_4]^+$	7
	1480.7910	8.053	0	no	$[M+H]^+$	8
5	1567.7410	8.659	1	yes	$[M+NH_4]^+$	6
	1550.7150	8.653	1	no	$[M+H]^+$	4
6	1292.8540	9.903	0	yes	$[M+NH_4]^+$	4
	1257.8170	9.903	0	no	$[M-H_2O+H]^+$	5
7	958.5261	11.188	1	no	$[M+H]^+$	8
	860.5576	11.182	0	no	$[M-SO_3-H_2O+H]^+$	8
8	1058.5410	11.462	1	yes	$[M+H]^+$	4
	1041.4760	11.504	1	no	$[M-NH_3+H]^+$	5
9	958.5577	12.286	0	yes	$[M+NH_4]^+$	7
	923.5206	12.28	0	no	$[M+H]^+$	4
	905.5102	12.28	0	no	$[M-H_2O+H]^+$	4
10	1042.5090	12.089	1	yes	$[M+NH_4]^+$	4
	1025.4810	12.095	1	no	$[M+H]^+$	17
	1007.4730	12.089	1	no	$[M-H_2O+H]^+$	12
	962.5529	12.089	0	yes	$[M-H_2O+NH_4]^+$	10
	927.5150	12.095	0	no	$[M-SO_3-H_2O+H]^+$	11
	909.5035	12.089	0	no	$[M-SO_3-2H_2O+H]^+$	10

Table S7. The information of ten shared polyether compounds across three *G.caribaeus* strains.

	m/z	RT (min)	SO_3 loss	NH_3 loss	Scores	
1	700.4966	11.053	0	no	7	*G.caribaeus* GCBG01
	700.4967	11.059	0	no	8	*G.caribaeus* GCBG02
	700.4960	11.045	0	no	9	*G.caribaeus* S4
2	708.3837	9.132	0	yes	7	*G.caribaeus* GCBG01
	708.3834	9.151	0	yes	5	*G.caribaeus* GCBG02
	708.3835	9.153	0	yes	3	*G.caribaeus* S4
3	779.3898	8.513	0	no	8	*G.caribaeus* GCBG01
	779.3893	8.521	0	no	6	*G.caribaeus* GCBG02
	779.3876	8.517	0	no	7	*G.caribaeus* S4
4	978.4600	6.942	0	no	6	*G.caribaeus* GCBG01
	978.4600	6.940	0	no	9	*G.caribaeus* GCBG02
	978.4604	6.948	0	no	10	*G.caribaeus* S4
5	999.7406	12.477	0	no	10	*G.caribaeus* GCBG01
	999.7402	12.487	0	no	10	*G.caribaeus* GCBG02
	999.7390	12.475	0	no	10	*G.caribaeus* S4
6	1292.8540	9.900	0	yes	10	*G.caribaeus* GCBG01
	1292.8560	9.922	0	yes	10	*G.caribaeus* GCBG02
	1292.8540	9.903	0	yes	8	*G.caribaeus* S4
7	783.4376	21.869	1	no	3	*G.caribaeus* GCBG01
	783.4379	21.867	1	no	4	*G.caribaeus* GCBG02
	783.4368	21.722	1	no	3	*G.caribaeus* S4
8	859.5289	22.163	1	no	3	*G.caribaeus* GCBG01
	859.5298	22.192	1	no	6	*G.caribaeus* GCBG02
	859.5276	22.005	1	no	3	*G.caribaeus* S4
9	1567.7420	8.673	1	yes	4	*G.caribaeus* GCBG01
	1567.7410	8.650	1	yes	8	*G.caribaeus* GCBG02
	1567.7410	8.659	1	yes	6	*G.caribaeus* S4
10	1025.4820	12.086	1	no	14	*G.caribaeus* GCBG01
	1025.4820	12.102	1	no	15	*G.caribaeus* GCBG02
	1025.4810	12.095	1	no	17	*G.caribaeus* S4

Table S8. The cellular level of gambierone in three *Gambierdiscus* strains.

Compound	Cellular toxin level (mean ± SD, pg/cell)		
	G. caribaeus GCBG01	*G. caribaeus* GCBG02	*G. caribaeus* S4
gambierone	119.38 ± 25.27^a	72.48 ± 17.35^b	26.23 ± 2.51^c

Note: The superscripts a, b, and c are significant homogenous subsets of means between groups. Different superscripts were statistically significant at the 0.05 level by the one-way ANOVA and Duncan's test.

Table S9 The empirical formula of ion at *m/z* 1058 (11.4 min) prediction

Formula	m/z	Adduct type	RDB	Mass error (ppm)

C56H83NO16S	1058.5505	$[M+H]^+$	16	-8.8
C52H83NO19S	1058.5353	$[M+H]^+$	12	5.6
C63H79NO19S	1058.5447	$[M+H]^+$	25	-3.3
C45H87NO24S	1058.5412	$[M+H]^+$	3	0

Note: The elements and limits allowed included C, 5-100; H, 0-200; O, 0-100; N, 0-1; and S, 0-1 (mass error < 10 ppm). The ring double bond (RDB)equivalents of each compound were calculated. Four compounds were found who met those conditions.

Table S10. MSDIAL 4.9 parameters for LC-HRMS/MS datasets processing

Data collection		
MS^1 tolerance	0.01 Da	
MS^2 tolerance	0.025 Da	
Retention time begin	0 min	
Retention time end	30 min	
MS^1 mass range	50-2000 Da	
MS/MS mass range	50-2000 Da	
Maximum charged number	3	
Number of threads	3	
Peak detection		
Minimum peak height	50 amplitudes	
Mass slice width	0.1 Da	
Smoothing method	Linear weighted moving average	
Smoothing level	3 scans	
Minimum peak width	5 scans	
MS^2 detection		
Sigma window value	0.5	
MS/MS abundance cut off	5 amplitudes	
Keep the isotopic ions until	0.5 Da	
MS/MS identification setting		
Retention time tolerance	0.1 min	
Accurate mass tolerance (MS^1)	0.01 Da	
Accurate mass tolerance (MS^2)	0.01 Da	
Identification score cut off	85%	
Adduct		
Molecular species	charge	Accurate mass (Da)
$[M+H]^+$	1	+1.007276
$[M+NH_4]^+$	1	+18.033823
$[M+Na]^+$	1	+22.989218
$[M+H-H_2O]^+$	1	-17.002191
$[M+H-2H_2O]^+$	1	-30.012756
$[M+2Na-H]^+$	1	+44.97116
$[2M+H]^+$	1	+1.007276
$[2M+NH_4]^+$	1	+18.033823
$[2M+Na]^+$	1	+22.989218
$[M+2H]^{2+}$	2	+1.007276
$[M+H+NH_4]^{2+}$	2	+9.52055

$[M+H+Na]^{2+}$	2	+11.998247
$[M+2Na]^{2+}$	2	+22.989218
$[M+3H]^{3+}$	3	+1.007276
$[M+2H+Na]^{3+}$	3	+15.76619
$[M+H+2Na]^{3+}$	3	+22.989218
Alignment		
Retention time tolerance	0.1 min	
MS^1 tolerance	0.02 Da	
Retention time factor	0.5	
MS^1 factor	0.5	

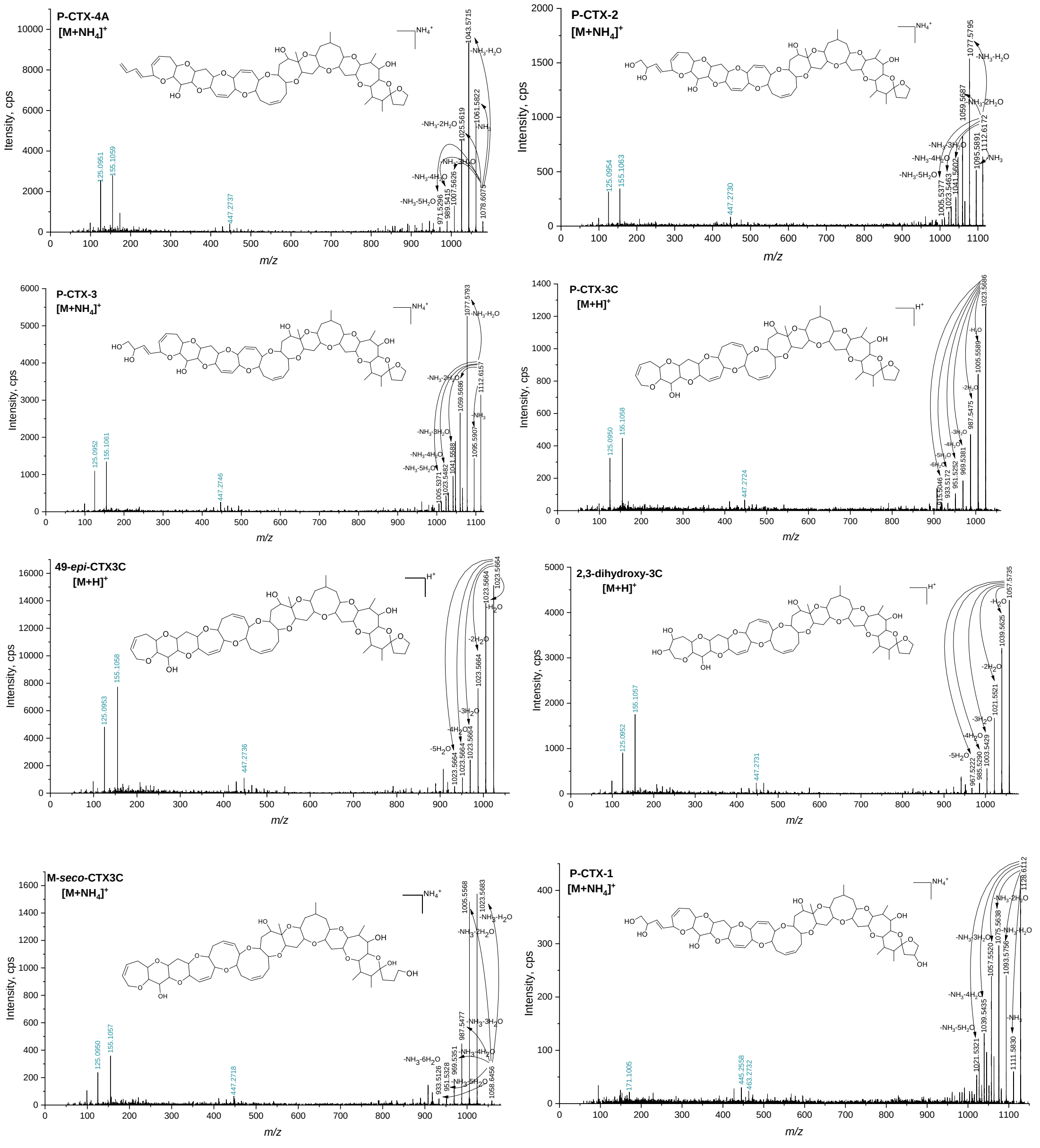

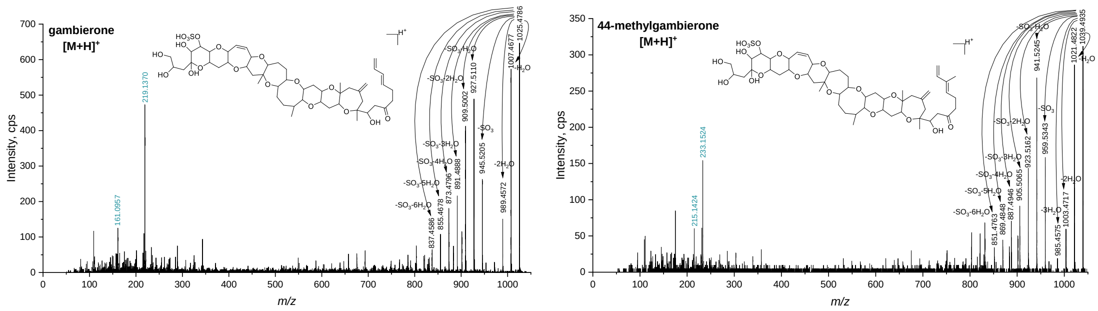

Figure S1. Fragment ion spectra (MS^2, MS/MS) of $[M + NH_4]^+$ ions of P-CTX-4A (P-CTX I type), P-CTX-2 (P-CTX I type), P-CTX-3 (P-CTX I type), P-CTX-1 (P-CTX I type), and M-*seco*-CTX3C (P-CTX II type), $[M + H]^+$ ions of P-CTX-3C (P-CTX II type), 49-*epi*-3C (P-CTX II type), 2,3-dihydroxy-3C (P-CTX II type), gambierone, and 44-methylgambierone. Diagnostic ions were highlighted in cyan.

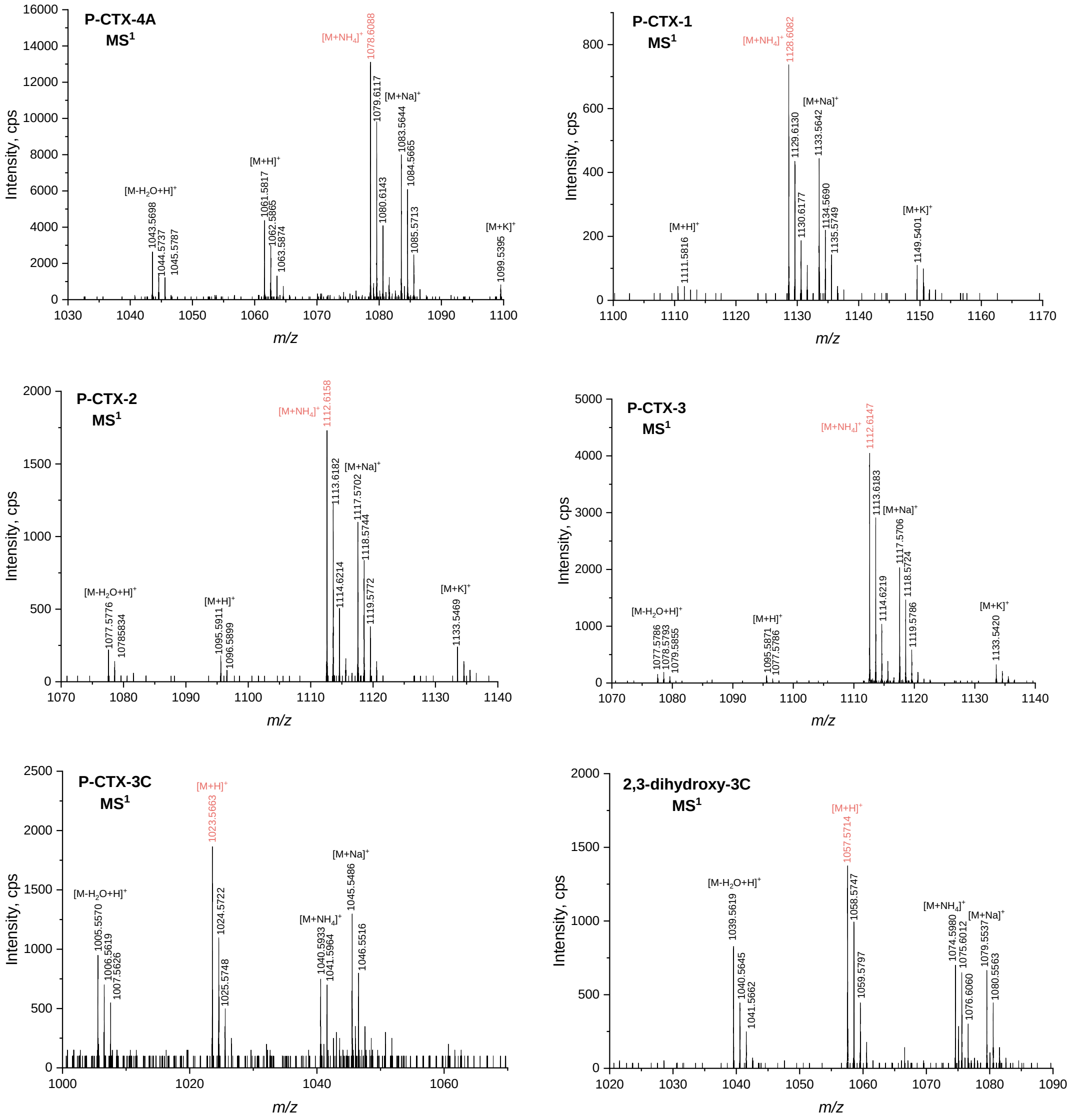

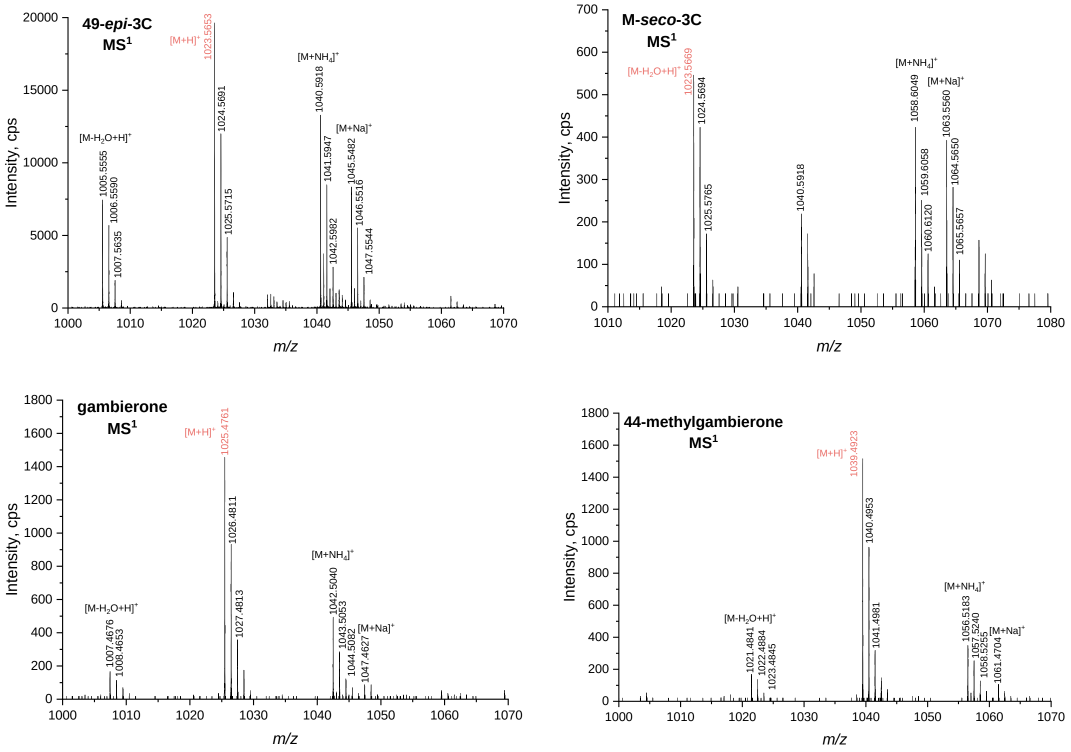

Figure S2. Full-scan mass spectra (MS[1]) of P-CTX-4A, P-CTX-1, P-CTX-2, P-CTX-3, P-CTX-3C, 2,3-dihydroxy-3C, 49-*epi*-3C, M-*seco*-3C, gambierone, and 44-methylgambierone. The ions with the highest intensity were shown in red colour.

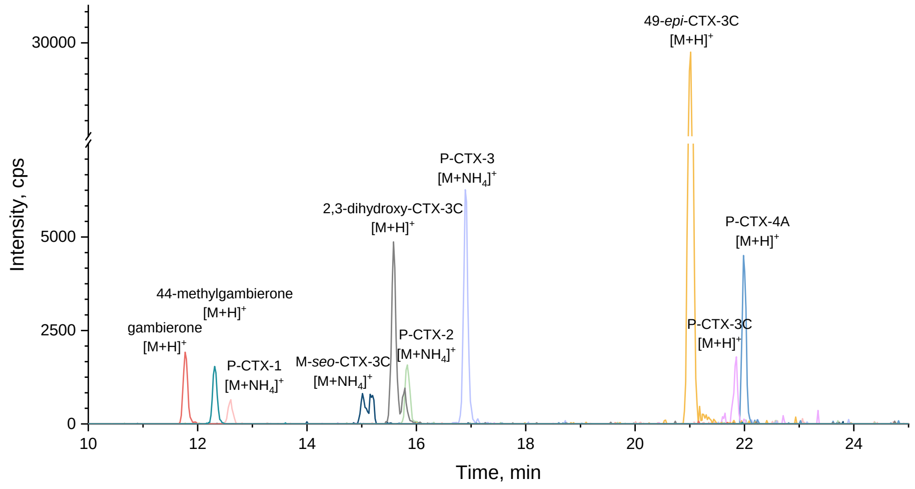

Figure S3. Extraction ion chromatograms (XICs) of gambierone (100 ng/mL), 44-methylgambierone (100 ng/mL), P-CTX-1 (20 ng/mL), M-*seco*-3C (100 ng/mL), 2,3-dihydroxy-3C (100 ng/mL), P-CTX-2 (80 ng/mL), P-CTX-3 (80 ng/mL), 49-*epi*-3C (100 ng/mL), P-CTX-3C (100 ng/mL), and P-CTX-4A (100 ng/mL).

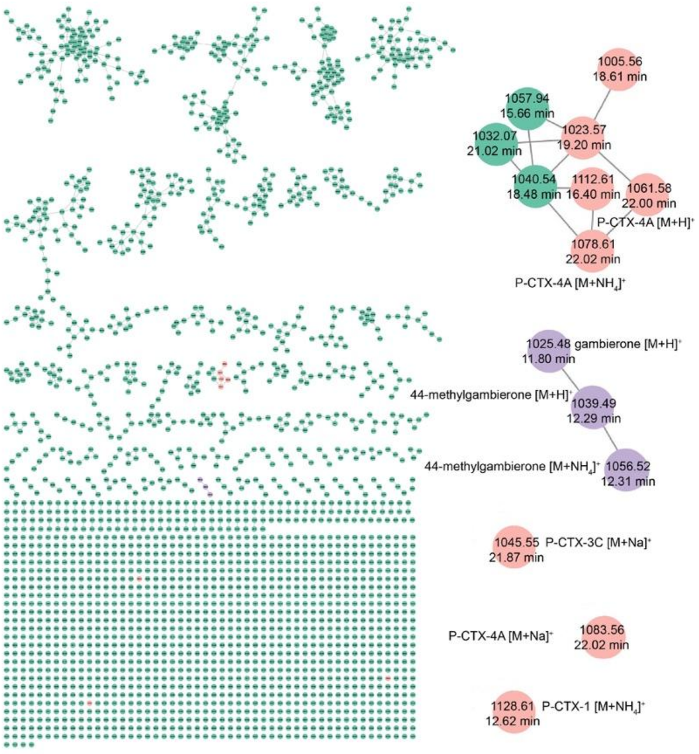

Figure S4. Molecular network (MN) of eight P-CTXs (*i.e.*, P-CTX-1, P-CTX-2, P-CTX-3, P-CTX-4A, P-CTX-3C, 2,3-dihydroxy-CTX-3C, 49-*epi*-CTX-3C, and M-*seco*-CTX-3C) and two gambierones (*i.e.*, gambierone, 44-methylgambierone). MN was created with the following parameters: cosine score of 0.7, minimum of 6 common fragment ions and a TopK set at 1000. Each node contained the *m/z* of the precursor ion and P-CTXs and gambierones were highlighted by pink and purple, respectively.

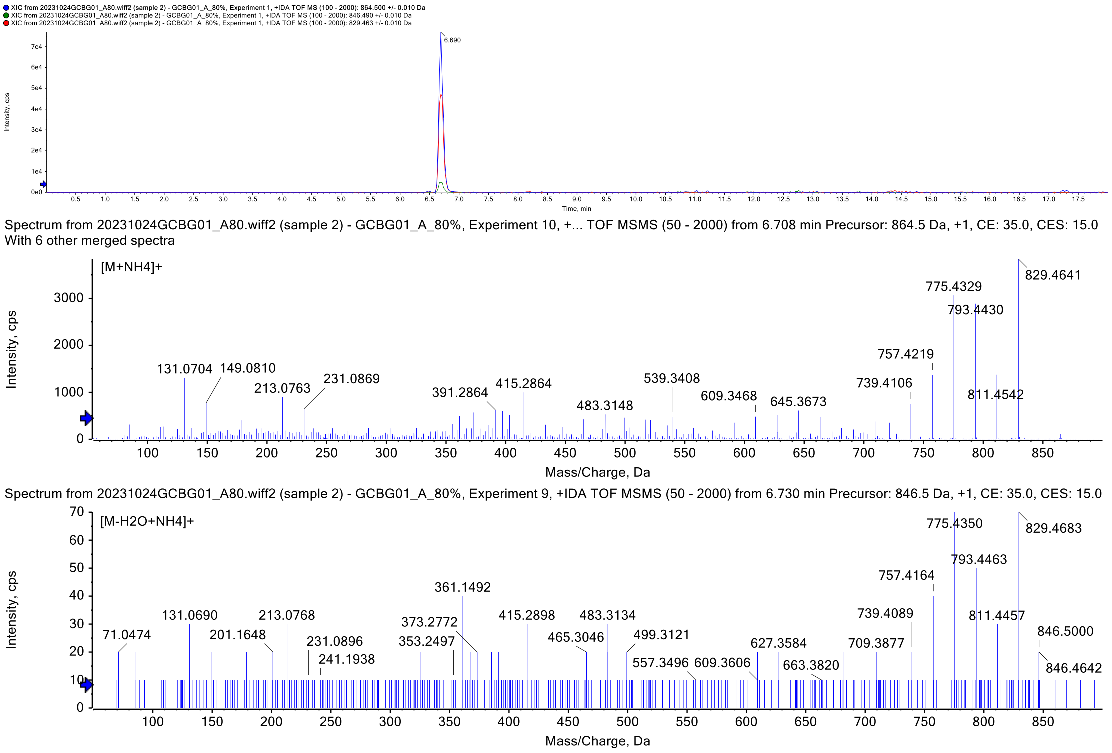

Spectrum from 20231024GCBG01_A80.wiff2 (sample 2) - GCBG01_A_80%, Experiment 9, +IDA TOF MSMS (50 - 2000) from 6.707 min Precursor: 829.5 Da, +1, CE: 35.0, CES: 15.0
With 4 other merged spectra

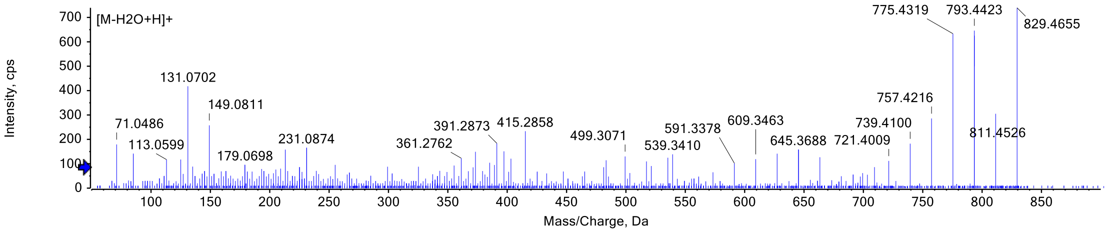

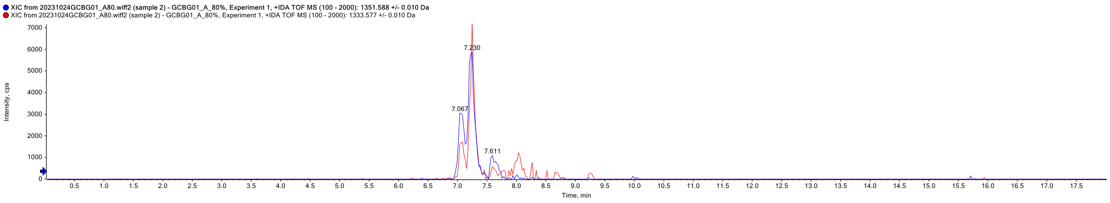

Spectrum from 20231024GCBG01_A80.wiff2 (sample 2) - GCBG01_A_80%, Experiment 11, +... TOF MSMS (50 - 2000) from 7.243 min Precursor: 1351.6 Da, +1, CE: 35.0, CES: 15.0
With 8 other merged spectra

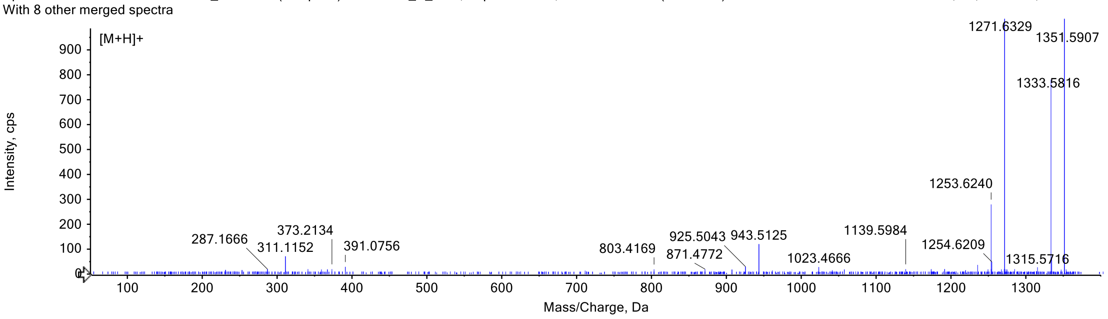

Spectrum from 20231024GCBG01_A80.wiff2 (sample 2) - GCBG01_A_80%, Experiment 10, +... TOF MSMS (50 - 2000) from 7.056 min Precursor: 1333.6 Da, +1, CE: 35.0, CES: 15.0
With 3 other merged spectra

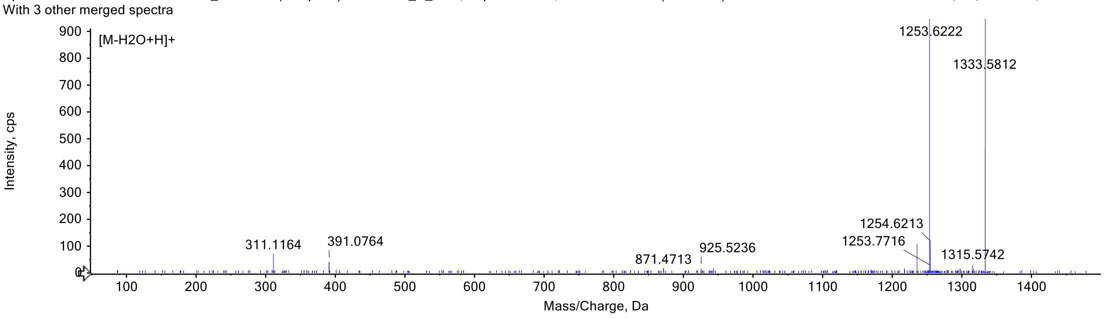

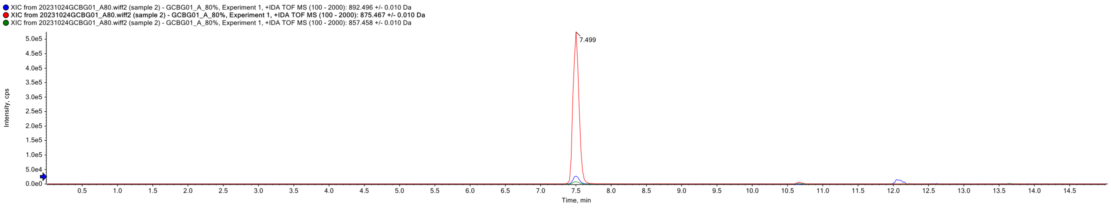

Spectrum from 20231024GCBG01_C80.wiff2 (sample 1) - GCBG01_C_80%, Experiment 11, +... TOF MSMS (50 - 2000) from 7.607 min Precursor: 892.5 Da, +1, CE: 35.0, CES: 15.0
With 6 other merged spectra

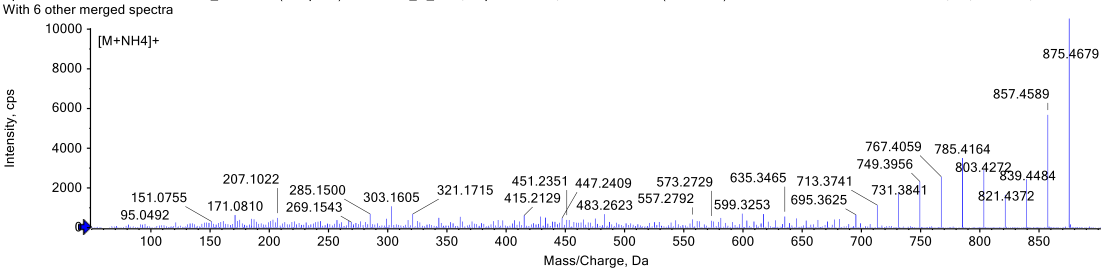

Spectrum from 20231024GCBG01_C80.wiff2 (sample 1) - GCBG01_C_80%, Experiment 8, +IDA TOF MSMS (50 - 2000) from 7.487 min Precursor: 875.5 Da, +1, CE: 35.0, CES: 15.0
With 6 other merged spectra

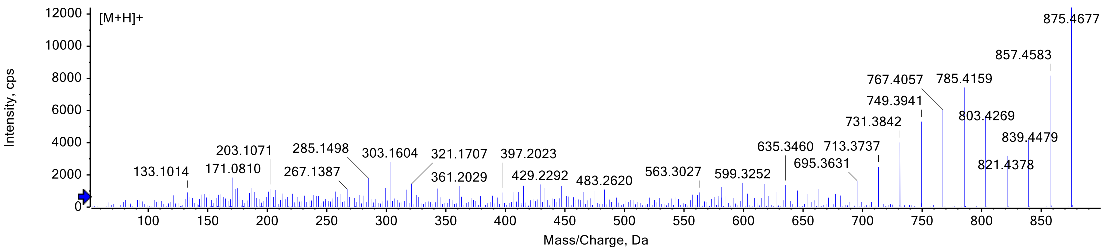

Spectrum from 20231024GCBG01_C80.wiff2 (sample 1) - GCBG01_C_80%, Experiment 9, +IDA TOF MSMS (50 - 2000) from 7.580 min Precursor: 857.5 Da, +1, CE: 35.0, CES: 15.0
With 1 other merged spectrum

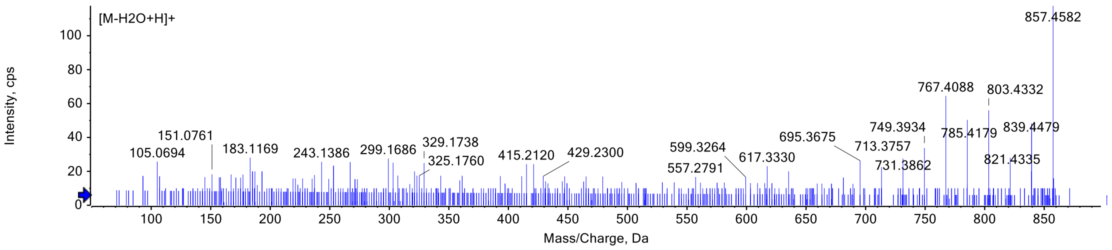

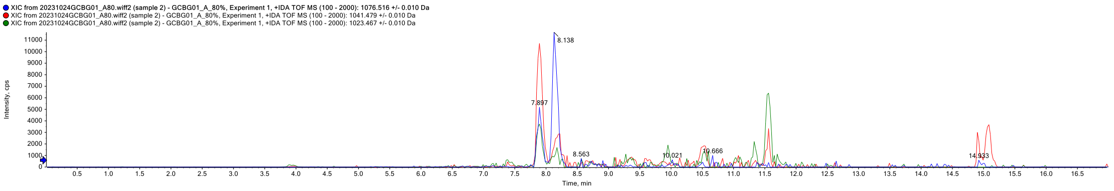

Spectrum from 20231024GCBG01_A80.wiff2 (sample 2) - GCBG01_A_80%, Experiment 11, +... TOF MSMS (50 - 2000) from 7.890 min Precursor: 1076.5 Da, +1, CE: 35.0, CES: 15.0
With 2 other merged spectra

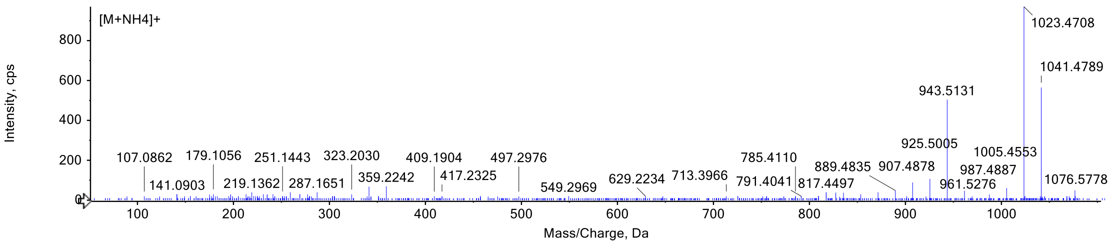

Spectrum from 20231024GCBG01_A80.wiff2 (sample 2) - GCBG01_A_80%, Experiment 11, +... TOF MSMS (50 - 2000) from 7.959 min Precursor: 1041.5 Da, +1, CE: 35.0, CES: 15.0
With 1 other merged spectrum

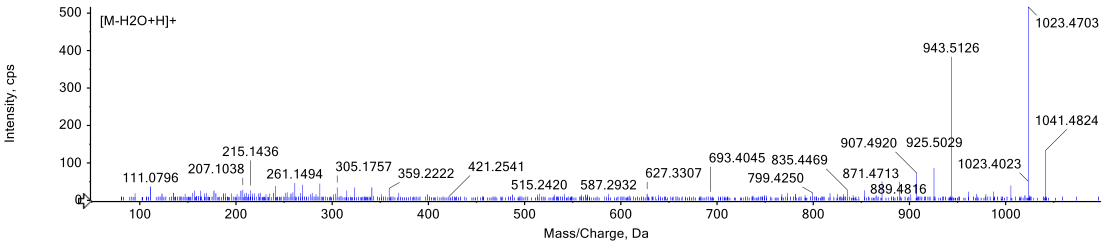

Spectrum from 20231024GCBG01_A80.wiff2 (sample 2) - GCBG01_A_80%, Experiment 10, +... TOF MSMS (50 - 2000) from 7.934 min Precursor: 1023.5 Da, +1, CE: 35.0, CES: 15.0

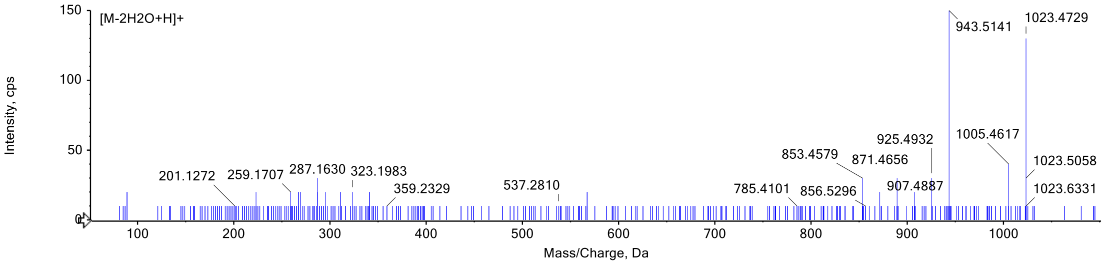

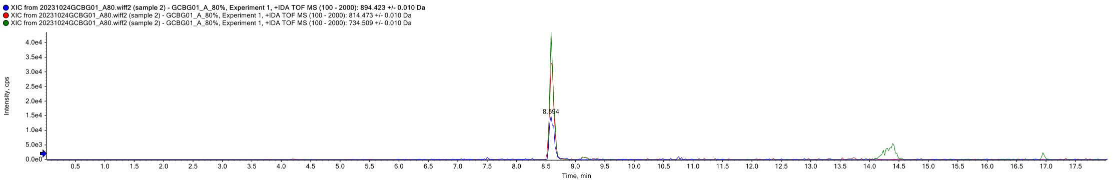

Spectrum from 20231024GCBG01_B80.wiff2 (sample 1) - GCBG01_B_80%, Experiment 11, +... TOF MSMS (50 - 2000) from 8.596 min Precursor: 894.4 Da, +1, CE: 35.0, CES: 15.0

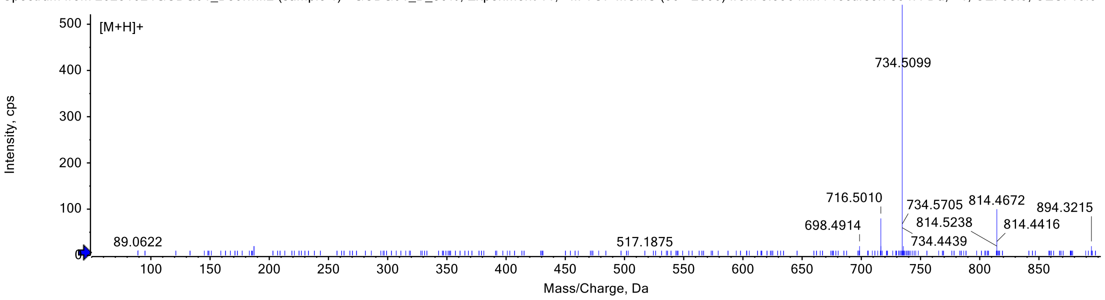

Spectrum from 20231024GCBG01_B80.wiff2 (sample 1) - GCBG01_B_80%, Experiment 10, +... TOF MSMS (50 - 2000) from 8.617 min Precursor: 814.5 Da, +1, CE: 35.0, CES: 15.0
With 1 other merged spectrum

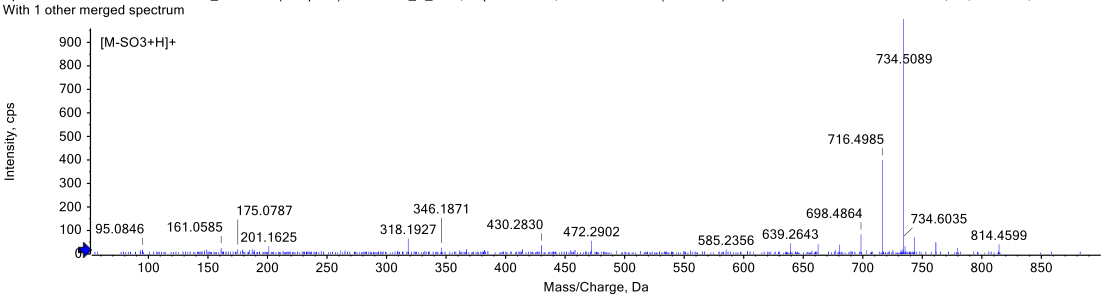

Spectrum from 20231024GCBG01_B80.wiff2 (sample 1) - GCBG01_B_80%, Experiment 9, +IDA TOF MSMS (50 - 2000) from 8.593 min Precursor: 734.5 Da, +1, CE: 35.0, CES: 15.0

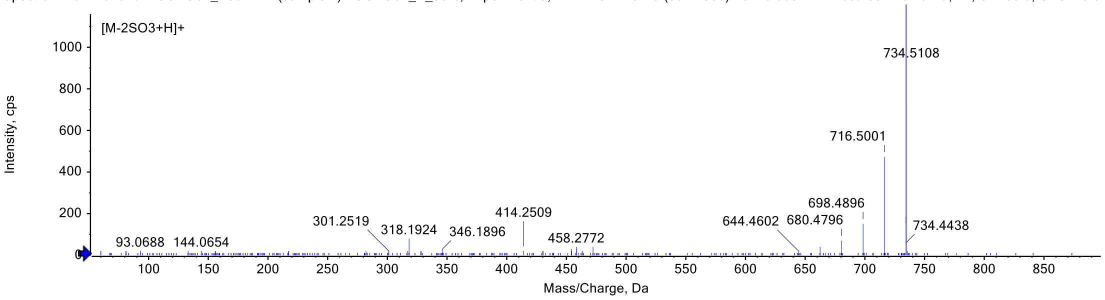

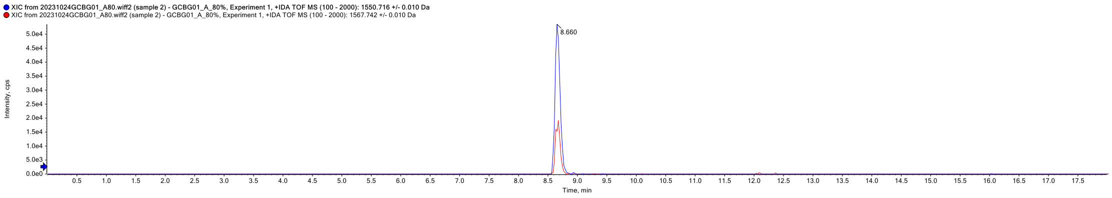

Spectrum from 20231024GCBG01_A80.wiff2 (sample 2) - GCBG01_A_80%, Experiment 11, +... TOF MSMS (50 - 2000) from 8.695 min Precursor: 1567.7 Da, +1, CE: 35.0, CES: 15.0
With 1 other merged spectrum

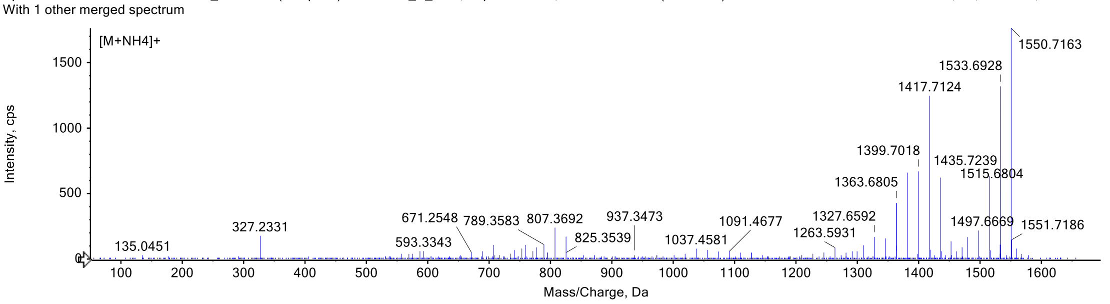

Spectrum from 20231024GCBG01_A80.wiff2 (sample 2) - GCBG01_A_80%, Experiment 11, +... TOF MSMS (50 - 2000) from 8.718 min Precursor: 1550.7 Da, +1, CE: 35.0, CES: 15.0
With 4 other merged spectra

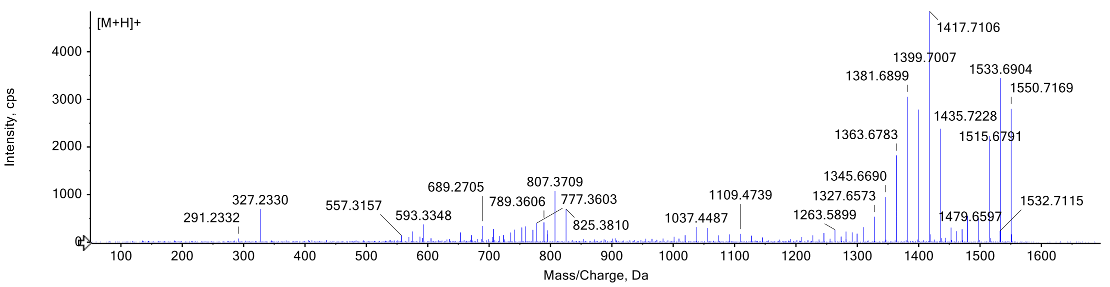

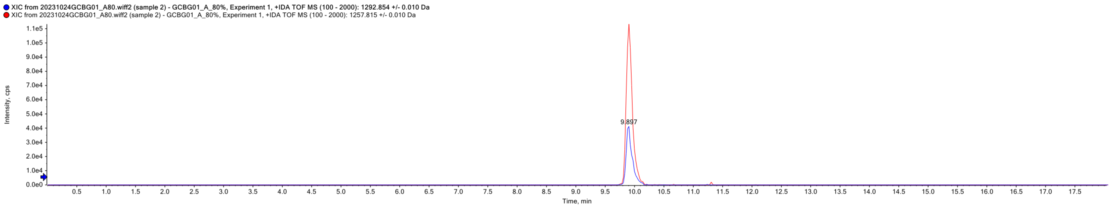

Spectrum from 20231024GCBG01_A80.wiff2 (sample 2) - GCBG01_A_80%, Experiment 11, +... TOF MSMS (50 - 2000) from 9.922 min Precursor: 1292.9 Da, +1, CE: 35.0, CES: 15.0
With 4 other merged spectra

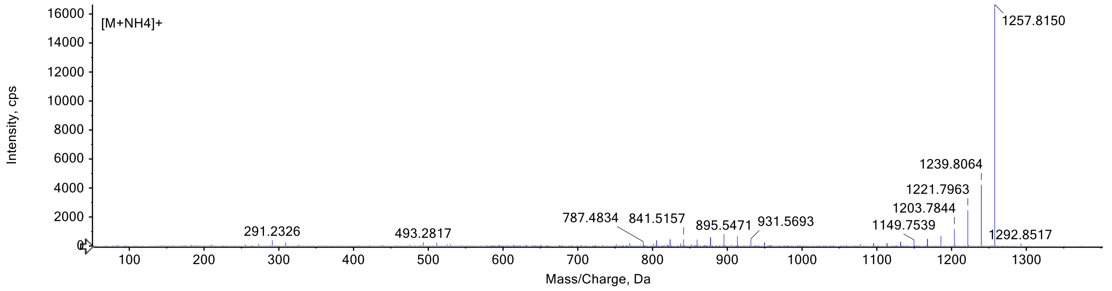

Spectrum from 20231024GCBG01_A80.wiff2 (sample 2) - GCBG01_A_80%, Experiment 9, +I... TOF MSMS (50 - 2000) from 9.942 min Precursor: 1257.8 Da, +1, CE: 35.0, CES: 15.0
With 9 other merged spectra

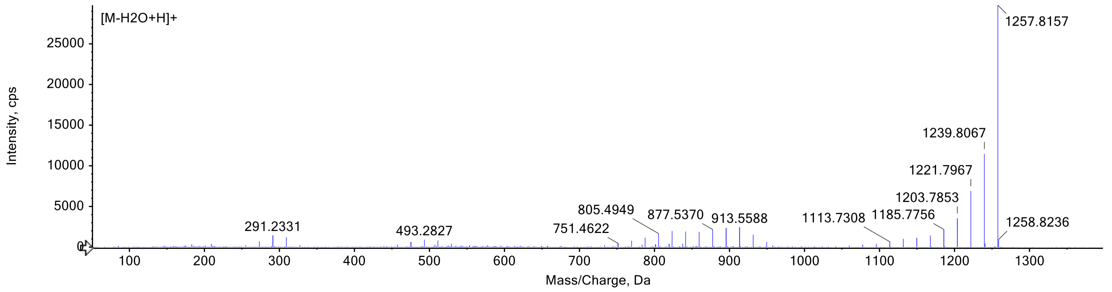

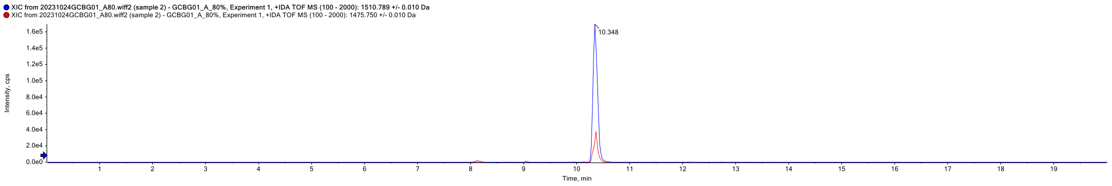

Spectrum from 20231024GCBG01_A80.wiff2 (sample 2) - GCBG01_A_80%, Experiment 11, +...OF MSMS (50 - 2000) from 10.407 min Precursor: 1510.8 Da, +1, CE: 35.0, CES: 15.0
With 5 other merged spectra

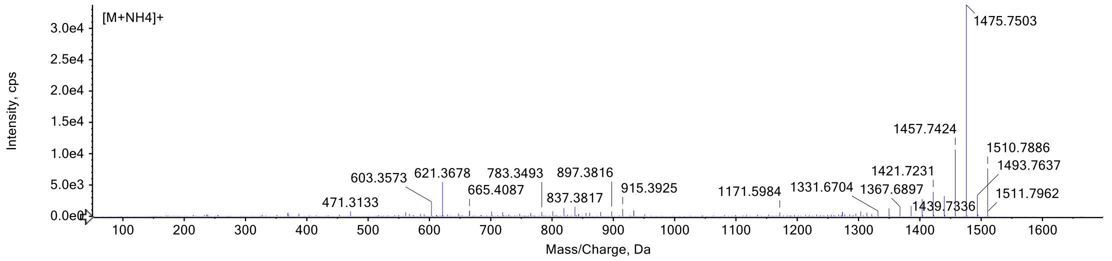

Spectrum from 20231024GCBG01_A80.wiff2 (sample 2) - GCBG01_A_80%, Experiment 10, +...OF MSMS (50 - 2000) from 10.383 min Precursor: 1475.8 Da, +1, CE: 35.0, CES: 15.0
With 1 other merged spectrum

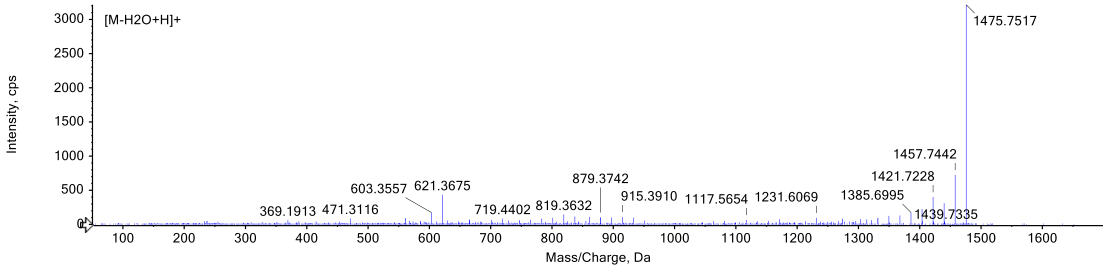

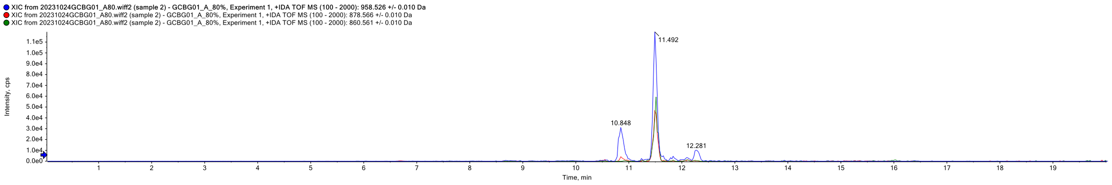

Spectrum from 20231024GCBG01_A80.wiff2 (sample 2) - GCBG01_A_80%, Experiment 11, +... TOF MSMS (50 - 2000) from 11.439 min Precursor: 958.5 Da, +1, CE: 35.0, CES: 15.0

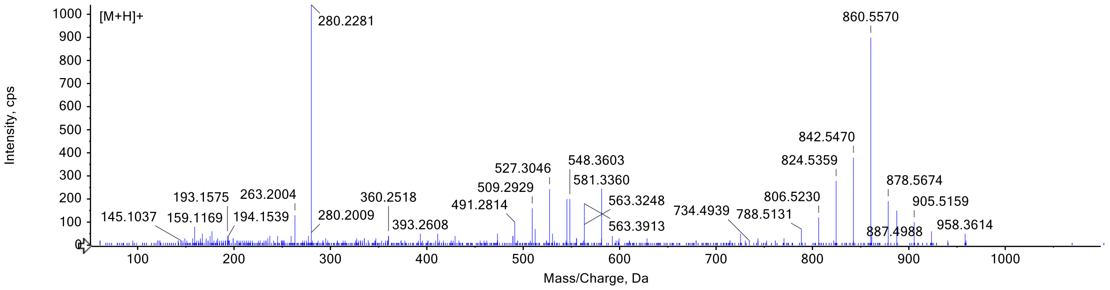

Spectrum from 20231024GCBG01_A80.wiff2 (sample 2) - GCBG01_A_80%, Experiment 10, +... TOF MSMS (50 - 2000) from 11.507 min Precursor: 878.6 Da, +1, CE: 35.0, CES: 15.0

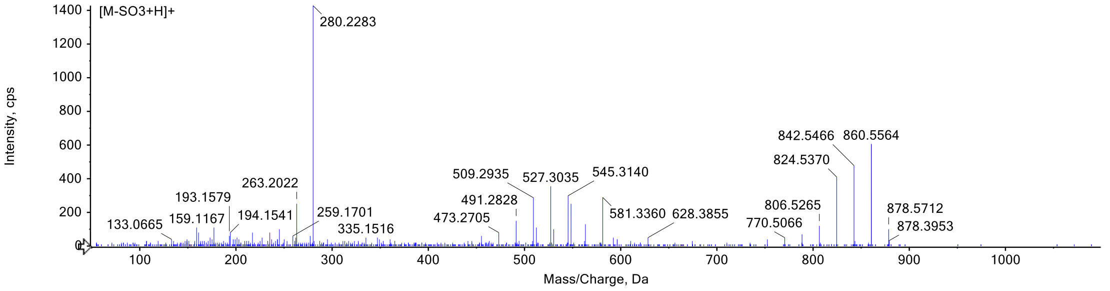

Spectrum from 20231024GCBG01_A80.wiff2 (sample 2) - GCBG01_A_80%, Experiment 10, +... TOF MSMS (50 - 2000) from 11.530 min Precursor: 860.6 Da, +1, CE: 35.0, CES: 15.0
With 1 other merged spectrum

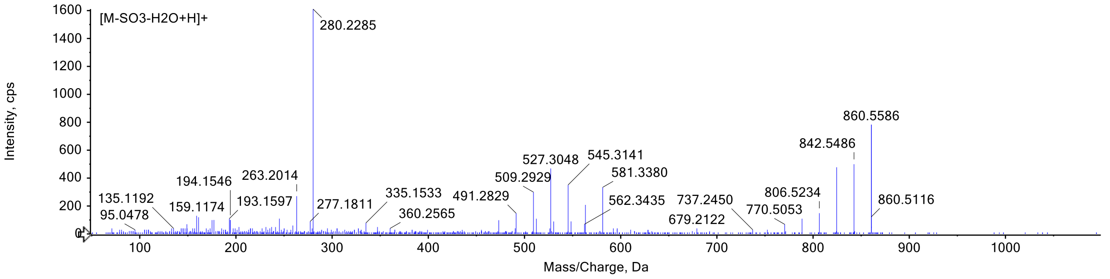

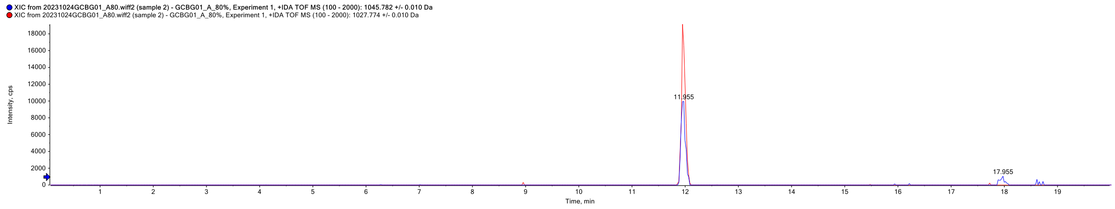

Spectrum from 20231024GCBG01_C80.wiff2 (sample 1) - GCBG01_C_80%, Experiment 11, +...OF MSMS (50 - 2000) from 11.966 min Precursor: 1045.8 Da, +1, CE: 35.0, CES: 15.0

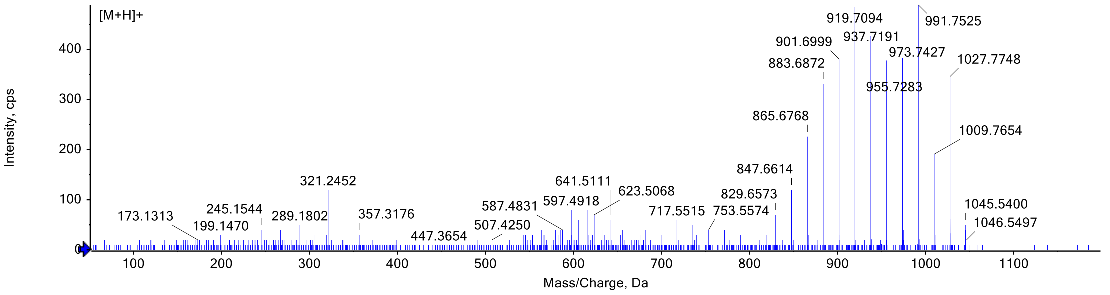

Spectrum from 20231024GCBG01_C80.wiff2 (sample 1) - GCBG01_C_80%, Experiment 10, +...OF MSMS (50 - 2000) from 11.964 min Precursor: 1027.8 Da, +1, CE: 35.0, CES: 15.0
With 1 other merged spectrum

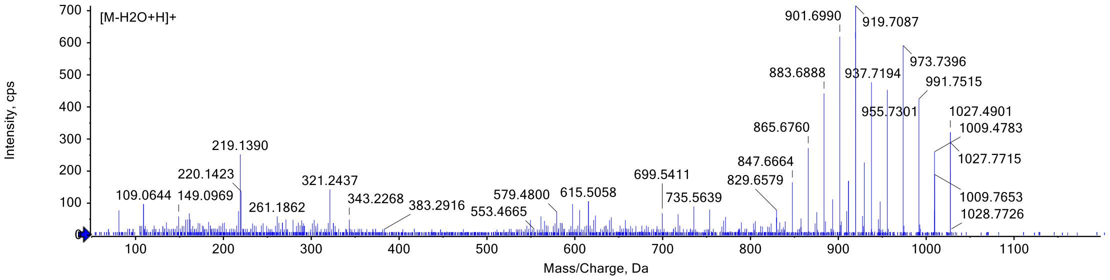

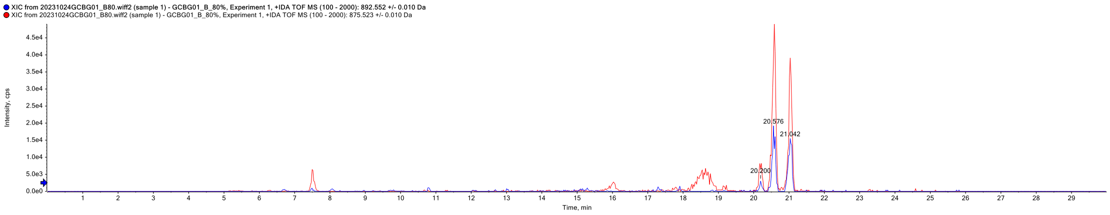

Spectrum from 20231024GCBG01_B80.wiff2 (sample 1) - GCBG01_B_80%, Experiment 6, +I... TOF MSMS (50 - 2000) from 21.118 min Precursor: 892.6 Da, +1, CE: 35.0, CES: 15.0
With 12 other merged spectra

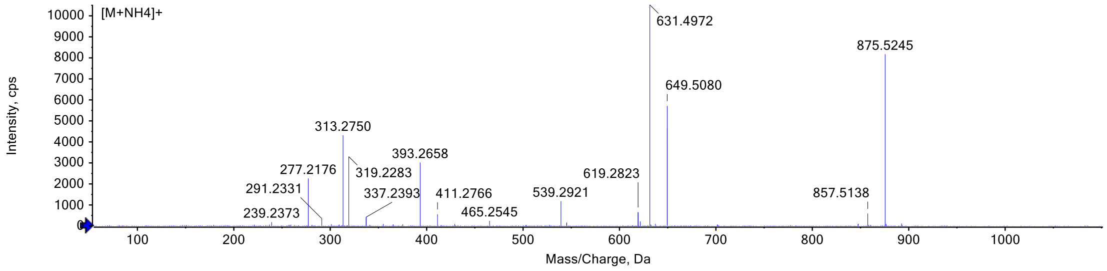

Spectrum from 20231024GCBG01_B80.wiff2 (sample 1) - GCBG01_B_80%, Experiment 6, +I... TOF MSMS (50 - 2000) from 20.159 min Precursor: 875.5 Da, +1, CE: 35.0, CES: 15.0
With 8 other merged spectra

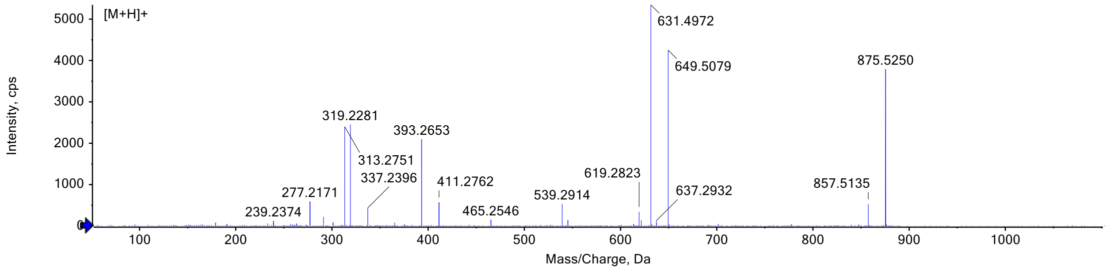

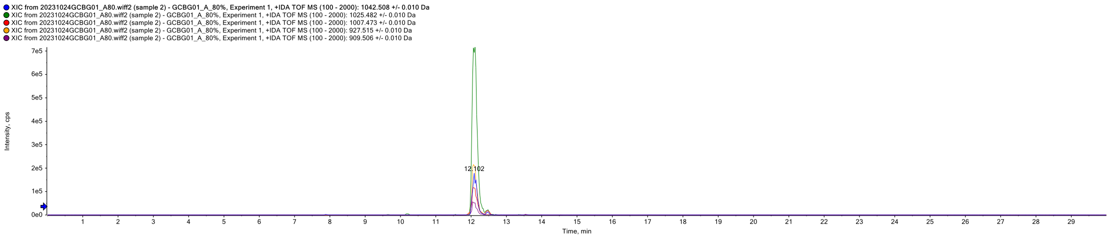

Spectrum from 20231024GCBG01_A80.wiff2 (sample 2) - GCBG01_A_80%, Experiment 10, +...OF MSMS (50 - 2000) from 12.105 min Precursor: 1042.5 Da, +2, CE: 35.0, CES: 15.0
With 8 other merged spectra

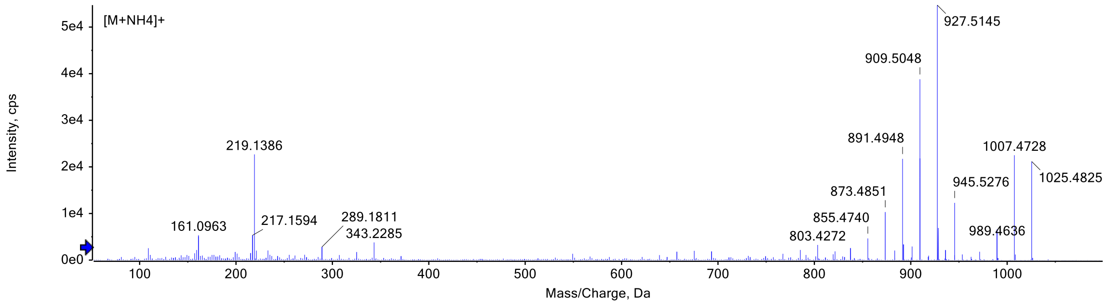

Spectrum from 20231024GCBG01_A80.wiff2 (sample 2) - GCBG01_A_80%, Experiment 9, +I...TOF MSMS (50 - 2000) from 12.057 min Precursor: 1025.5 Da, +1, CE: 35.0, CES: 15.0
With 10 other merged spectra

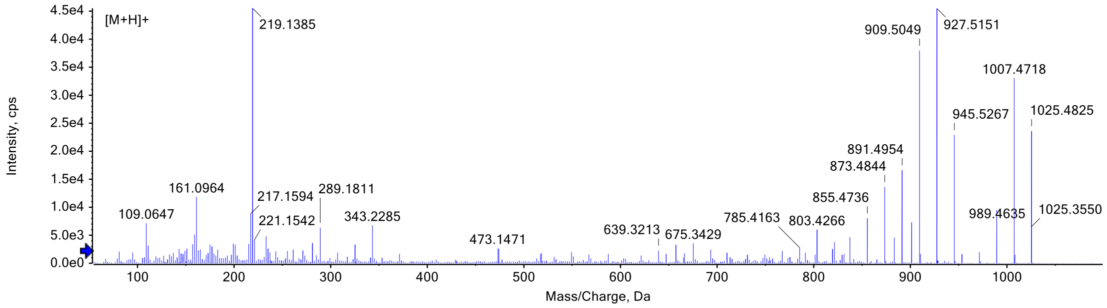

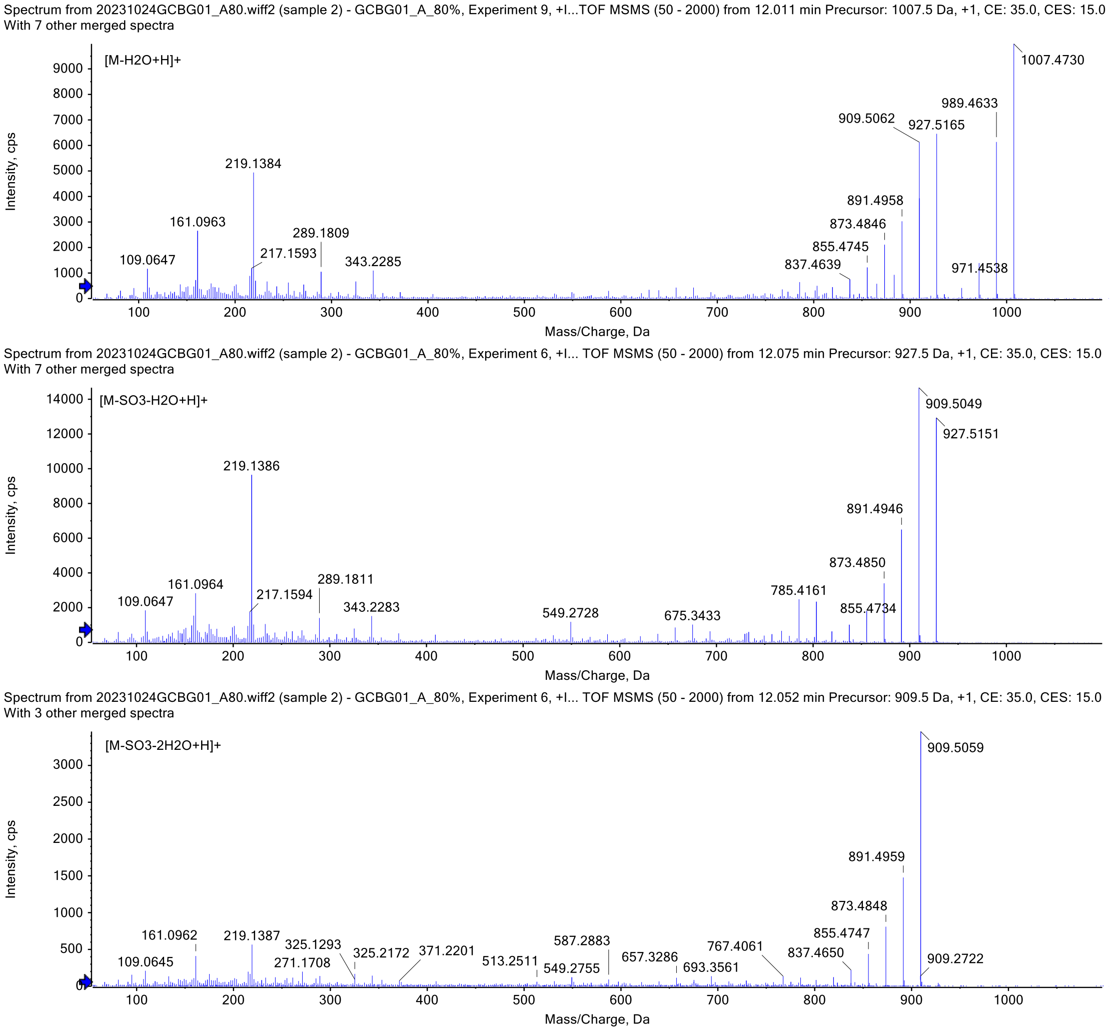

Figure S5. The MS^1 and MS^2 spectra of aligned adducts in *Gambierdiscus caribaeus* GCBG01

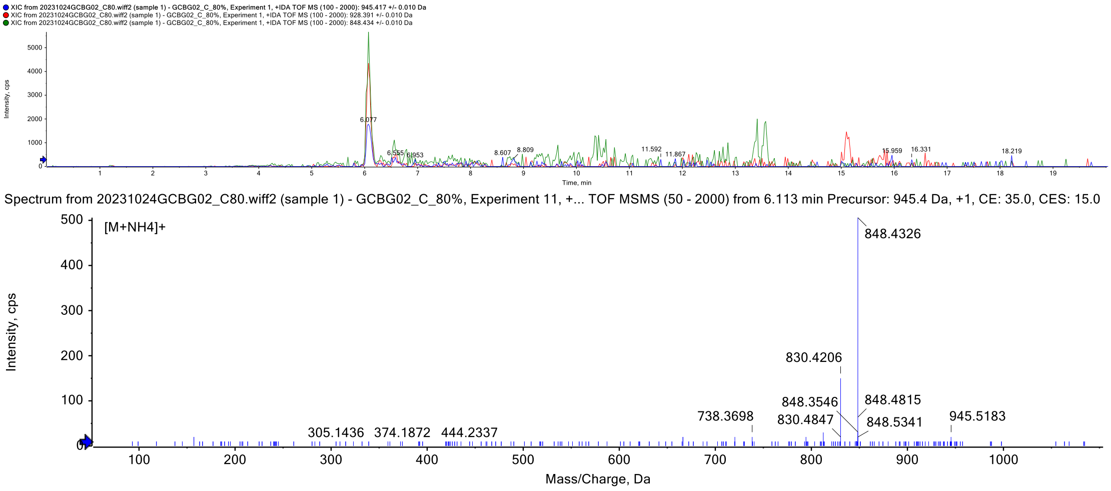

Spectrum from 20231024GCBG02_C80.wiff2 (sample 1) - GCBG02_C_80%, Experiment 10, +... TOF MSMS (50 - 2000) from 6.112 min Precursor: 928.4 Da, +1, CE: 35.0, CES: 15.0
With 2 other merged spectra

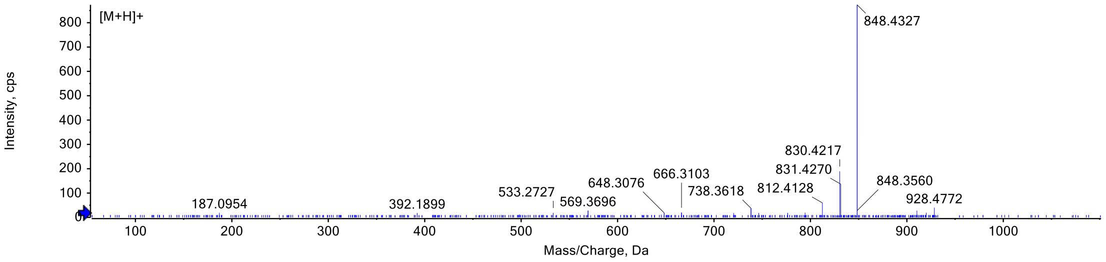

Spectrum from 20231024GCBG02_C80.wiff2 (sample 1) - GCBG02_C_80%, Experiment 10, +... TOF MSMS (50 - 2000) from 6.089 min Precursor: 848.4 Da, +1, CE: 35.0, CES: 15.0

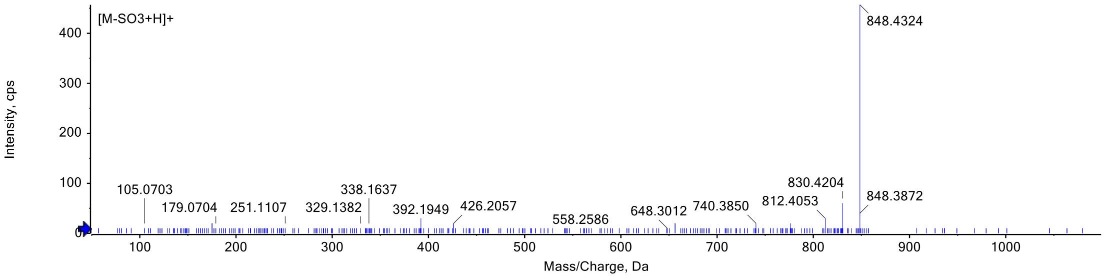

XIC from 20231024GCBG02_A80.wiff2 (sample 1) - GCBG02_A_80%, Experiment 1, +IDA TOF MS (100 - 2000): 1104.665 +/- 0.010 Da
XIC from 20231024GCBG02_A80.wiff2 (sample 1) - GCBG02_A_80%, Experiment 1, +IDA TOF MS (100 - 2000): 1122.678 +/- 0.010 Da

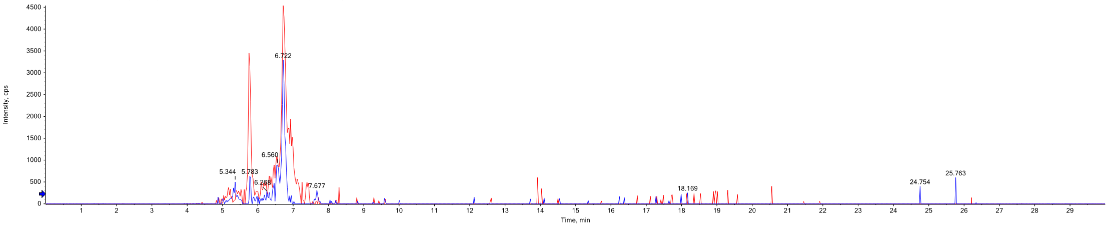

Spectrum from 20231024GCBG02_A80.wiff2 (sample 1) - GCBG02_A_80%, Experiment 11, +... TOF MSMS (50 - 2000) from 6.760 min Precursor: 1122.7 Da, +1, CE: 35.0, CES: 15.0

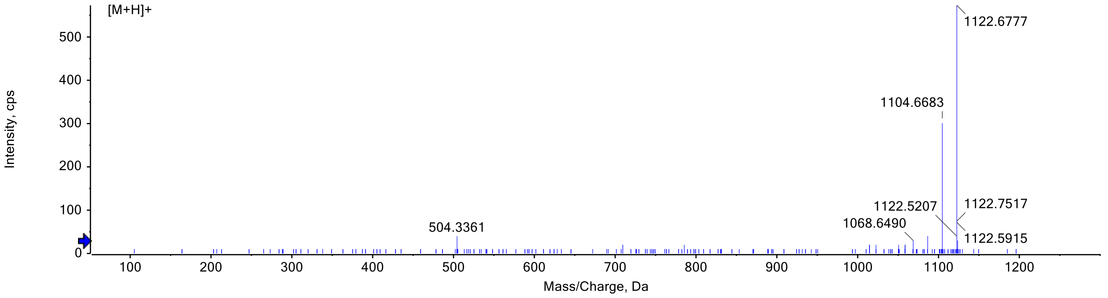

Spectrum from 20231024GCBG02_A80.wiff2 (sample 1) - GCBG02_A_80%, Experiment 10, +... TOF MSMS (50 - 2000) from 6.758 min Precursor: 1104.7 Da, +1, CE: 35.0, CES: 15.0

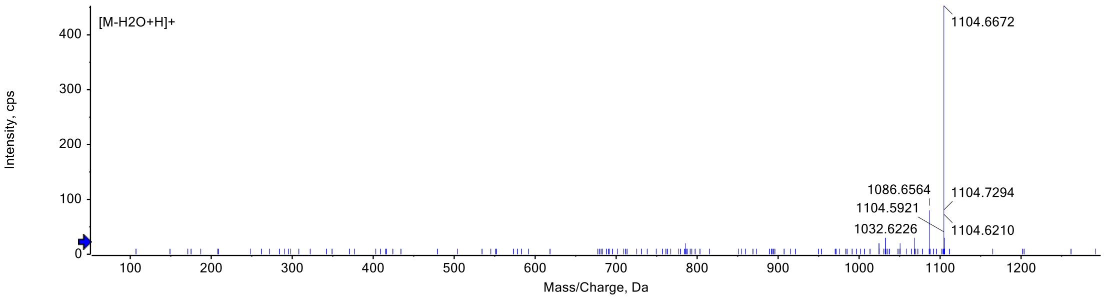

XIC from 20231024GCBG02_A80.wiff2 (sample 1) - GCBG02_A_80%, Experiment 1, +IDA TOF MS (100 - 2000): 913.426 +/- 0.010 Da
XIC from 20231024GCBG02_A80.wiff2 (sample 1) - GCBG02_A_80%, Experiment 1, +IDA TOF MS (100 - 2000): 896.400 +/- 0.010 Da
XIC from 20231024GCBG02_A80.wiff2 (sample 1) - GCBG02_A_80%, Experiment 1, +IDA TOF MS (100 - 2000): 816.442 +/- 0.010 Da

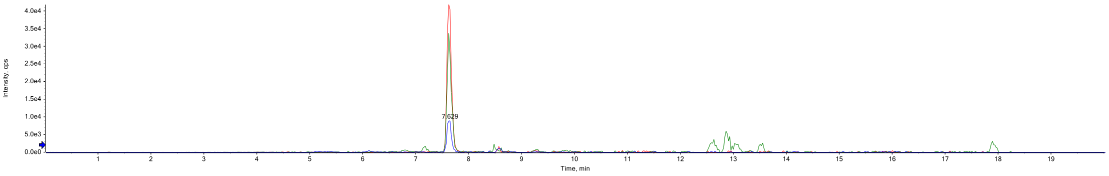

Spectrum from 20231024GCBG02_A80.wiff2 (sample 1) - GCBG02_A_80%, Experiment 11, +... TOF MSMS (50 - 2000) from 7.667 min Precursor: 913.4 Da, +1, CE: 35.0, CES: 15.0
With 1 other merged spectrum

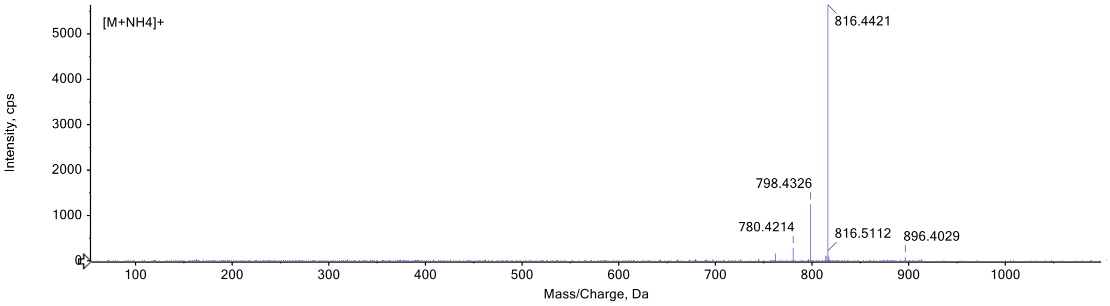

Spectrum from 20231024GCBG02_A80.wiff2 (sample 1) - GCBG02_A_80%, Experiment 11, +... TOF MSMS (50 - 2000) from 7.597 min Precursor: 896.4 Da, +1, CE: 35.0, CES: 15.0
With 3 other merged spectra

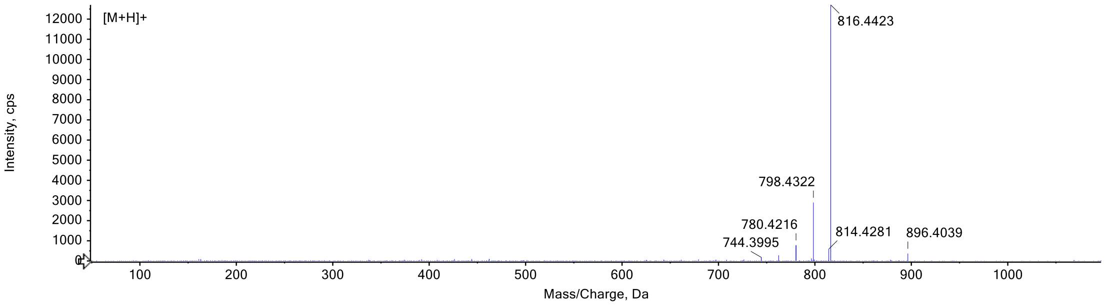

Spectrum from 20231024GCBG02_A80.wiff2 (sample 1) - GCBG02_A_80%, Experiment 7, +IDA TOF MSMS (50 - 2000) from 7.637 min Precursor: 816.4 Da, +1, CE: 35.0, CES: 15.0

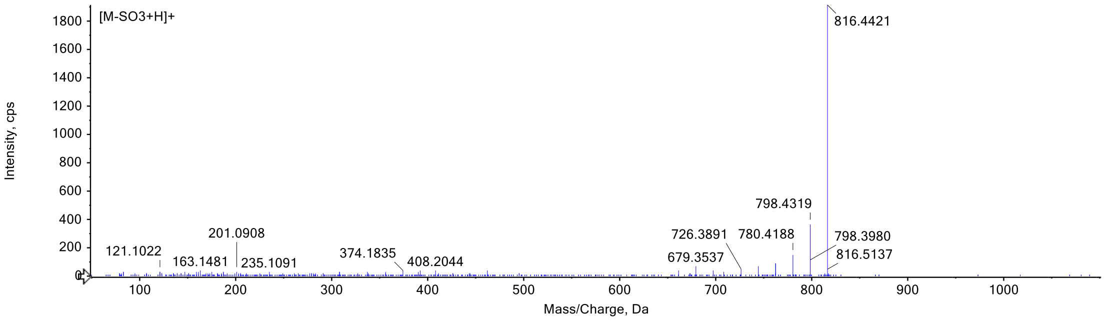

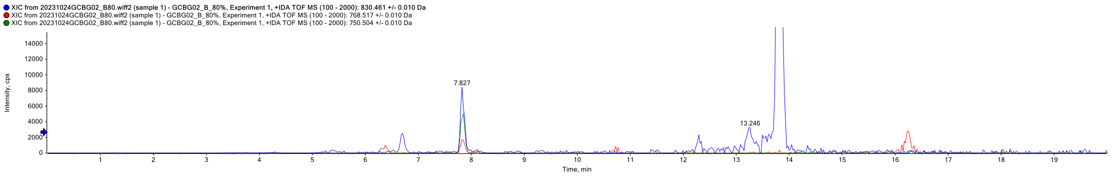

Spectrum from 20231024GCBG02_B80.wiff2 (sample 1) - GCBG02_B_80%, Experiment 9, +IDA TOF MSMS (50 - 2000) from 7.866 min Precursor: 830.5 Da, +1, CE: 35.0, CES: 15.0

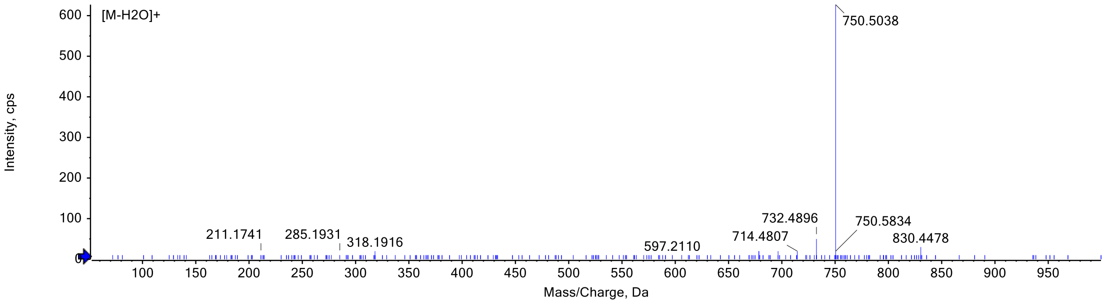

Spectrum from 20231024GCBG02_B80.wiff2 (sample 1) - GCBG02_B_80%, Experiment 8, +IDA TOF MSMS (50 - 2000) from 7.864 min Precursor: 768.5 Da, +1, CE: 35.0, CES: 15.0

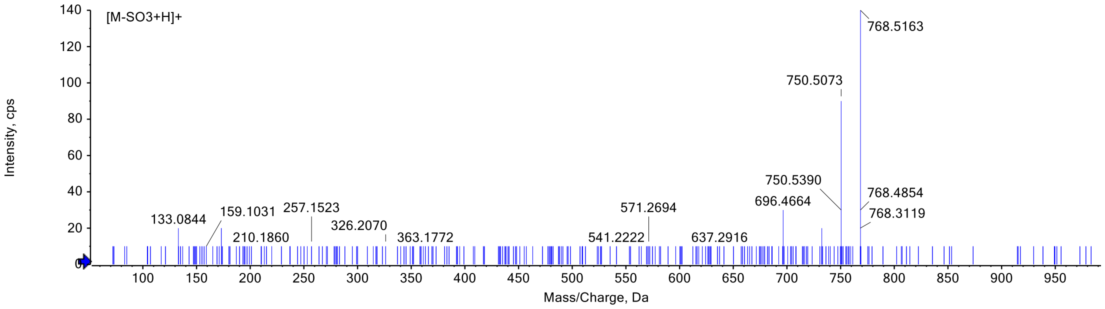

Spectrum from 20231024GCBG02_B80.wiff2 (sample 1) - GCBG02_B_80%, Experiment 7, +IDA TOF MSMS (50 - 2000) from 7.862 min Precursor: 750.5 Da, +1, CE: 35.0, CES: 15.0
With 3 other merged spectra

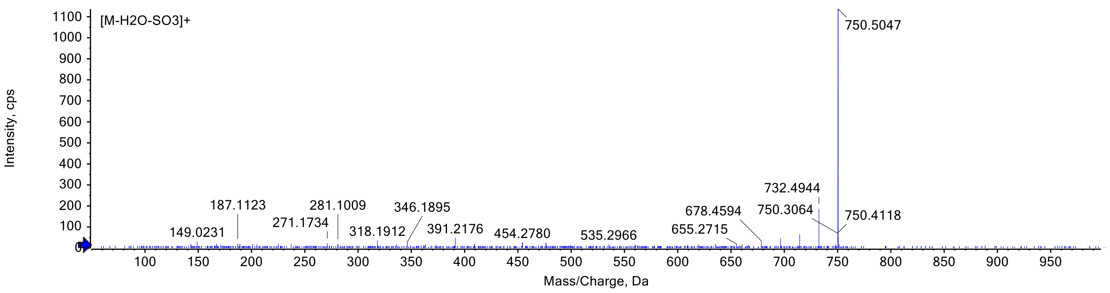

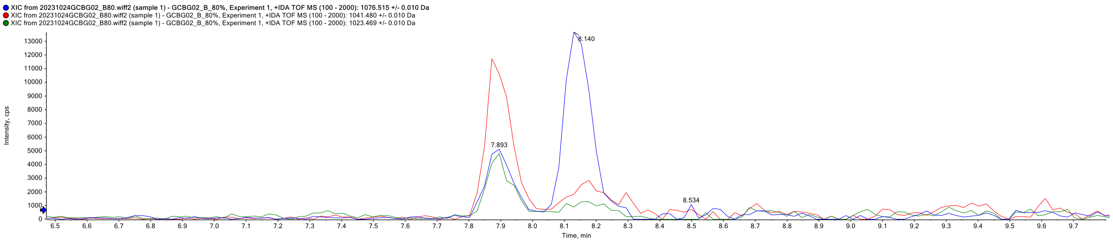

Spectrum from 20231024GCBG02_B80.wiff2 (sample 1) - GCBG02_B_80%, Experiment 11, +... TOF MSMS (50 - 2000) from 8.149 min Precursor: 1076.5 Da, +1, CE: 35.0, CES: 15.0

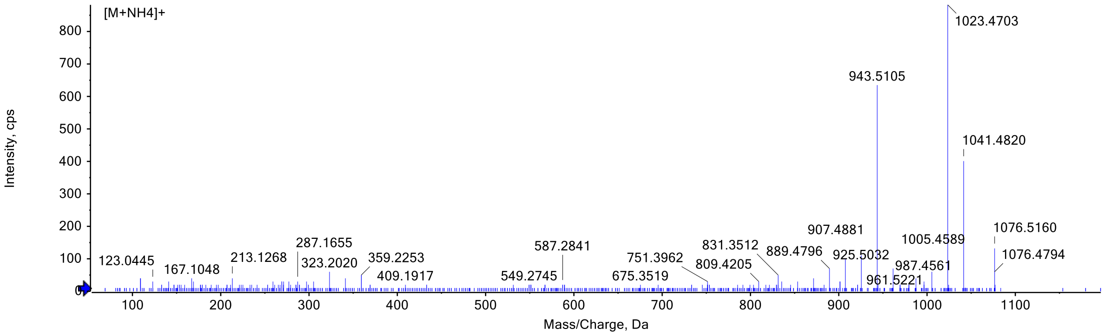

Spectrum from 20231024GCBG02_B80.wiff2 (sample 1) - GCBG02_B_80%, Experiment 11, +... TOF MSMS (50 - 2000) from 7.914 min Precursor: 1041.5 Da, +1, CE: 35.0, CES: 15.0

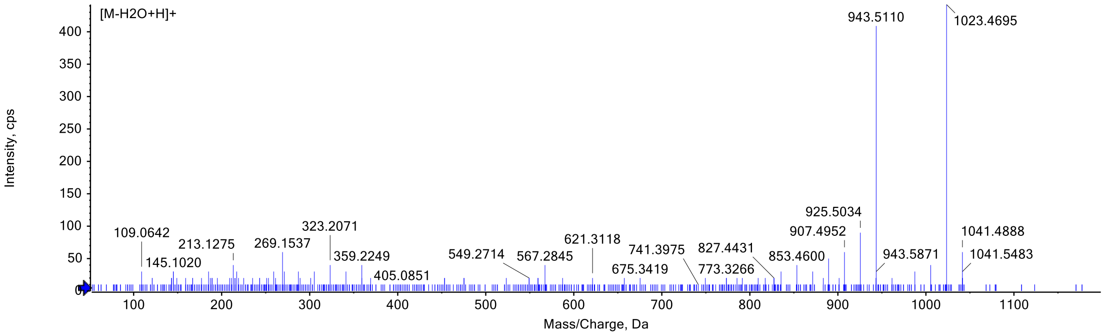

Spectrum from 20231024GCBG02_B80.wiff2 (sample 1) - GCBG02_B_80%, Experiment 10, +... TOF MSMS (50 - 2000) from 7.912 min Precursor: 1023.5 Da, +1, CE: 35.0, CES: 15.0

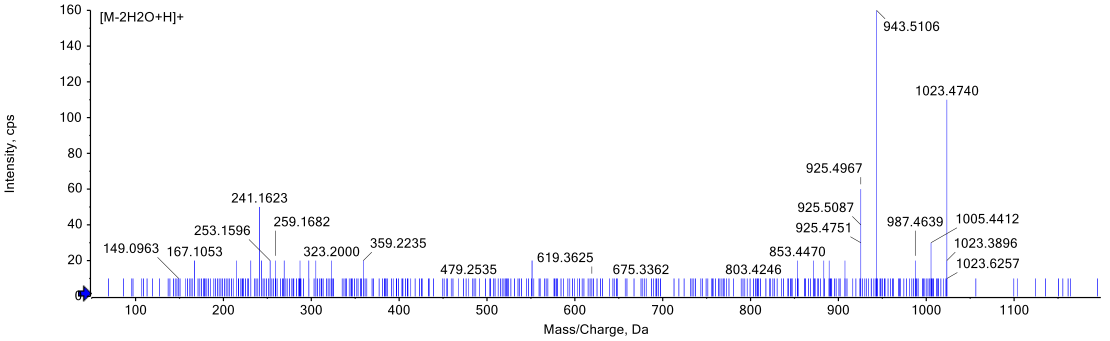

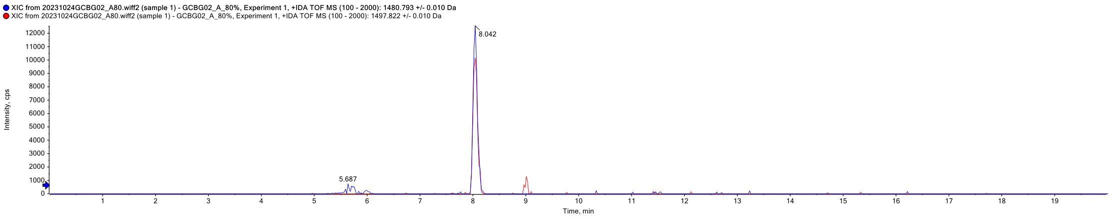

Spectrum from 20231024GCBG02_A80.wiff2 (sample 1) - GCBG02_A_80%, Experiment ...MSMS (50 - 2000) from 8.062 min Precursor: 1497.8 Da, +1, CE: 35.0, CES: 15.0
With 2 other merged spectra

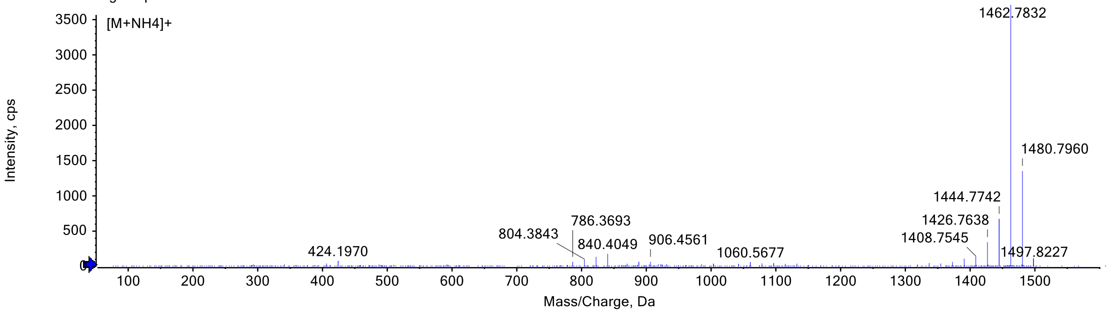

Spectrum from 20231024GCBG02_A80.wiff2 (sample 1) - GCBG02_A_80%, Experiment ...MSMS (50 - 2000) from 8.060 min Precursor: 1480.8 Da, +1, CE: 35.0, CES: 15.0
With 2 other merged spectra

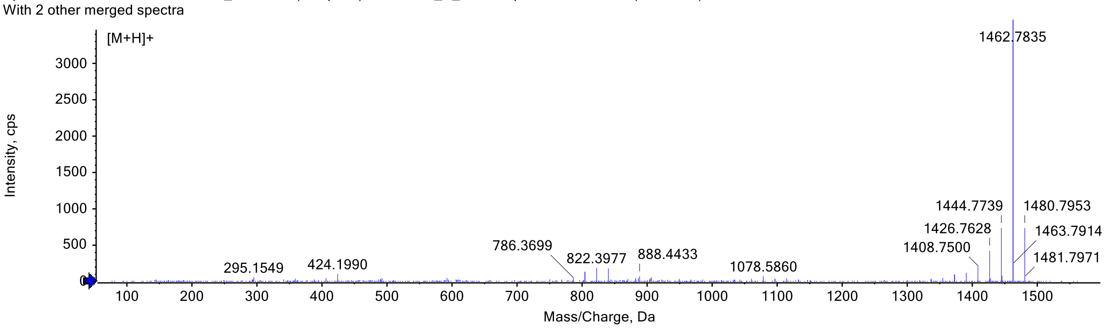

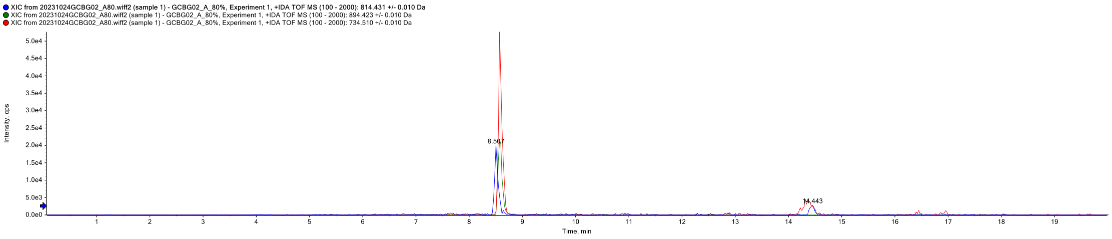

Spectrum from 20231024GCBG02_A80.wiff2 (sample 1) - GCBG02_A_80%, Experiment 11, +... TOF MSMS (50 - 2000) from 8.590 min Precursor: 894.4 Da, +1, CE: 35.0, CES: 15.0

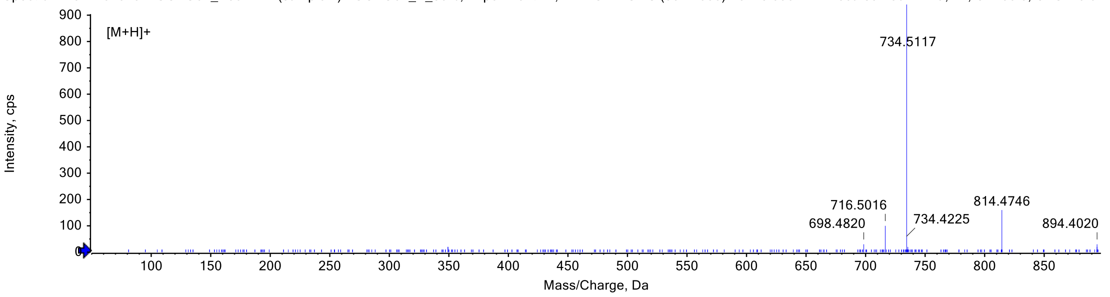

Spectrum from 20231024GCBG02_A80.wiff2 (sample 1) - GCBG02_A_80%, Experiment 10, +... TOF MSMS (50 - 2000) from 8.588 min Precursor: 814.5 Da, +1, CE: 35.0, CES: 15.0
With 1 other merged spectrum

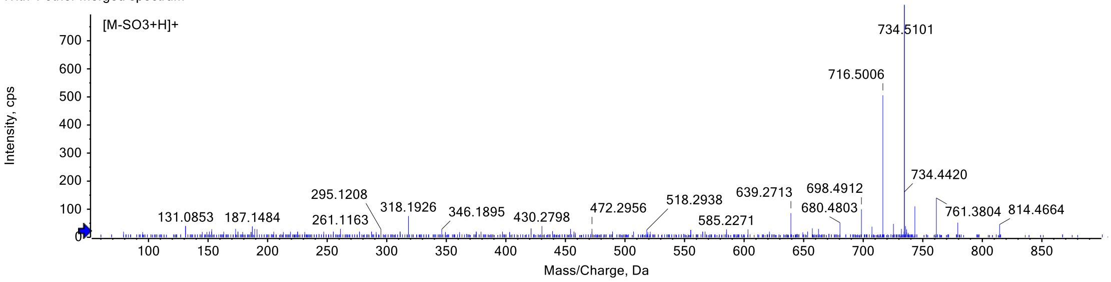

Spectrum from 20231024GCBG02_A80.wiff2 (sample 1) - GCBG02_A_80%, Experiment 9, +IDA TOF MSMS (50 - 2000) from 8.609 min Precursor: 734.5 Da, +1, CE: 35.0, CES: 15.0
With 1 other merged spectrum

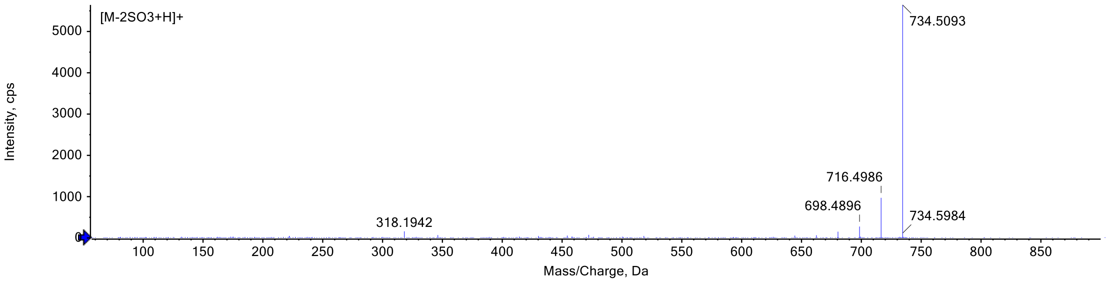

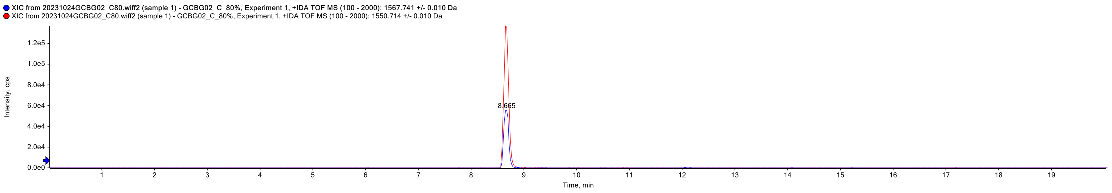

Spectrum from 20231024GCBG02_C80.wiff2 (sample 1) - GCBG02_C_80%, Experiment ...SMS (50 - 2000) from 8.715 min Precursor: 1567.7 Da, +2, CE: 35.0, CES: 15.0
With 4 other merged spectra

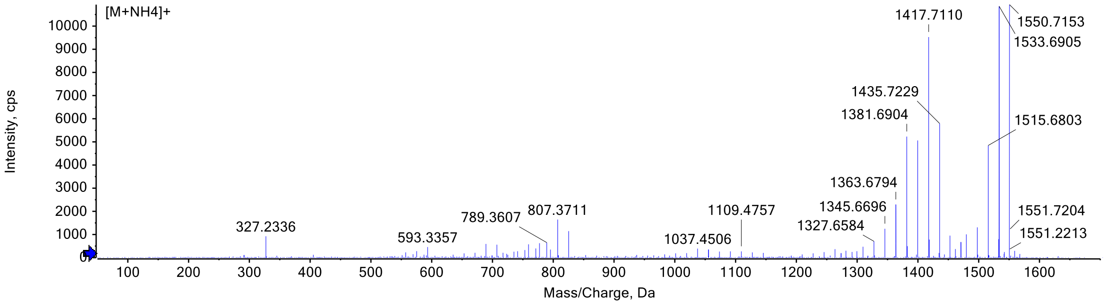

Spectrum from 20231024GCBG02_C80.wiff2 (sample 1) - GCBG02_C_80%, Experiment ...SMS (50 - 2000) from 8.690 min Precursor: 1550.7 Da, +1, CE: 35.0, CES: 15.0
With 6 other merged spectra

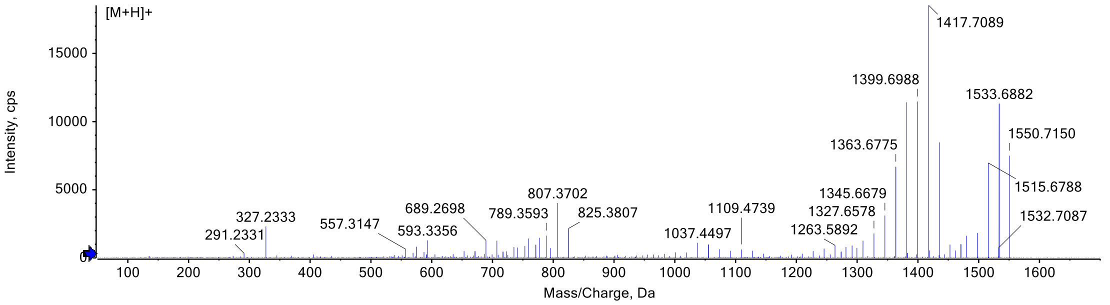

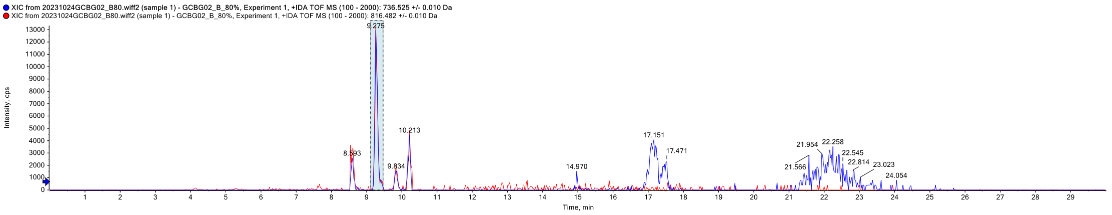

Spectrum from 20231024GCBG02_B80.wiff2 (sample 1) - GCBG02_B_80%, Experiment ... MSMS (50 - 2000) from 9.280 min Precursor: 816.5 Da, +1, CE: 35.0, CES: 15.0
With 1 other merged spectrum

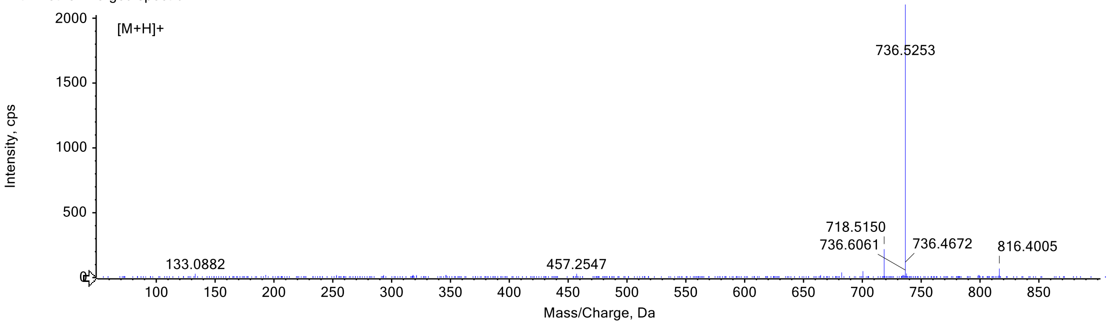

Spectrum from 20231024GCBG02_B80.wiff2 (sample 1) - GCBG02_B_80%, Experiment ... MSMS (50 - 2000) from 9.253 min Precursor: 736.5 Da, +1, CE: 35.0, CES: 15.0
With 1 other merged spectrum

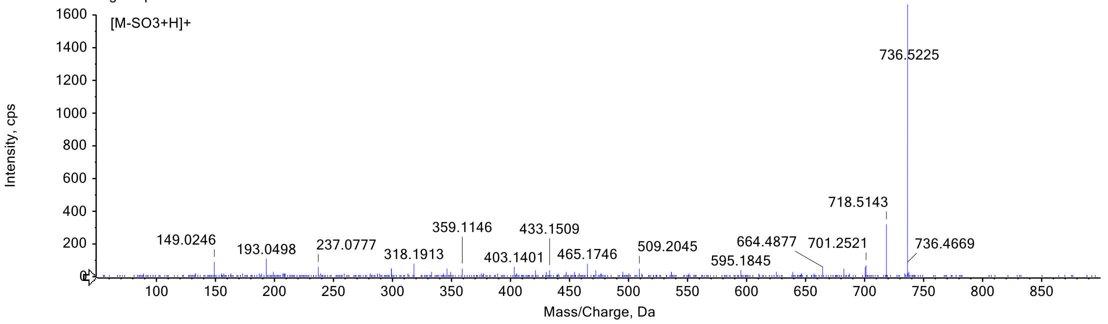

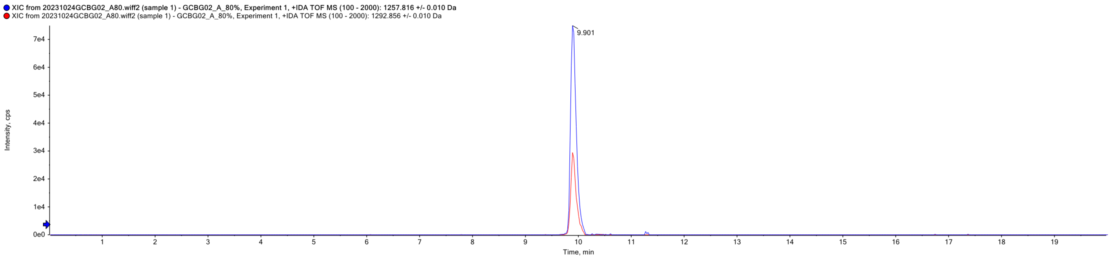

Spectrum from 20231024GCBG02_A80.wiff2 (sample 1) - GCBG02_A_80%, Experiment ...MSMS (50 - 2000) from 9.978 min Precursor: 1292.9 Da, +1, CE: 35.0, CES: 15.0
With 3 other merged spectra

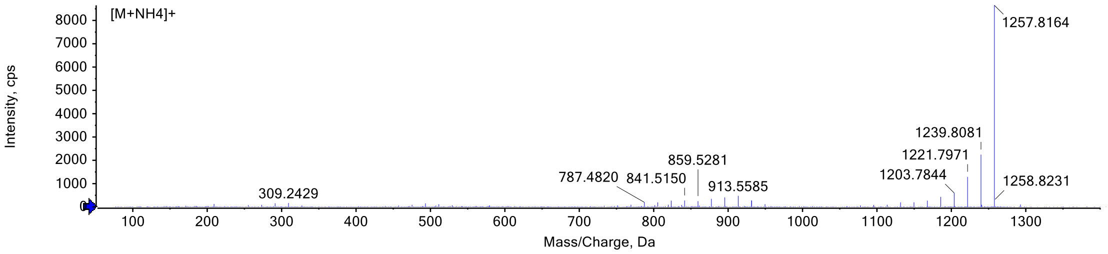

Spectrum from 20231024GCBG02_A80.wiff2 (sample 1) - GCBG02_A_80%, Experiment ...MSMS (50 - 2000) from 9.884 min Precursor: 1257.8 Da, +1, CE: 35.0, CES: 15.0
With 6 other merged spectra

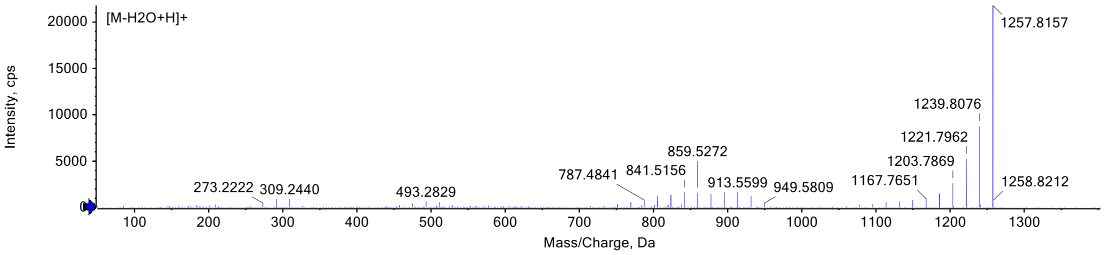

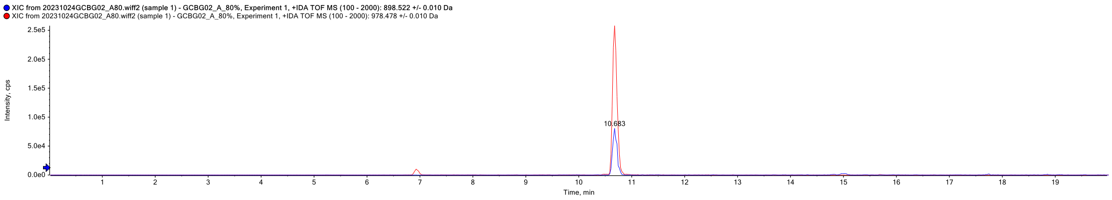

Spectrum from 20231024GCBG02_A80.wiff2 (sample 1) - GCBG02_A_80%, Experiment ...MSMS (50 - 2000) from 10.718 min Precursor: 978.5 Da, +1, CE: 35.0, CES: 15.0
With 4 other merged spectra

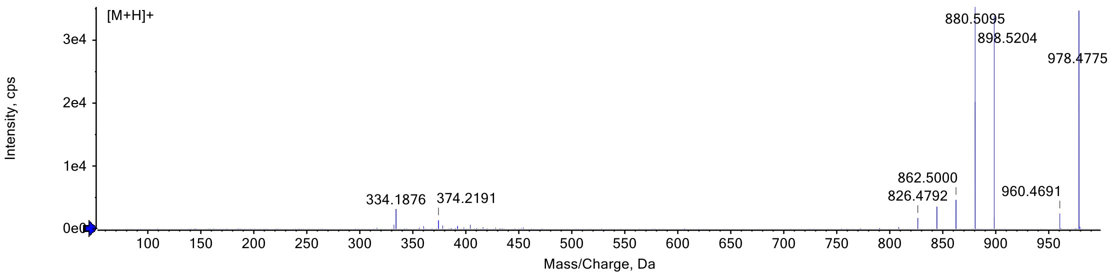

Spectrum from 20231024GCBG02_A80.wiff2 (sample 1) - GCBG02_A_80%, Experiment ...MSMS (50 - 2000) from 10.739 min Precursor: 898.5 Da, +1, CE: 35.0, CES: 15.0

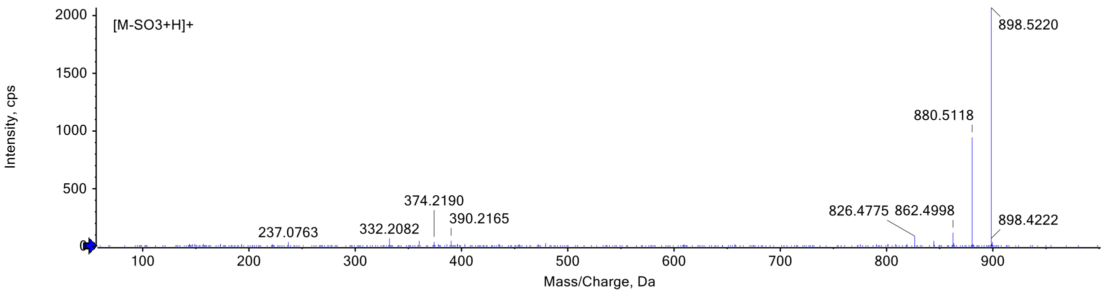

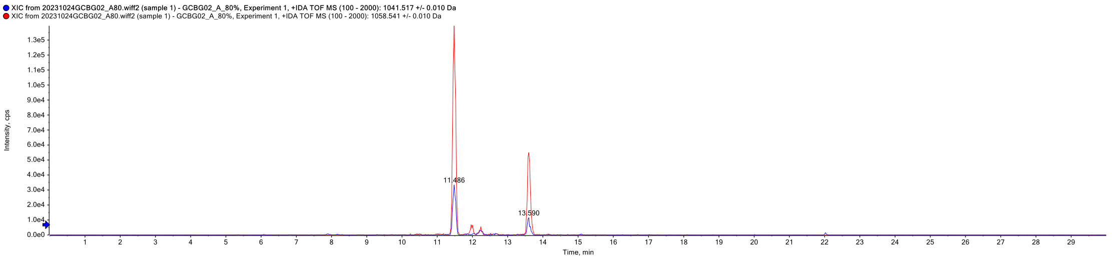

Spectrum from 20231024GCBG02_A80.wiff2 (sample 1) - GCBG02_A_80%, Experiment 11, +...OF MSMS (50 - 2000) from 11.498 min Precursor: 1058.5 Da, +1, CE: 35.0, CES: 15.0
With 4 other merged spectra

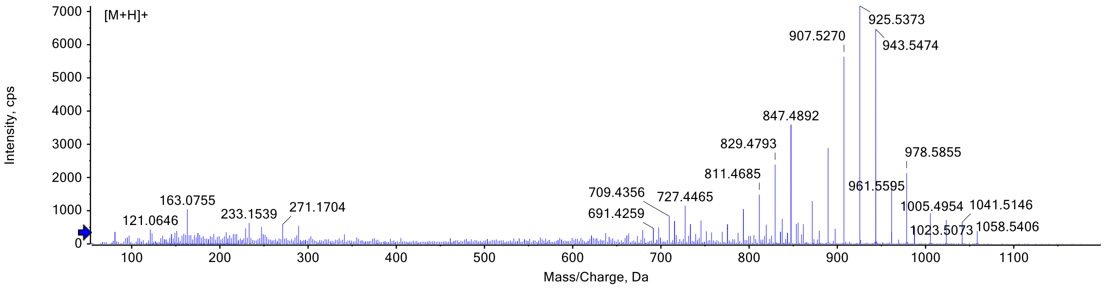

Spectrum from 20231024GCBG02_A80.wiff2 (sample 1) - GCBG02_A_80%, Experiment 9, +I...TOF MSMS (50 - 2000) from 11.447 min Precursor: 1041.5 Da, +1, CE: 35.0, CES: 15.0

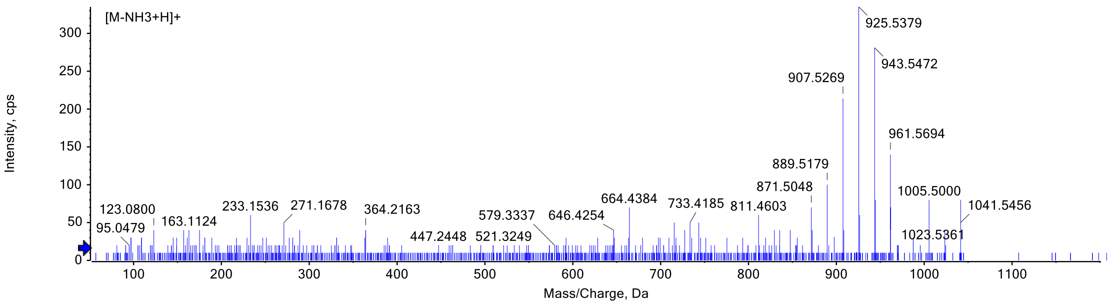

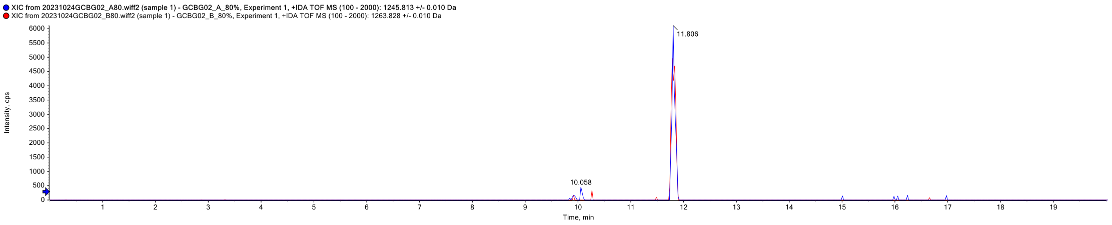

Spectrum from 20231024GCBG02_B80.wiff2 (sample 1) - GCBG02_B_80%, Experiment ...SMS (50 - 2000) from 11.803 min Precursor: 1263.8 Da, +1, CE: 35.0, CES: 15.0

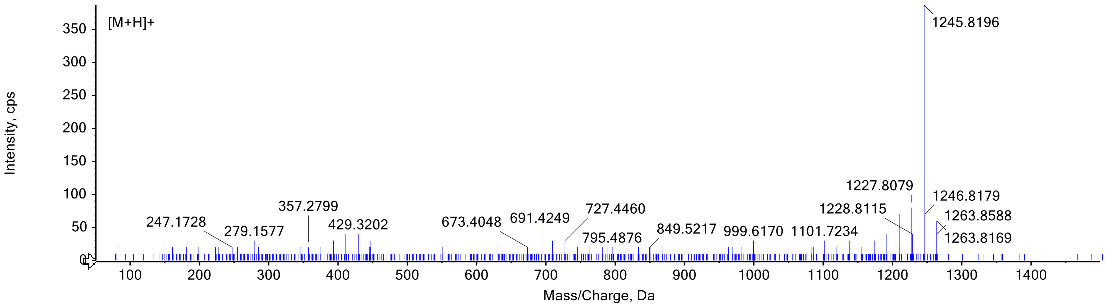

Spectrum from 20231024GCBG02_A80.wiff2 (sample 1) - GCBG02_A_80%, Experiment ...SMS (50 - 2000) from 11.820 min Precursor: 1245.8 Da, +1, CE: 35.0, CES: 15.0

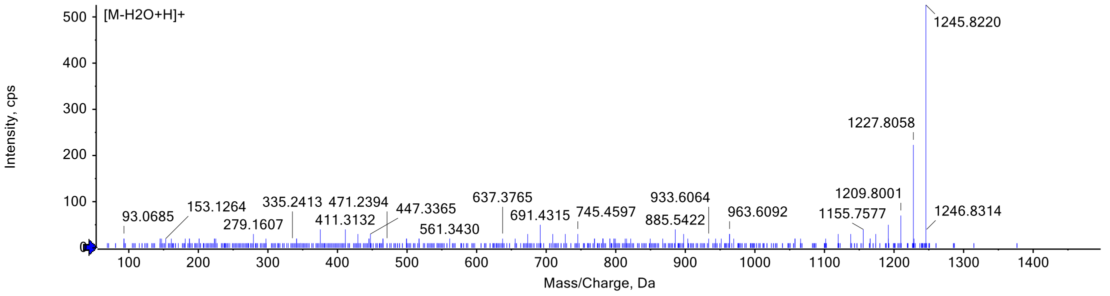

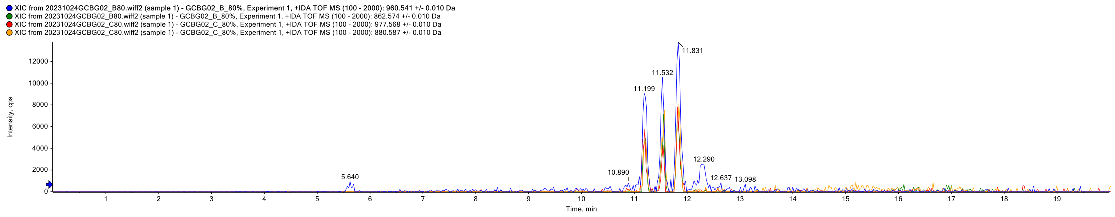

Spectrum from 20231024GCBG02_C80.wiff2 (sample 1) - GCBG02_C_80%, Experiment ...SMS (50 - 2000) from 11.840 min Precursor: 977.6 Da, +1, CE: 35.0, CES: 15.0

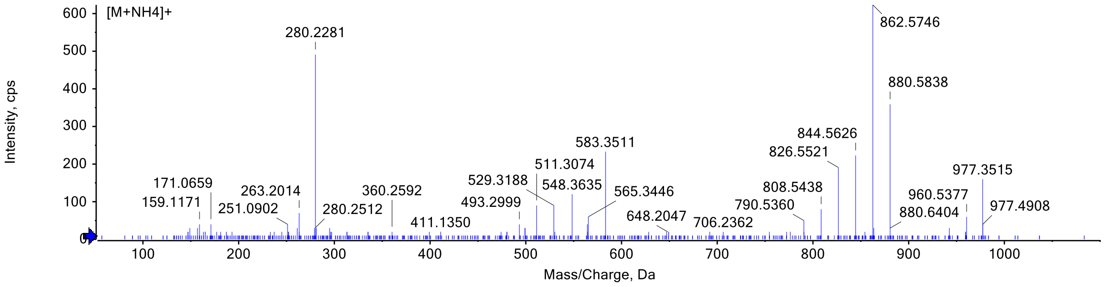

Spectrum from 20231024GCBG02_B80.wiff2 (sample 1) - GCBG02_B_80%, Experiment ...MSMS (50 - 2000) from 11.846 min Precursor: 960.5 Da, +1, CE: 35.0, CES: 15.0

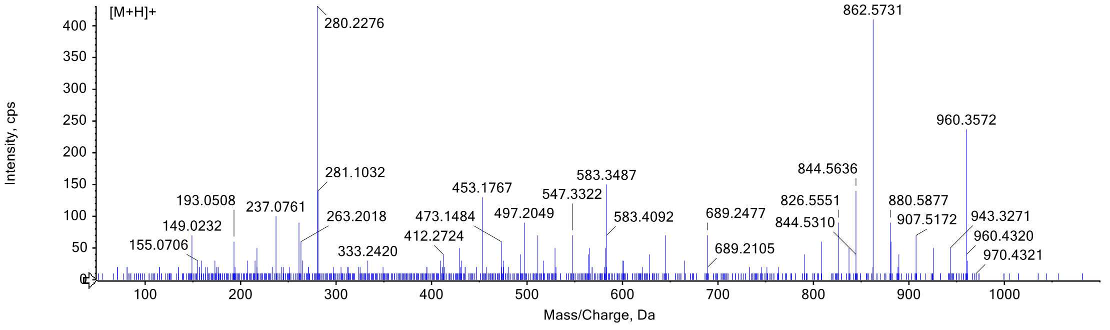

Spectrum from 20231024GCBG02_C80.wiff2 (sample 1) - GCBG02_C_80%, Experiment ...SMS (50 - 2000) from 11.838 min Precursor: 880.6 Da, +1, CE: 35.0, CES: 15.0

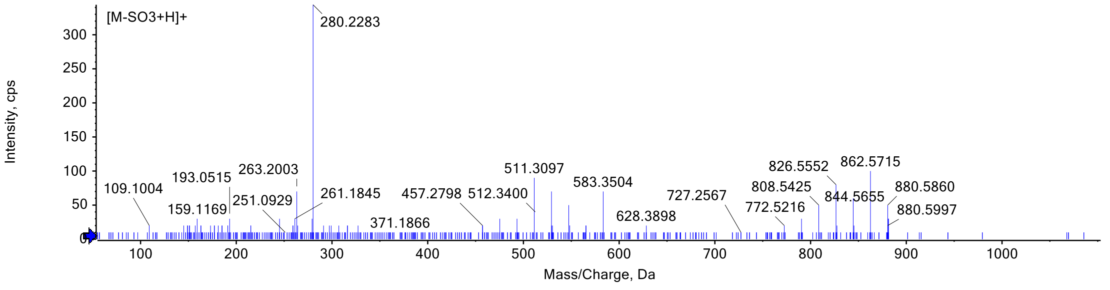

Spectrum from 20231024GCBG02_B80.wiff2 (sample 1) - GCBG02_B_80%, Experiment ...MSMS (50 - 2000) from 11.844 min Precursor: 862.6 Da, +1, CE: 35.0, CES: 15.0

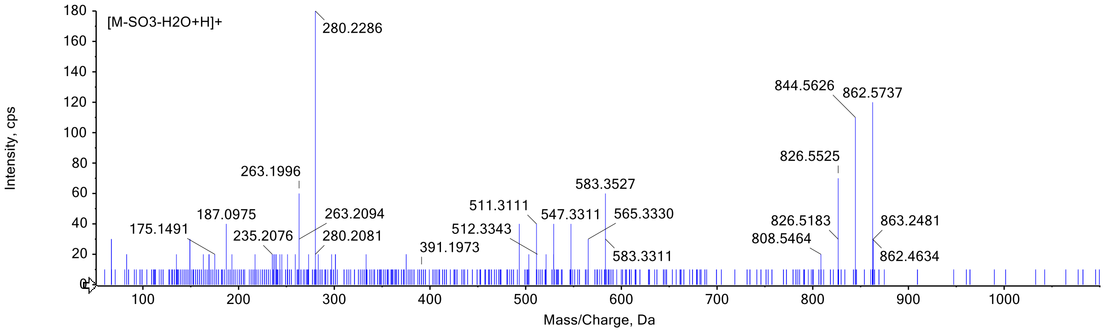

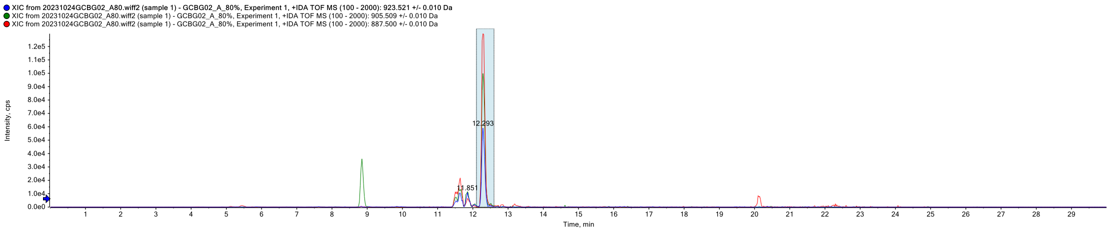

Spectrum from 20231024GCBG02_A80.wiff2 (sample 1) - GCBG02_A_80%, Experiment ...MSMS (50 - 2000) from 12.304 min Precursor: 923.5 Da, +1, CE: 35.0, CES: 15.0

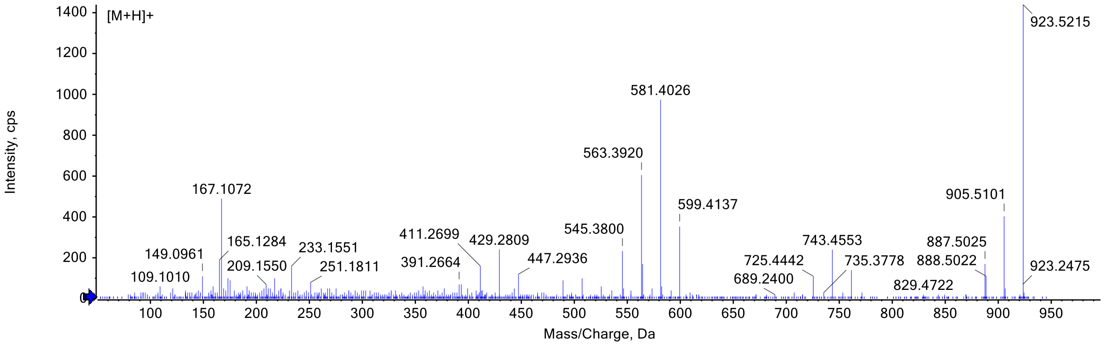

Spectrum from 20231024GCBG02_A80.wiff2 (sample 1) - GCBG02_A_80%, Experiment ...MSMS (50 - 2000) from 11.841 min Precursor: 905.5 Da, +1, CE: 35.0, CES: 15.0
With 1 other merged spectrum

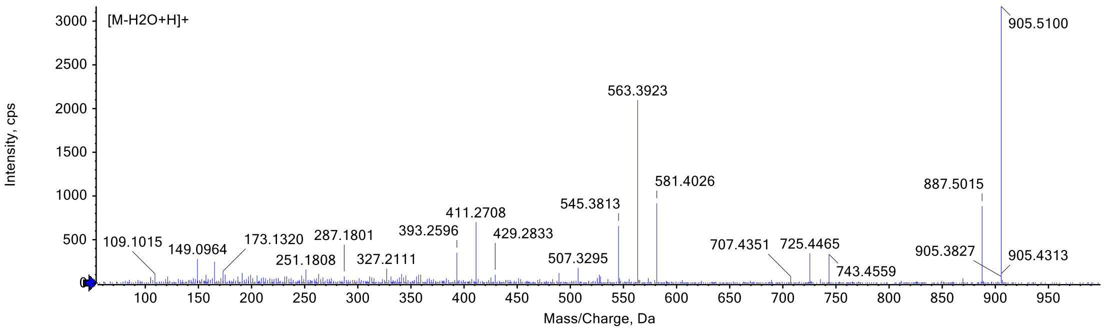

Spectrum from 20231024GCBG02_A80.wiff2 (sample 1) - GCBG02_A_80%, Experiment ...MSMS (50 - 2000) from 12.300 min Precursor: 887.5 Da, +1, CE: 35.0, CES: 15.0

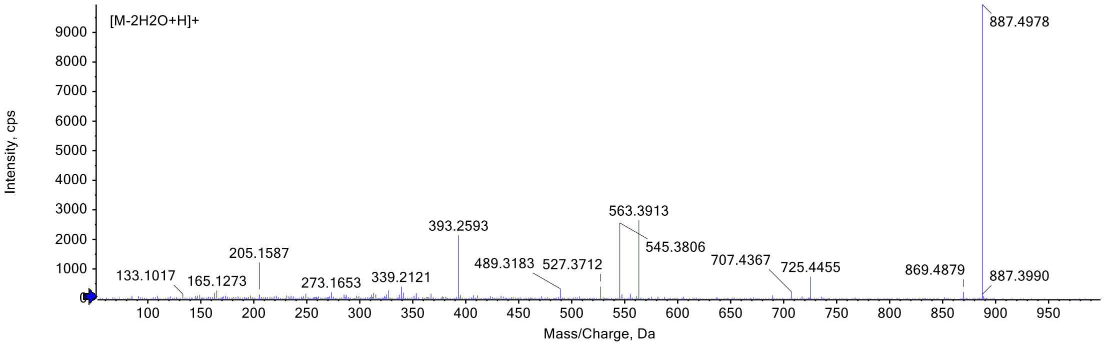

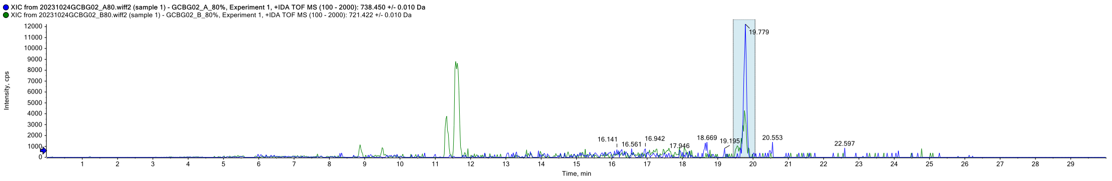

Spectrum from 20231024GCBG02_A80.wiff2 (sample 1) - GCBG02_A_80%, Experiment 7, +I... TOF MSMS (50 - 2000) from 19.777 min Precursor: 738.4 Da, +1, CE: 35.0, CES: 15.0
With 4 other merged spectra

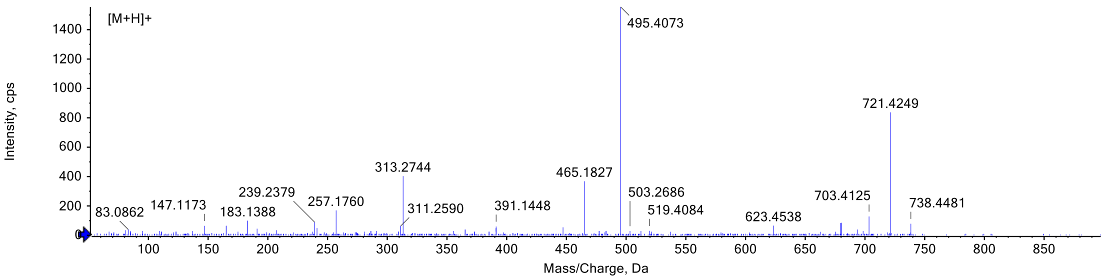

Spectrum from 20231024GCBG02_B80.wiff2 (sample 1) - GCBG02_B_80%, Experiment 4, +I... TOF MSMS (50 - 2000) from 19.788 min Precursor: 721.4 Da, +1, CE: 35.0, CES: 15.0

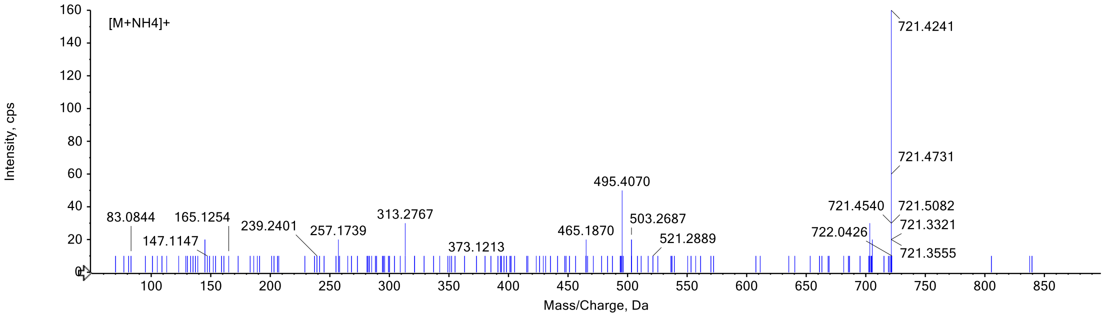

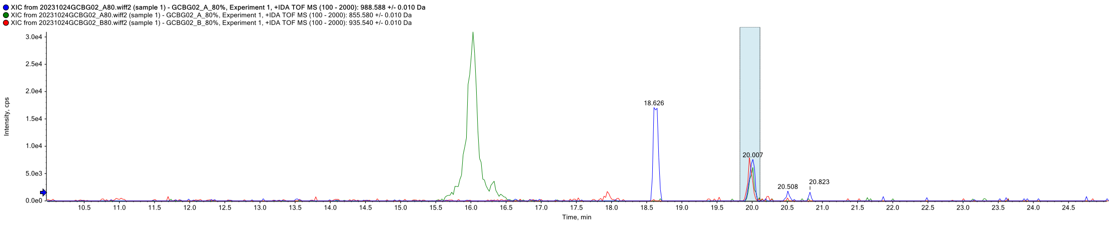

Spectrum from 20231024GCBG02_A80.wiff2 (sample 1) - GCBG02_A_80%, Experiment 9, +I... TOF MSMS (50 - 2000) from 20.074 min Precursor: 988.6 Da, +1, CE: 35.0, CES: 15.0
With 2 other merged spectra

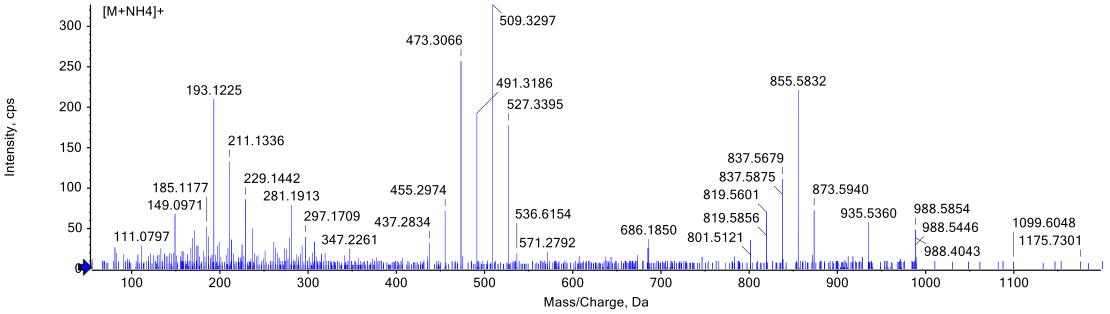

Spectrum from 20231024GCBG02_B80.wiff2 (sample 1) - GCBG02_B_80%, Experiment 10, +... TOF MSMS (50 - 2000) from 20.022 min Precursor: 935.5 Da, +1, CE: 35.0, CES: 15.0
With 2 other merged spectra

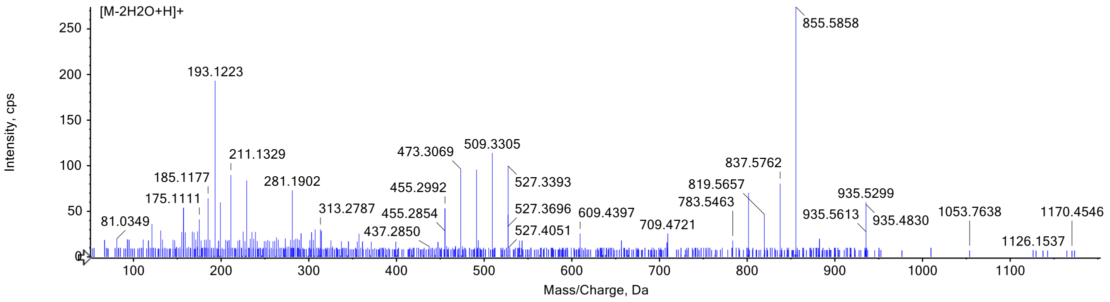

Spectrum from 20231024GCBG02_A80.wiff2 (sample 1) - GCBG02_A_80%, Experiment 7, +I... TOF MSMS (50 - 2000) from 19.978 min Precursor: 855.6 Da, +1, CE: 35.0, CES: 15.0
With 1 other merged spectrum

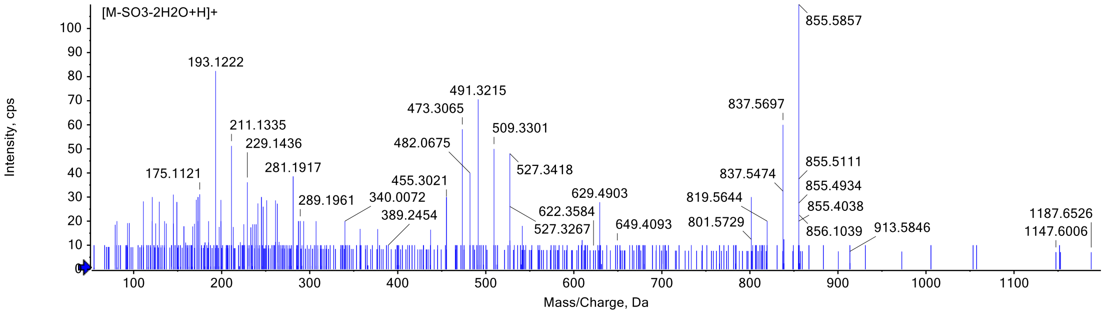

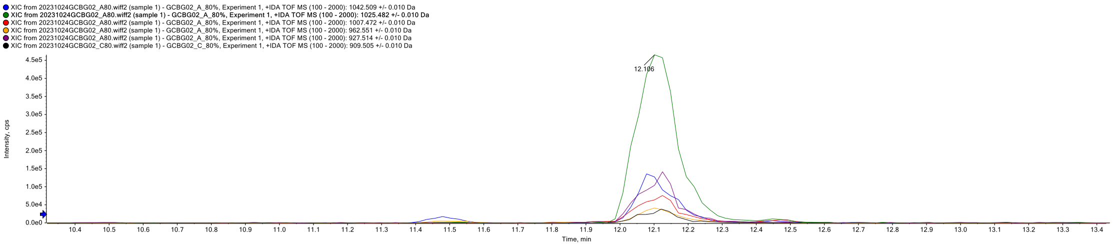

Spectrum from 20231024GCBG02_A80.wiff2 (sample 1) - GCBG02_A_80%, Experiment 10, +...OF MSMS (50 - 2000) from 12.095 min Precursor: 1042.5 Da, +1, CE: 35.0, CES: 15.0
With 7 other merged spectra

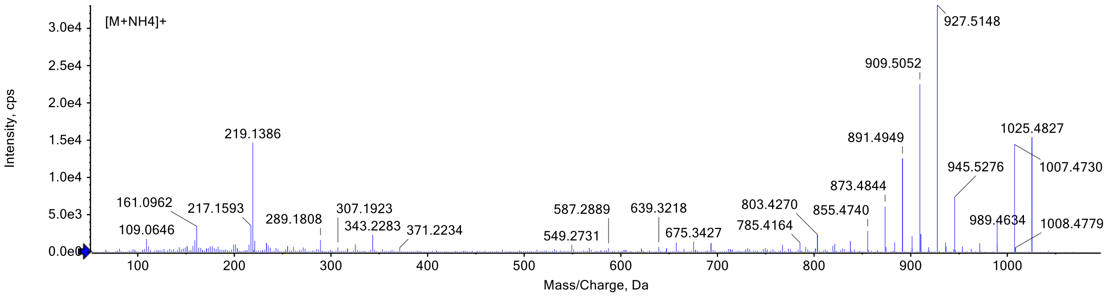

Spectrum from 20231024GCBG02_A80.wiff2 (sample 1) - GCBG02_A_80%, Experiment 9, +I...TOF MSMS (50 - 2000) from 12.140 min Precursor: 1025.5 Da, +1, CE: 35.0, CES: 15.0
With 8 other merged spectra

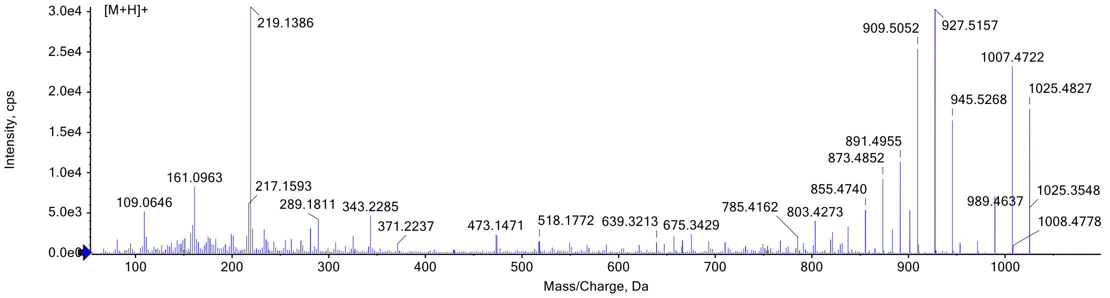

Spectrum from 20231024GCBG02_A80.wiff2 (sample 1) - GCBG02_A_80%, Experiment 9, +I...TOF MSMS (50 - 2000) from 12.047 min Precursor: 1007.5 Da, +1, CE: 35.0, CES: 15.0
With 5 other merged spectra

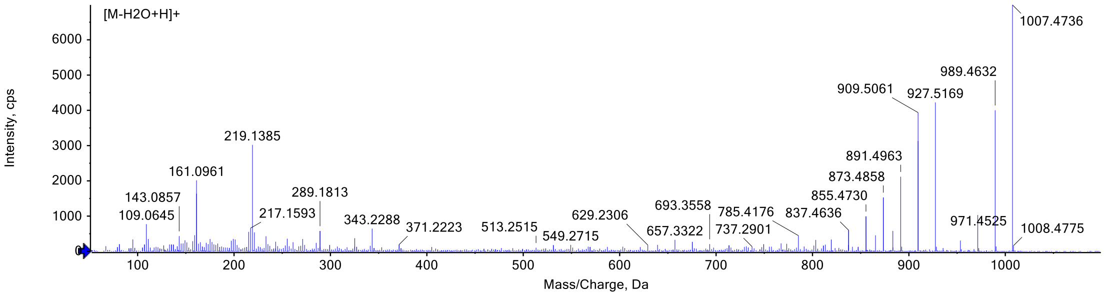

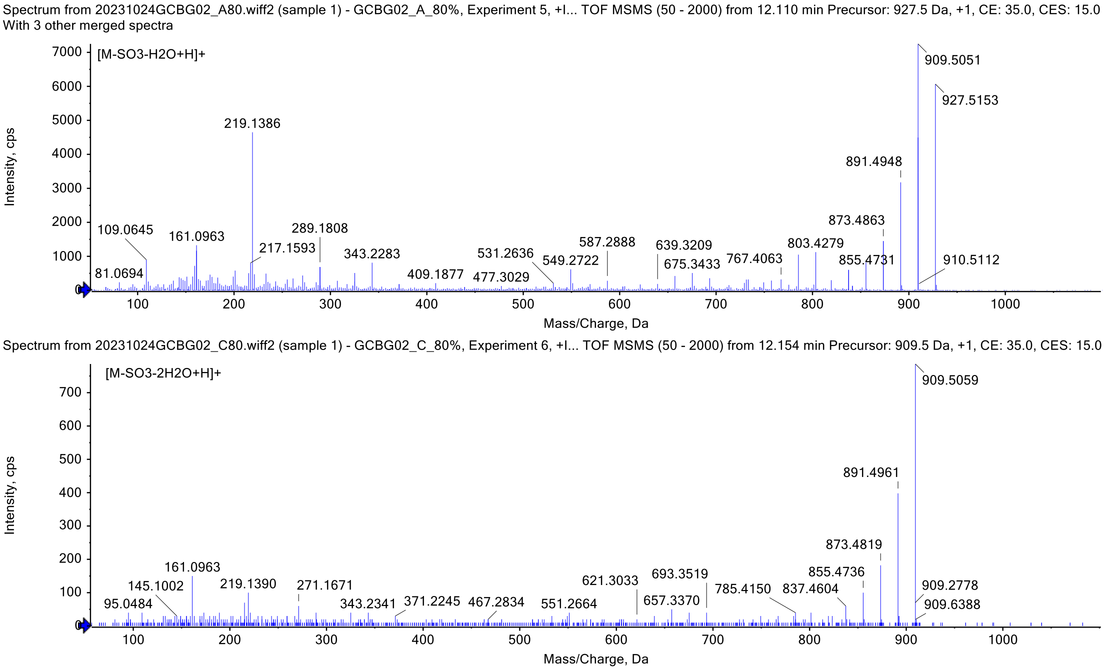

Figure S6. The MS^1 and MS^2 spectra of aligned adducts in *Gambierdiscus caribaeus* GCBG02

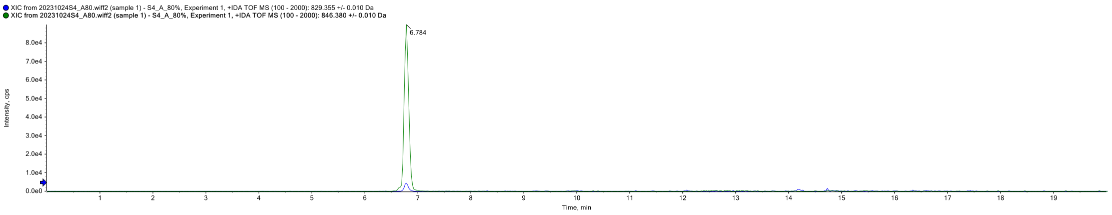

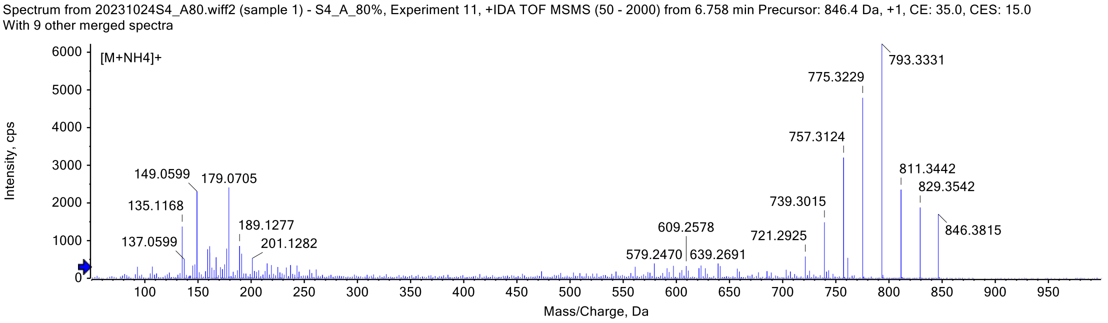

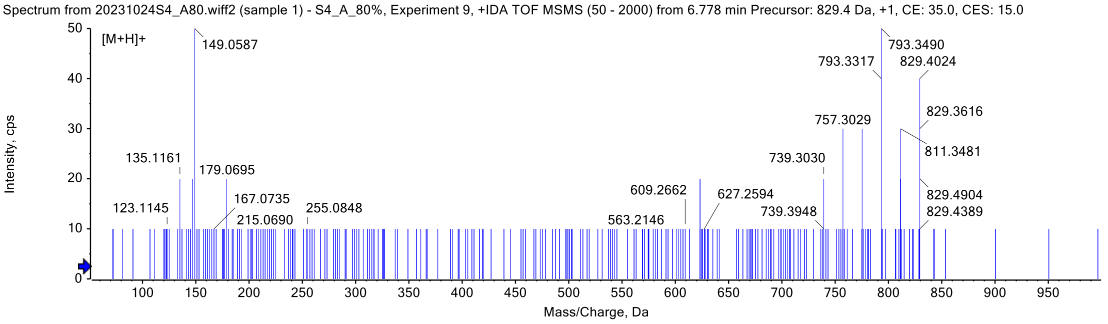

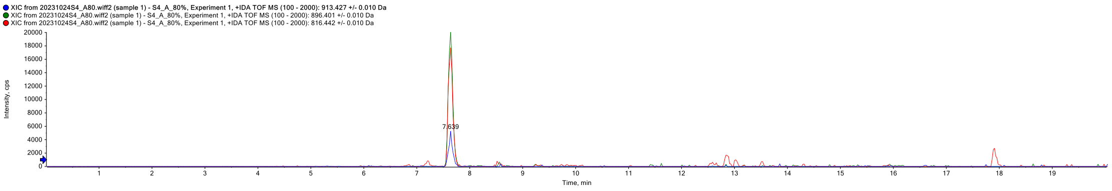

Spectrum from 20231024S4_A80.wiff2 (sample 1) - S4_A_80%, Experiment 11, +IDA TOF MSMS (50 - 2000) from 7.658 min Precursor: 913.4 Da, +1, CE: 35.0, CES: 15.0
With 1 other merged spectrum

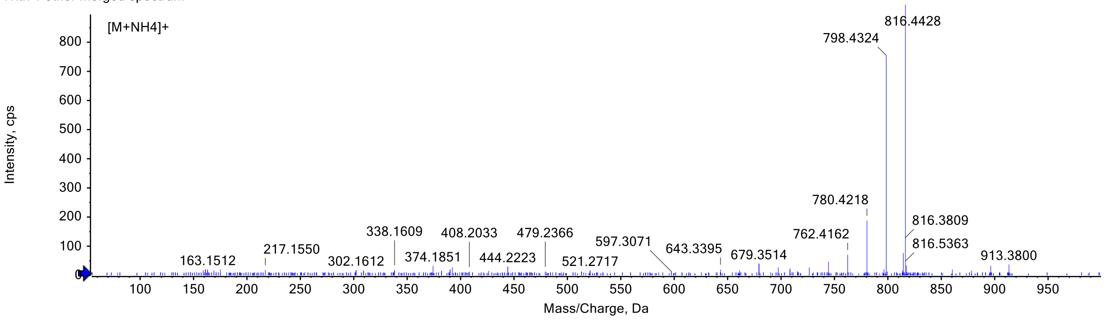

Spectrum from 20231024S4_A80.wiff2 (sample 1) - S4_A_80%, Experiment 11, +IDA TOF MSMS (50 - 2000) from 7.681 min Precursor: 896.4 Da, +1, CE: 35.0, CES: 15.0
With 5 other merged spectra

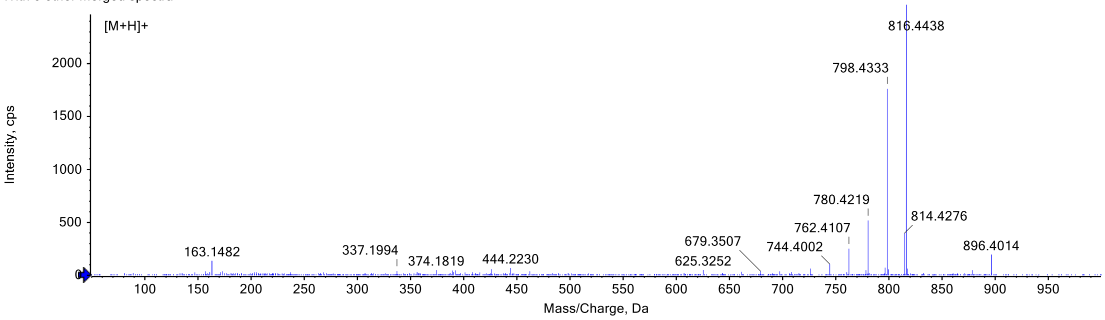

Spectrum from 20231024S4_A80.wiff2 (sample 1) - S4_A_80%, Experiment 10, +IDA TOF MSMS (50 - 2000) from 7.679 min Precursor: 816.4 Da, +1, CE: 35.0, CES: 15.0
With 2 other merged spectra

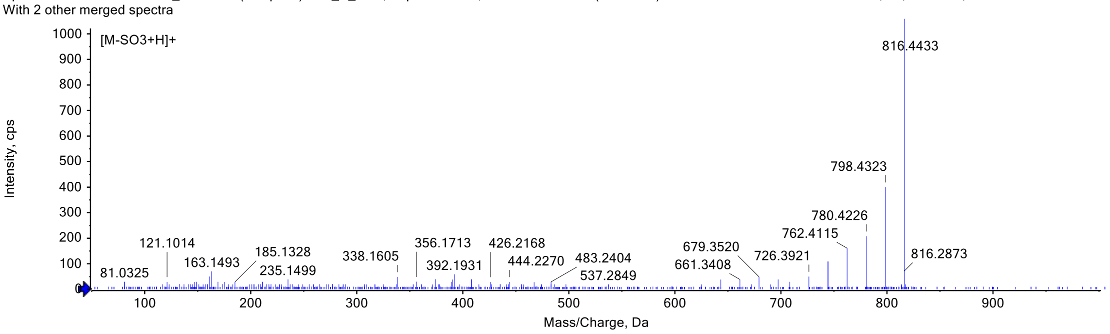

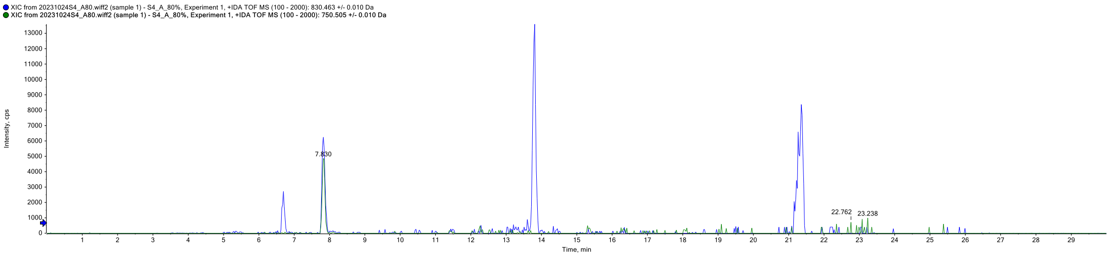

Spectrum from 20231024S4_A80.wiff2 (sample 1) - S4_A_80%, Experiment 11, +IDA TOF MSMS (50 - 2000) from 7.797 min Precursor: 830.5 Da, +1, CE: 35.0, CES: 15.0
With 3 other merged spectra

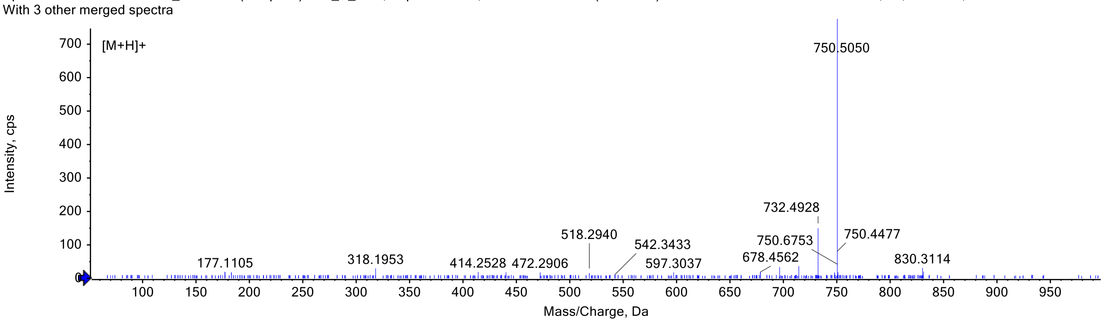

Spectrum from 20231024S4_A80.wiff2 (sample 1) - S4_A_80%, Experiment 10, +IDA TOF MSMS (50 - 2000) from 7.864 min Precursor: 750.5 Da, +1, CE: 35.0, CES: 15.0
With 1 other merged spectrum

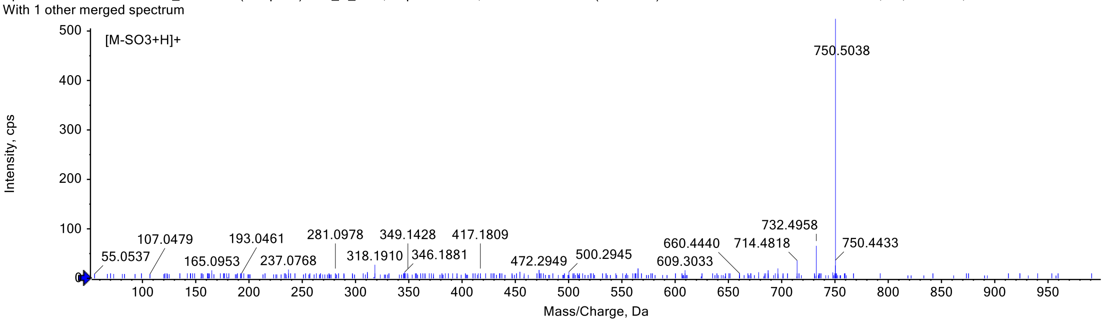

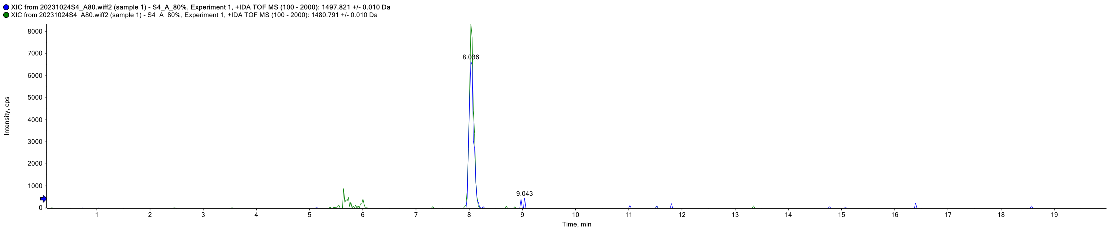

Spectrum from 20231024S4_A80.wiff2 (sample 1) - S4_A_80%, Experiment 11, +IDA TOF MSMS (50 - 2000) from 8.027 min Precursor: 1497.8 Da, +1, CE: 35.0, CES: 15.0
With 1 other merged spectrum

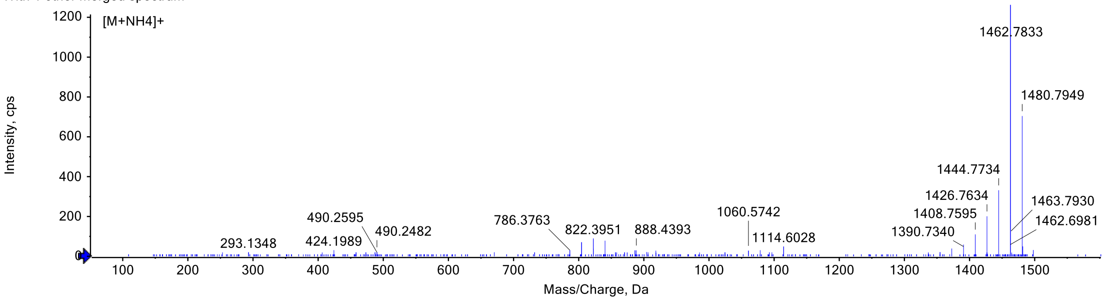

Spectrum from 20231024S4_A80.wiff2 (sample 1) - S4_A_80%, Experiment 11, +IDA TOF MSMS (50 - 2000) from 8.050 min Precursor: 1480.8 Da, +1, CE: 35.0, CES: 15.0
With 3 other merged spectra

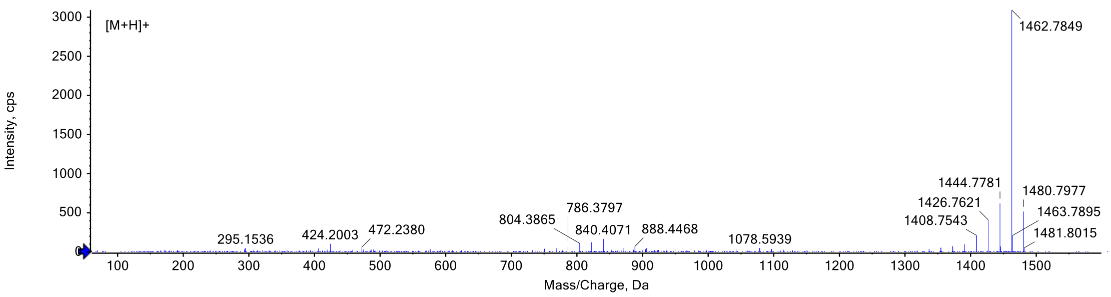

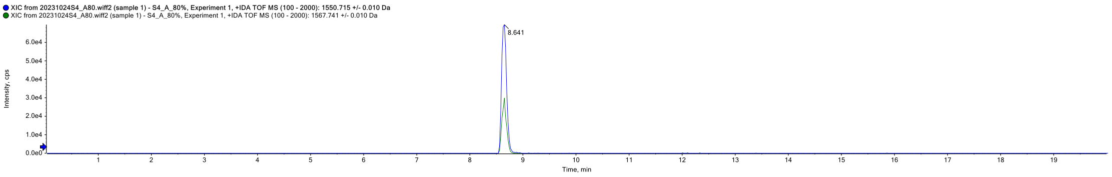

Spectrum from 20231024S4_A80.wiff2 (sample 1) - S4_A_80%, Experiment 10, +IDA TOF MSMS (50 - 2000) from 8.667 min Precursor: 1567.7 Da, +2, CE: 35.0, CES: 15.0
With 2 other merged spectra

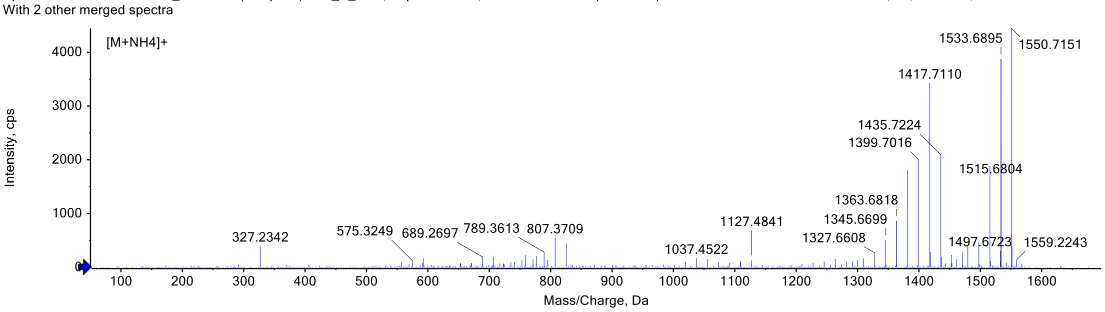

Spectrum from 20231024S4_A80.wiff2 (sample 1) - S4_A_80%, Experiment 11, +IDA TOF MSMS (50 - 2000) from 8.738 min Precursor: 1550.7 Da, +1, CE: 35.0, CES: 15.0
With 6 other merged spectra

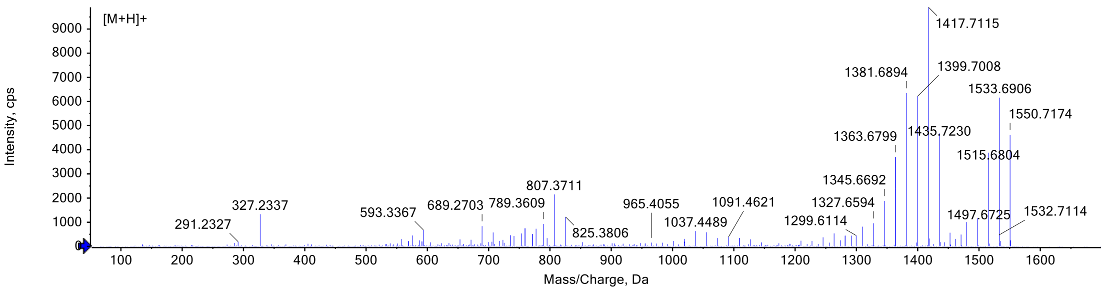

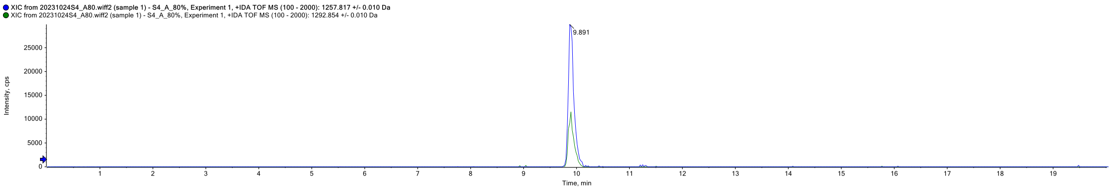

Spectrum from 20231024S4_A80.wiff2 (sample 1) - S4_A_80%, Experiment 11, +IDA TOF MSMS (50 - 2000) from 9.867 min Precursor: 1292.9 Da, +1, CE: 35.0, CES: 15.0
With 1 other merged spectrum

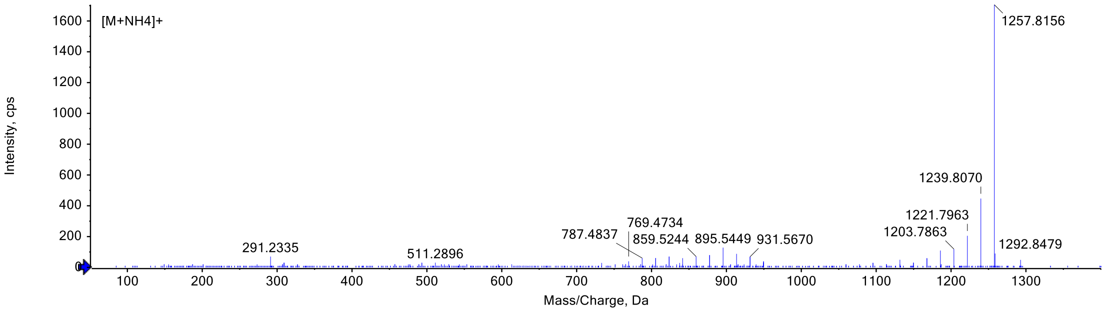

Spectrum from 20231024S4_A80.wiff2 (sample 1) - S4_A_80%, Experiment 9, +IDA TOF MSMS (50 - 2000) from 9.980 min Precursor: 1257.8 Da, +1, CE: 35.0, CES: 15.0
With 5 other merged spectra

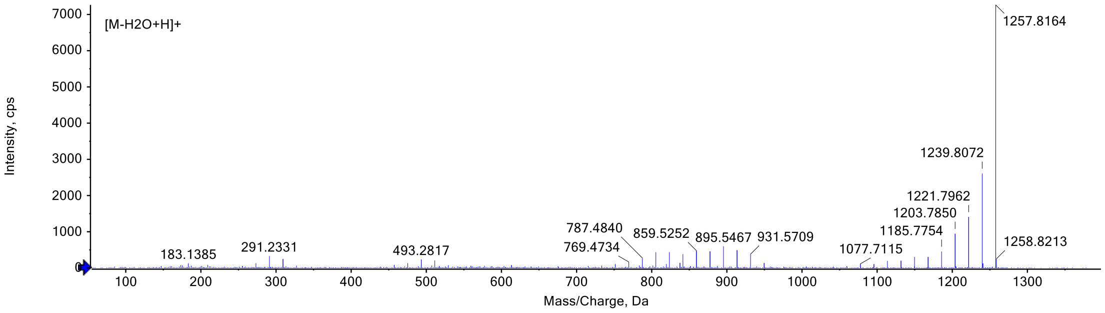

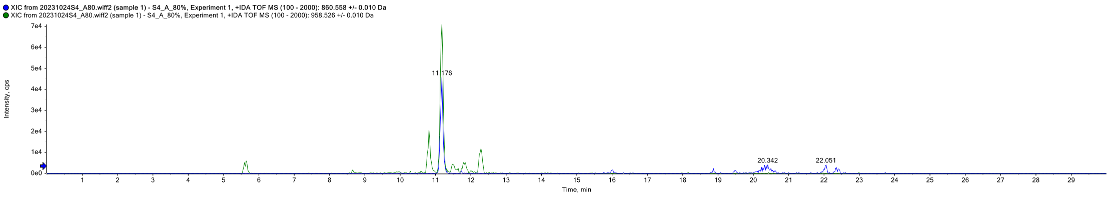

Spectrum from 20231024S4_A80.wiff2 (sample 1) - S4_A_80%, Experiment 11, +IDA TOF MSMS (50 - 2000) from 11.177 min Precursor: 958.5 Da, +1, CE: 35.0, CES: 15.0

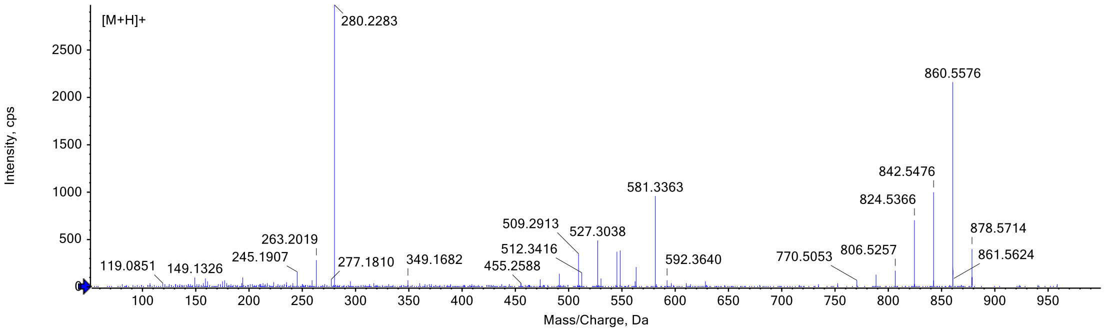

Spectrum from 20231024S4_A80.wiff2 (sample 1) - S4_A_80%, Experiment 11, +IDA TOF MSMS (50 - 2000) from 11.200 min Precursor: 860.6 Da, +1, CE: 35.0, CES: 15.0

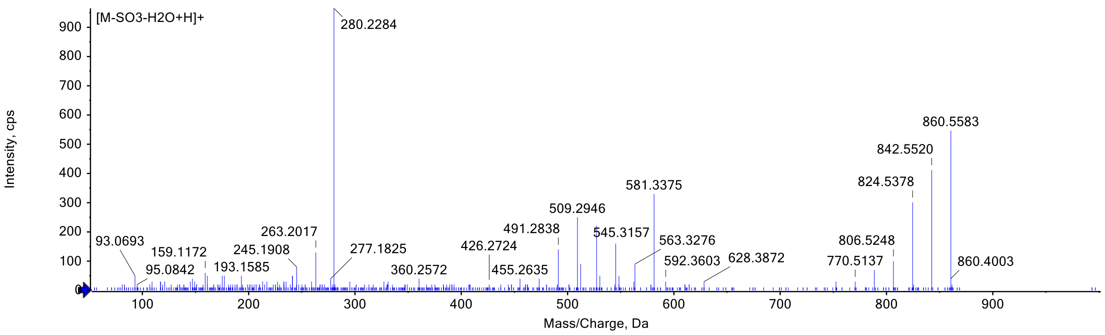

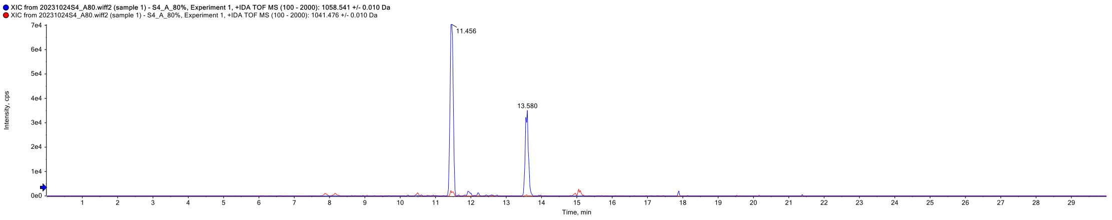

Spectrum from 20231024S4_A80.wiff2 (sample 1) - S4_A_80%, Experiment 10, +IDA TOF MSMS (50 - 2000) from 11.474 min Precursor: 1058.5 Da, +1, CE: 35.0, CES: 15.0
With 4 other merged spectra

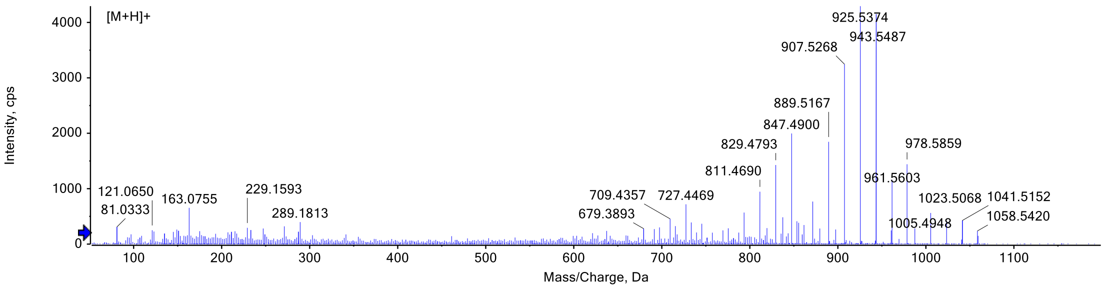

Spectrum from 20231024S4_A80.wiff2 (sample 1) - S4_A_80%, Experiment 9, +IDA TOF MSMS (50 - 2000) from 11.449 min Precursor: 1041.5 Da, +1, CE: 35.0, CES: 15.0
With 1 other merged spectrum

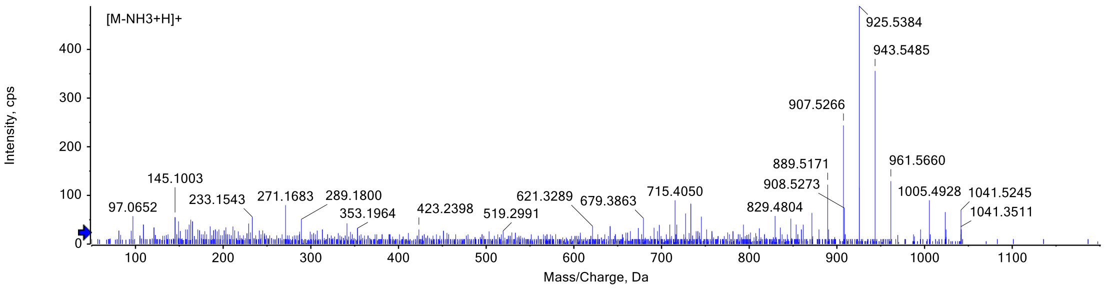

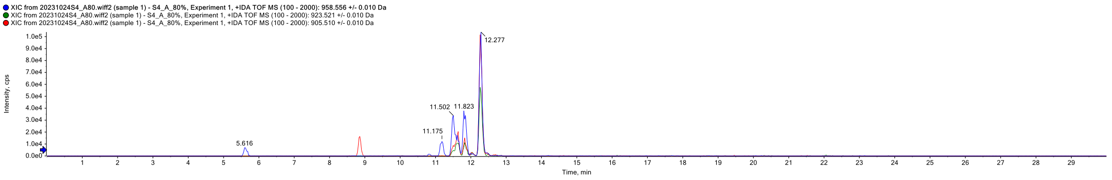

Spectrum from 20231024S4_A80.wiff2 (sample 1) - S4_A_80%, Experiment 9, +IDA TOF MSMS (50 - 2000) from 11.840 min Precursor: 958.6 Da, +1, CE: 35.0, CES: 15.0
With 1 other merged spectrum

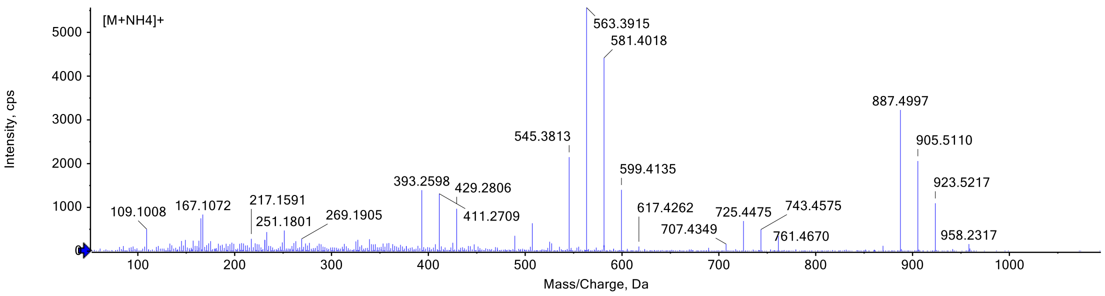

Spectrum from 20231024S4_A80.wiff2 (sample 1) - S4_A_80%, Experiment 9, +IDA TOF MSMS (50 - 2000) from 12.279 min Precursor: 923.5 Da, +1, CE: 35.0, CES: 15.0
With 1 other merged spectrum

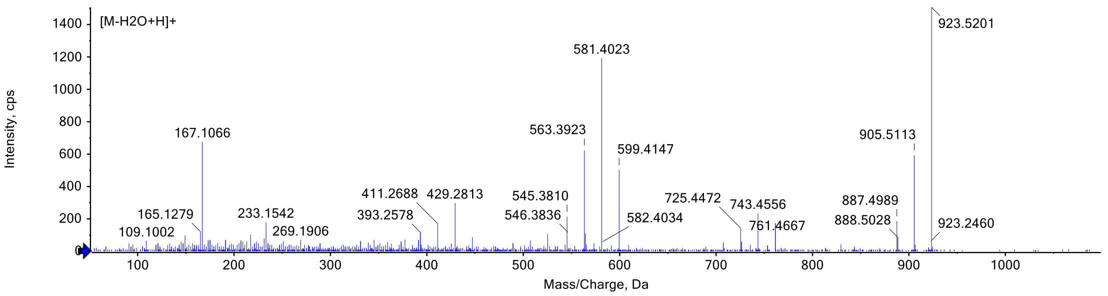

Spectrum from 20231024S4_A80.wiff2 (sample 1) - S4_A_80%, Experiment 8, +IDA TOF MSMS (50 - 2000) from 11.838 min Precursor: 905.5 Da, +1, CE: 35.0, CES: 15.0
With 1 other merged spectrum

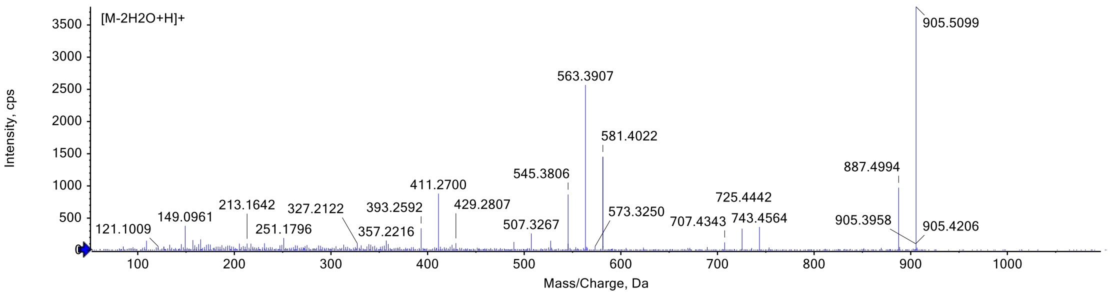

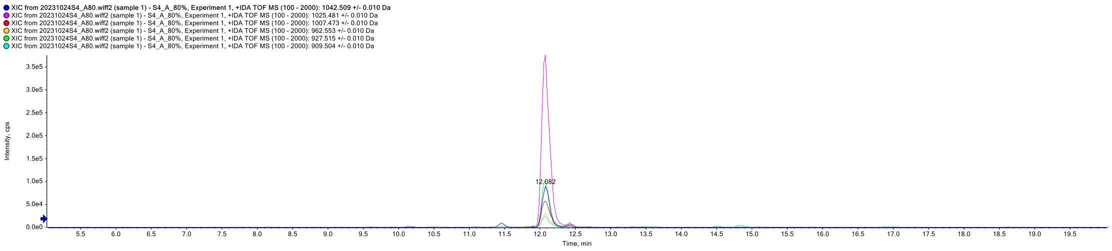

Spectrum from 20231024S4_A80.wiff2 (sample 1) - S4_A_80%, Experiment 10, +IDA TOF MSMS (50 - 2000) from 12.165 min Precursor: 1042.5 Da, +2, CE: 35.0, CES: 15.0
With 6 other merged spectra

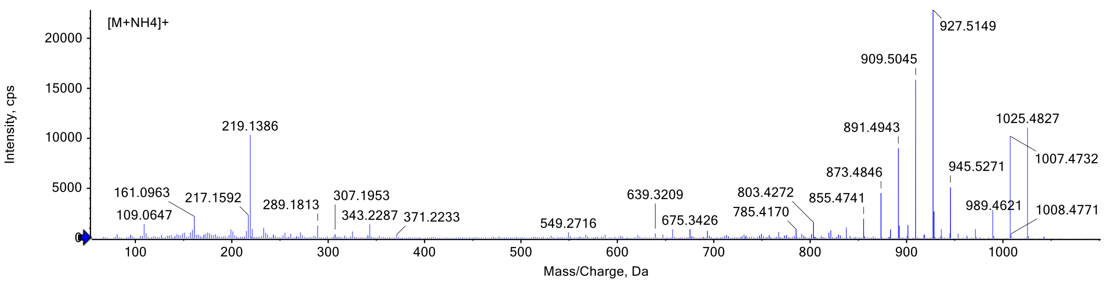

Spectrum from 20231024S4_A80.wiff2 (sample 1) - S4_A_80%, Experiment 9, +IDA TOF MSMS (50 - 2000) from 12.070 min Precursor: 1025.5 Da, +1, CE: 35.0, CES: 15.0
With 8 other merged spectra

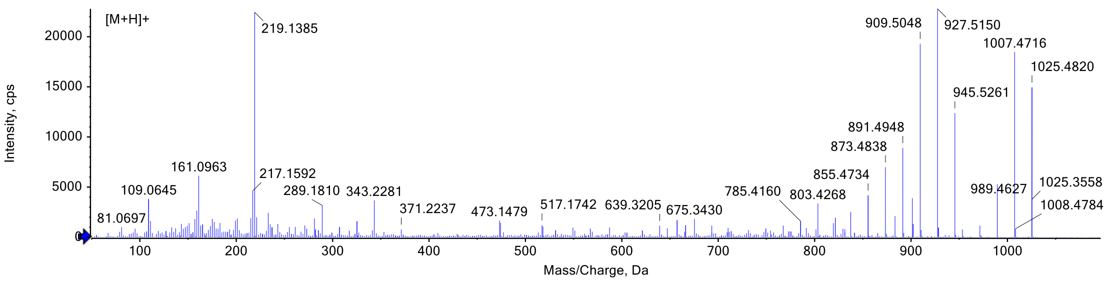

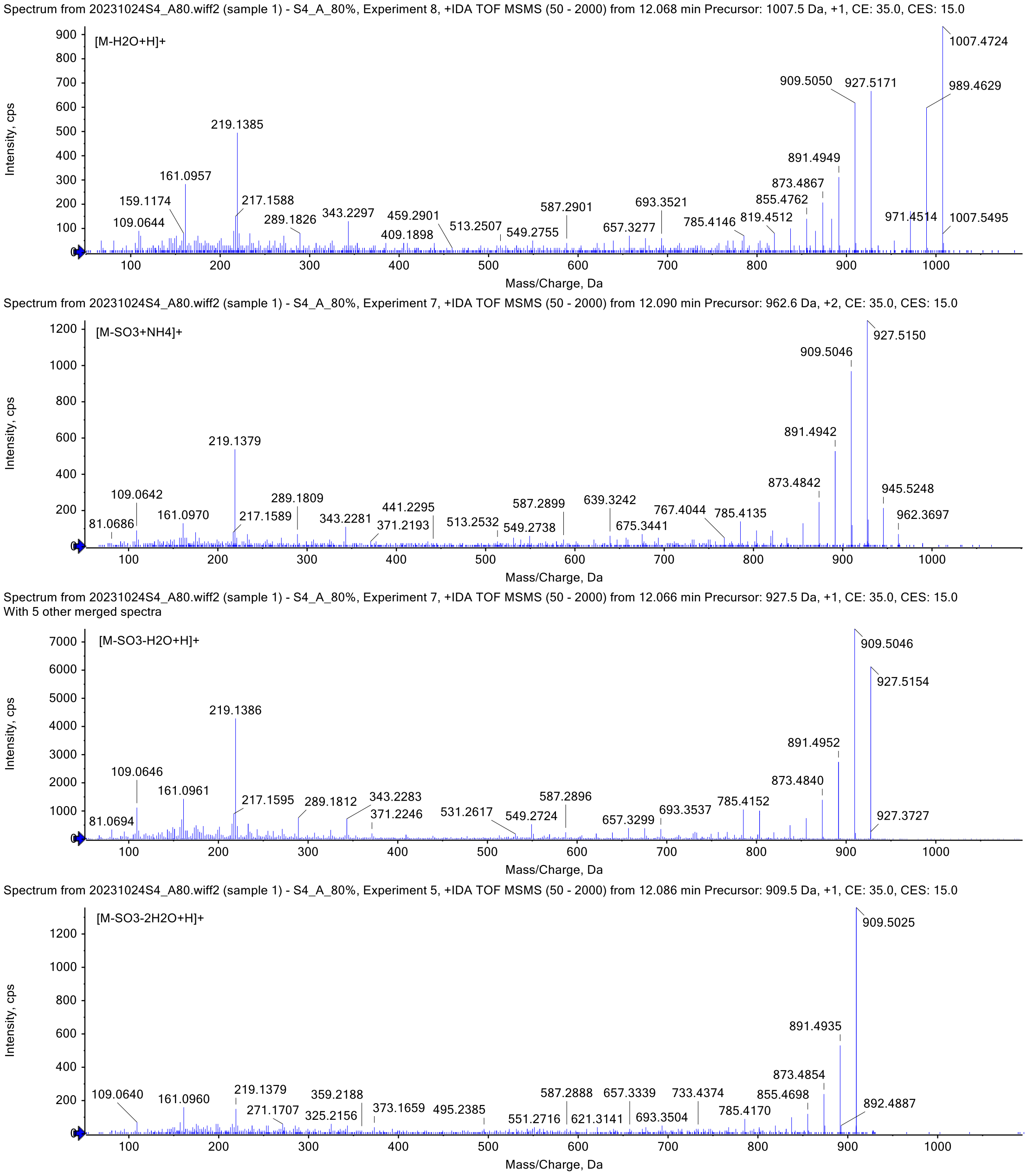

Figure S7. The MS^1 and MS^2 spectra of aligned adducts in *Gambierdiscus caribaeus* S4.

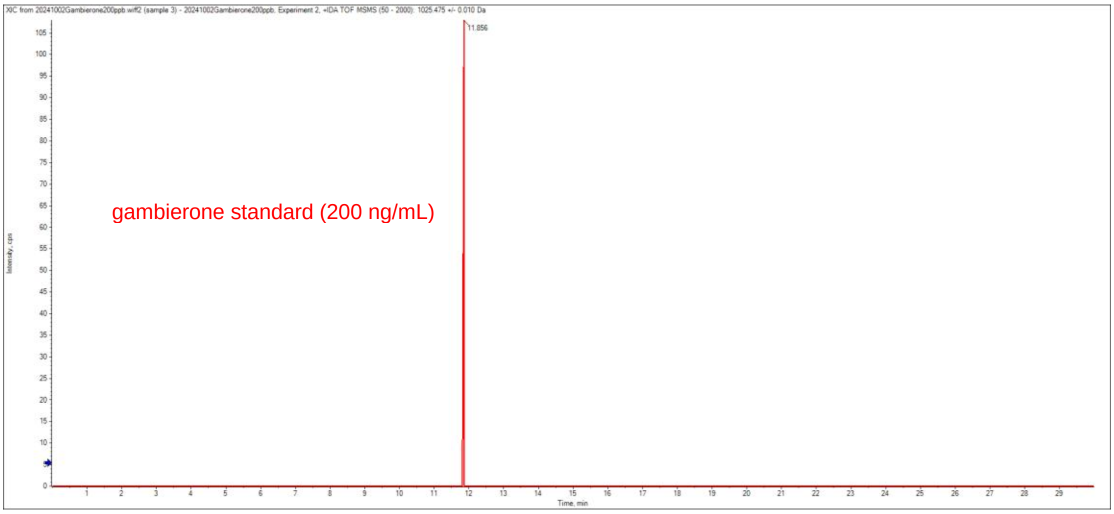
gambierone standard (200 ng/mL)
11.856
Time, min

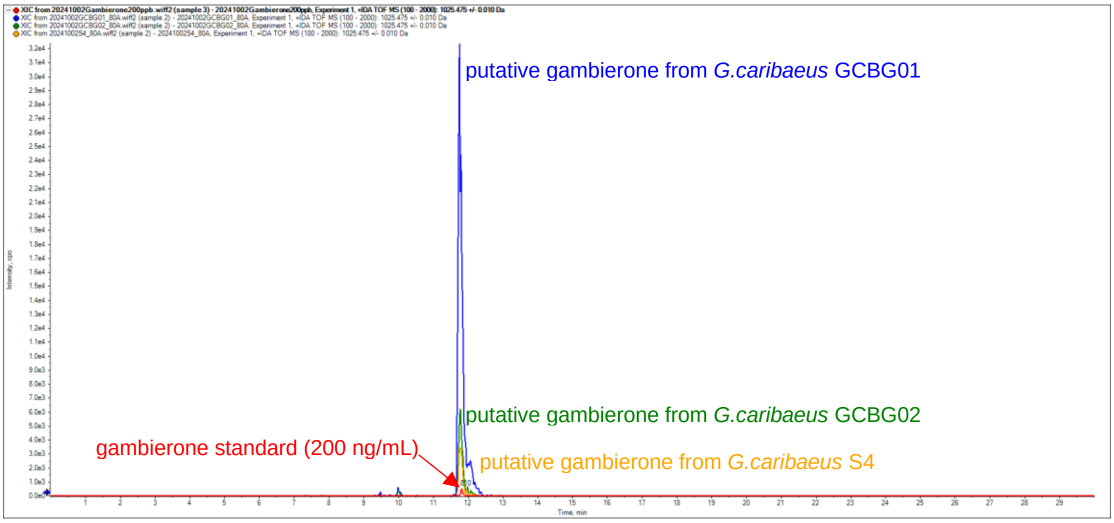
putative gambierone from G.caribaeus GCBG01
putative gambierone from G.caribaeus GCBG02
gambierone standard (200 ng/mL)
putative gambierone from G.caribaeus S4
Time, min

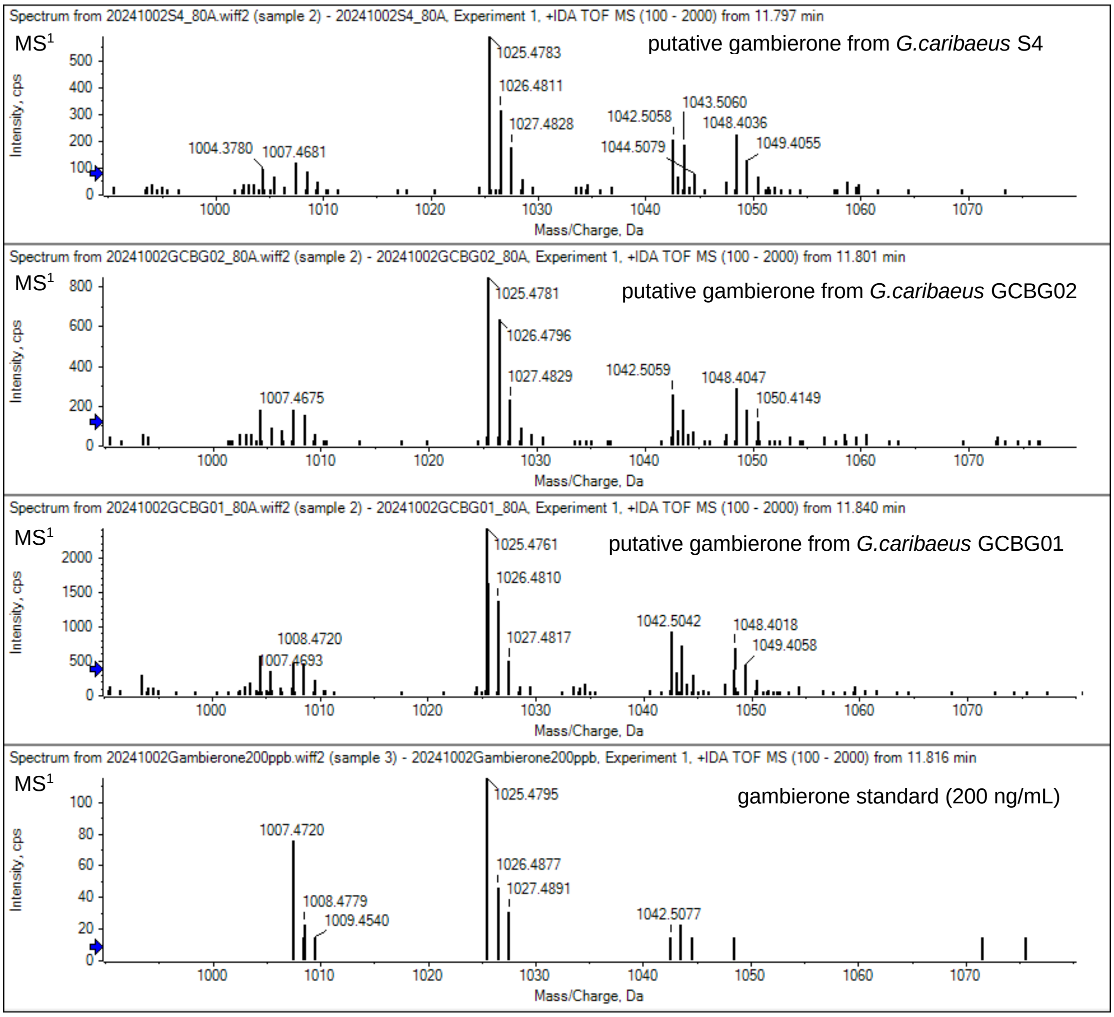

Figure S8. Extraction ion chromatograms (XICs) and MS^1 spectra of gambierone standard and putative gambierone from *G.caribaeus* GCBG01, *G.caribaeus* GCBG02, and *G.caribaeus* S4.

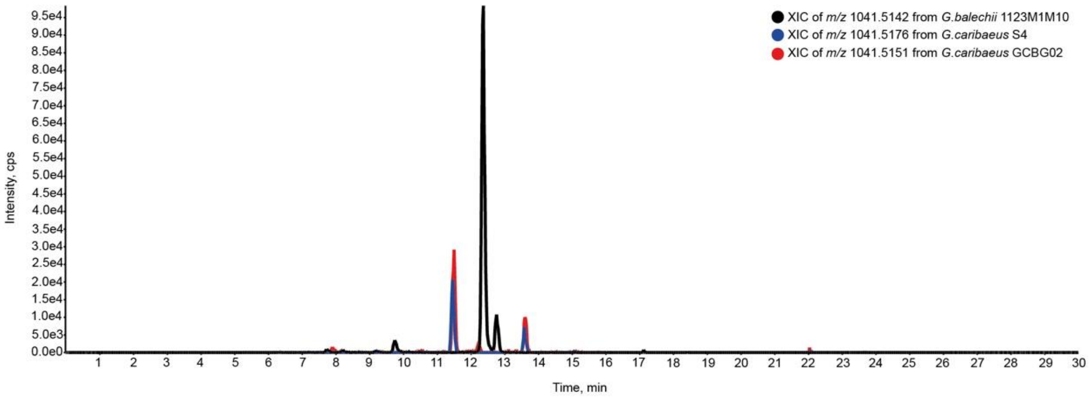

Figure S9. Extraction ion chromatograms (XICs) of ions at *m/z* 1041 from *G.balechii* 1123M1M10, *G.caribaeus* S4, and *G.caribaeus* GCBG02.

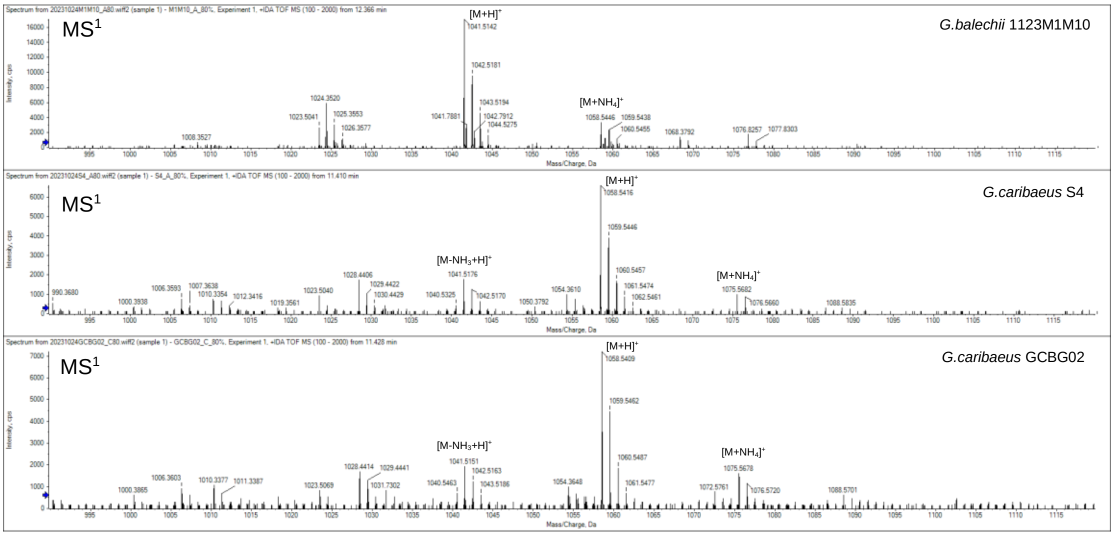

Figure S10. MS^1 spectra of 12,13-dihydro-44-methylgambierone from *G.balechii* 1123M1M10, and annotated 12,13-dihydro-44-methylgambierone from *G.caribaeus* S4 and *G.caribaeus* GCBG02.

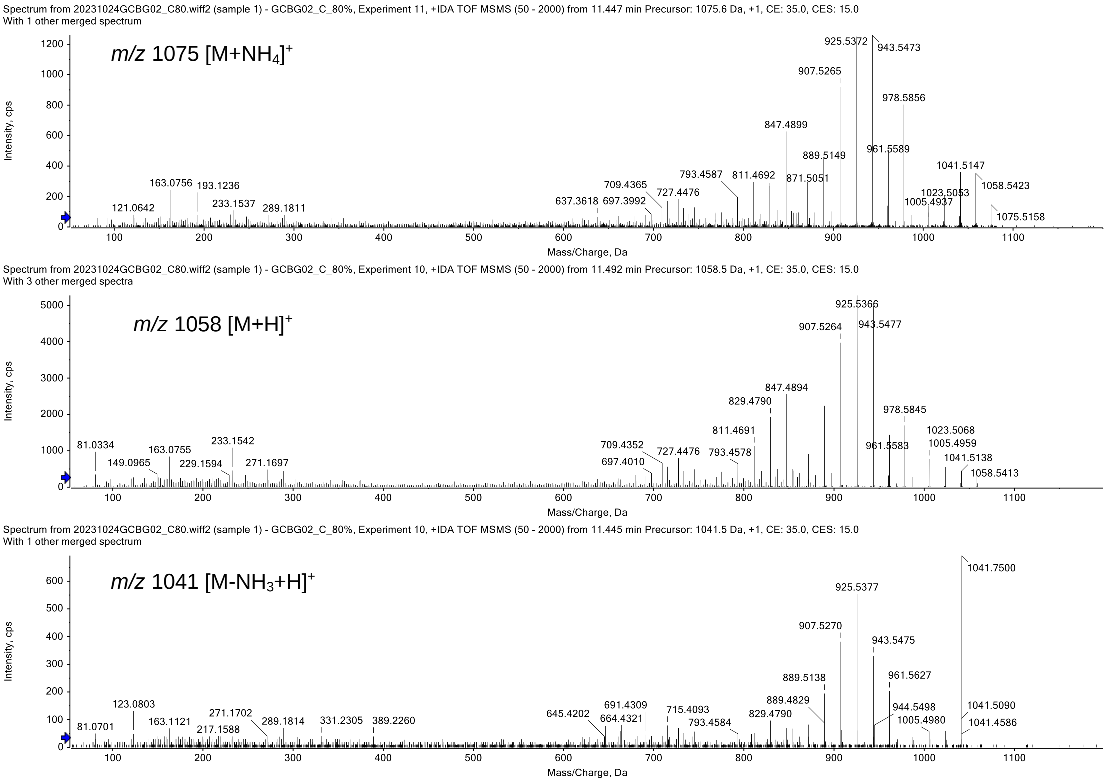

Figure S11. MS^2 spectra of ions at *m/z* 1075, 1058, and 1041 from *G.caribaeus* GCBG02.

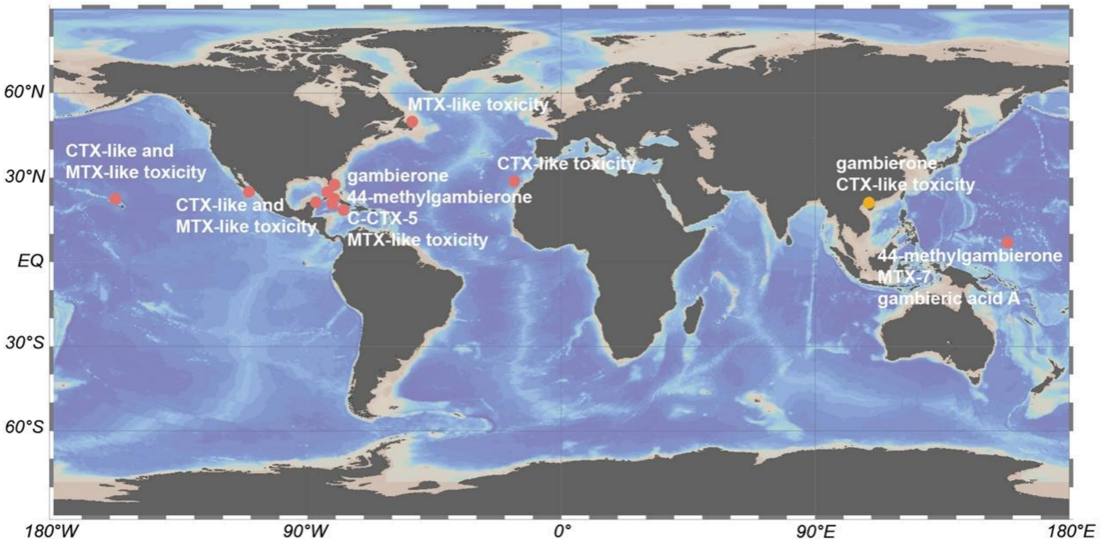

Figure S12. Distribution, toxicity assessment, and detection of polyether toxins in *Gambierdiscus caribaeus*. Species reported in the references are marked in red, while those described in this study are highlighted in yellow.

User manual and code of the Toxin-Screening program

Step 1: HPLC-HRMS/MS data was processed by MSDIAL 4.9 to extract the features to form the .mgf files. The parameters of MSDIAL 4.9 were listed in Table S10.

Step 2: Use software development environments, such as VSCode and PyCharm, to open the .mgf file and replace all occurrences of the keywords 'FEATURE_ID' with 'TITLE'.

Step 3: Put the modified .mgf file, toxin_database.xlsx, and Toxin-Screen code file into a folder. Then run the following code in VSCode or PyCharm.

```
import csv
import pandas as pd
from pyteomics import mgf
from matplotlib import pyplot as plt
import pdb


file_path = 'toxin_database.xlsx'
df = pd.read_excel(file_path, header=None)
toxins_list = df.values.tolist()


mgf_new_1 = []
```

```
mgf_new_2 = []
with mgf.read('xiaowan_test.mgf') as mgf_input:
    # Processing mgf_input
    for i, mgf_ele in enumerate(mgf_input):
        score_nH2O = 0
        score_SO3_nH2O = 0
        score_2SO3_nH2O = 0
        score_NH3_nH2O = 0
        score_NH3_SO3_nH2O = 0
        score_NH3_2SO3_nH2O = 0
        score_Na_nH2O = 0
        score_Na_SO3_nH2O = 0
        score_Na_2SO3_nH2O = 0
        mgf_array = mgf_ele['m/z array']

        # ions at m/z >= 700 will be analyzed
        if float(mgf_ele['params']['title']) < 700:
            continue

        # 1. First condition
        # 1.1 protonated adduct
        temp_mz = 0
        temp_it = 0
        for j in range(len(mgf_array)):
            if abs(mgf_array[j] - float(mgf_ele['params']['title'])) <= 1e-2 \
                and mgf_ele['intensity array'][j] > temp_it:
                temp_mz = mgf_array[j]
                temp_it = mgf_ele['intensity array'][j]
        for temp_value in [18.0106, 79.9568, 97.9674]:
            if temp_mz == 0:
                for j in range(len(mgf_array)):
                    if abs(float(mgf_ele['params']['title']) - mgf_array[j] - temp_value) <= 1e-
2 \
```

```
                and mgf_ele['intensity array'][j] > temp_it:
                temp_mz = mgf_array[j]
                temp_it = mgf_ele['intensity array'][j]

    # fragments analysis
    if temp_mz != 0:
        for j in range(len(mgf_array)):
            for temp_value in [18.0106, 36.0211, 54.0317, 72.0423, 90.0528, 108.0634,
126.0740, 144.0845, 162.0951, 180.1056]:
                if abs(float(mgf_ele['params']['title']) - mgf_array[j] - temp_value) <= 1e-
2:
                    score_nH2O += 1
            for temp_value in [79.9568, 97.9674, 115.9779, 133.9885, 151.9991,
170.0096]:
                if abs(float(mgf_ele['params']['title']) - mgf_array[j] - temp_value) <= 1e-
2:
                    score_SO3_nH2O += 1
            for temp_value in [159.9136, 177.9242, 195.9348, 213.9453, 231.9559,
249.9665]:
                if abs(float(mgf_ele['params']['title']) - mgf_array[j] - temp_value) <= 1e-
2:
                    score_2SO3_nH2O += 1

    # 1.2 ammonium adduct
    if score_nH2O == score_SO3_nH2O == score_2SO3_nH2O == 0:
        temp_mz = 0
        temp_it = 0
        for j in range(len(mgf_array)):
            if abs(mgf_array[j] - float(mgf_ele['params']['title'])) <= 1e-2 \
                and mgf_ele['intensity array'][j] > temp_it:
                temp_mz = mgf_array[j]
                temp_it = mgf_ele['intensity array'][j]
        for temp_value in [18.0106, 79.9568, 97.9674]:
            if temp_mz == 0:
```

```
                    for j in range(len(mgf_array)):
                        if abs(float(mgf_ele['params']['title']) - mgf_array[j] - temp_value) <=
1e-2 \
                            and mgf_ele['intensity array'][j] > temp_it:
                            temp_mz = mgf_array[j]
                            temp_it = mgf_ele['intensity array'][j]
            # fragments analysis
            if temp_mz != 0:
                for j in range(len(mgf_array)):
                    for temp_value in [35.0371, 53.0476, 71.0582, 89.0688, 107.0793,
125.0899, 143.1005, 161.1110, 179.1216, 197.1321]:
                        if abs(float(mgf_ele['params']['title']) - mgf_array[j] - temp_value) <=
1e-2:
                            score_NH3_nH2O += 1
                    for temp_value in [96.9834, 114.9939, 133.0044, 151.0150, 169.0256,
187.0361]:
                        if abs(float(mgf_ele['params']['title']) - mgf_array[j] - temp_value) <=
1e-2:
                            score_NH3_SO3_nH2O += 1
                    for temp_value in [176.9401, 194.9507, 212.9613, 230.9718, 248.9824,
266.9930]:
                        if abs(float(mgf_ele['params']['title']) - mgf_array[j] - temp_value) <=
1e-2:
                            score_NH3_2SO3_nH2O += 1


                # 1.3 sodium adduct
                # fragments analysis
                if score_nH2O == score_SO3_nH2O == score_2SO3_nH2O == 0 and
score_NH3_nH2O == score_NH3_SO3_nH2O == score_NH3_2SO3_nH2O == 0:
                    for j in range(len(mgf_array)):
                        for temp_value in [39.9926, 58.0031, 76.0137, 94.0243, 112.0348,
130.0454, 148.0560, 166.0665, 184.0771, 202.0876]:
                            if abs(float(mgf_ele['params']['title']) - mgf_array[j] - temp_value)
<= 1e-2:
                                score_Na_nH2O += 1
```

```
                        for temp_value in [101.9388, 119.9494, 137.9599, 155.9705,
173.9811, 191.9916]:
                            if abs(float(mgf_ele['params']['title']) - mgf_array[j] - temp_value)
<= 1e-2:
                                score_Na_SO3_nH2O += 1
                        for temp_value in [181.8956, 199.9062, 217.9168, 235.9273,
253.9379, 271.9485]:
                            if abs(float(mgf_ele['params']['title']) - mgf_array[j] - temp_value)
<= 1e-2:
                                score_Na_2SO3_nH2O += 1


        mgf_new_1.append([float(mgf_ele['params']['title']),
float(format(float(float(mgf_ele['params']['title'])), '.4f')),
            float(format((float(mgf_ele['params']['rtinminutes'])), '.3f')),
            mgf_ele['params']['charge'], mgf_array, mgf_ele['intensity array'],
score_nH2O, score_SO3_nH2O, score_2SO3_nH2O, \
                score_NH3_nH2O, score_NH3_SO3_nH2O, score_NH3_2SO3_nH2O,
score_Na_nH2O, score_Na_SO3_nH2O, score_Na_2SO3_nH2O])


for mgf_ele in mgf_new_1:
    mgf_array = mgf_ele[4]
    intensity_array = mgf_ele[5]
    mgf_array_500 = [value for value in mgf_array if value <= 500]
    intensity_500 = [intensity_array[k] for k in range(len(mgf_array)) if mgf_array[k]
<= 500]
    str_temp = ''
    score_nH2O, score_SO3_nH2O, score_2SO3_nH2O, score_NH3_nH2O,
score_NH3_SO3_nH2O, score_NH3_2SO3_nH2O, score_Na_nH2O,
score_Na_SO3_nH2O, score_Na_2SO3_nH2O = mgf_ele[-9:]
    temp_mz = 0
    temp_it = 0
    ciguatoxins_temp_score = 0
    condition_23 = condition_24 = \
                condition_25 = condition_26 = condition_27 = condition_28 =
condition_29 = False
```

```
    if (score_nH2O == score_SO3_nH2O == score_2SO3_nH2O == 0 and
(score_NH3_nH2O !=0 or score_NH3_SO3_nH2O != 0 or
score_NH3_2SO3_nH2O != 0)) \
        or (score_nH2O == score_SO3_nH2O == score_2SO3_nH2O == 0 and
score_NH3_nH2O == score_NH3_SO3_nH2O == score_NH3_2SO3_nH2O == 0
and score_Na_nH2O == score_Na_SO3_nH2O == score_Na_2SO3_nH2O == 0):
        #1.4 ammonium adducts change to protonated adducts
        for temp_value in [17.0265, 35.0371, 96.9834, 114.9939]:
            if temp_mz == 0:
                for j in range(len(mgf_array)):
                    if abs(mgf_ele[1] - mgf_array[j] - temp_value) <= 1e-2 \
                        and mgf_ele[5][j] > temp_it:
                        temp_mz = mgf_array[j]
                        temp_it = mgf_ele[5][j]


        # fragments analysis
        if temp_mz != 0:
            for j in range(len(mgf_array)):
                for temp_value in [18.0106, 36.0211, 54.0317, 72.0423, 90.0528, 108.0634,
126.0740, 144.0845, 162.0951, 180.1056]:
                    if abs(mgf_ele[1] - mgf_array[j] - temp_value) <= 1e-2:
                        score_NH3_nH2O += 1
                for temp_value in [79.9568, 97.9674, 115.9779, 133.9885, 151.9991,
170.0096]:
                    if abs(mgf_ele[1] - mgf_array[j] - temp_value) <= 1e-2:
                        score_NH3_SO3_nH2O += 1
                for temp_value in [159.9136, 177.9242, 195.9348, 213.9453, 231.9559,
249.9665]:
                    if abs(mgf_ele[1] - mgf_array[j] - temp_value) <= 1e-2:
                        score_NH3_2SO3_nH2O += 1


    if (score_nH2O == score_SO3_nH2O == score_2SO3_nH2O == 0 and
score_NH3_nH2O == score_NH3_SO3_nH2O == score_NH3_2SO3_nH2O == 0
and (score_Na_nH2O !=0 or score_Na_SO3_nH2O != 0 or score_Na_2SO3_nH2O !=
0)) \
```

```
            or (score_nH2O == score_SO3_nH2O == score_2SO3_nH2O == 0 and
score_NH3_nH2O == score_NH3_SO3_nH2O == score_NH3_2SO3_nH2O == 0
and score_Na_nH2O == score_Na_SO3_nH2O == score_Na_2SO3_nH2O == 0):
        #1.5 sodium adducts change to protonated adducts
        for temp_value in [21.9820, 39.9925, 101.9388, 119.9493]:
            if temp_mz == 0:
                for j in range(len(mgf_array)):
                    if abs(mgf_ele[1] - mgf_array[j] - temp_value) <= 1e-2 \
                        and mgf_ele[5][j] > temp_it:
                        temp_mz = mgf_array[j]
                        temp_it = mgf_ele[5][j]
        # fragments analysis
        if temp_mz != 0:
            for j in range(len(mgf_array)):
                for temp_value in [18.0106, 36.0211, 54.0317, 72.0423, 90.0528, 108.0634,
126.0740, 144.0845, 162.0951, 180.1056]:
                    if abs(mgf_ele[1] - mgf_array[j] - 18.0106) <= 1e-2:
                        score_Na_nH2O += 1
                for temp_value in [79.9568, 97.9674, 115.9779, 133.9885, 151.9991,
170.0096]:
                    if abs(mgf_ele[1] - mgf_array[j] - 79.9568) <= 1e-2:
                        score_Na_SO3_nH2O += 1
                for temp_value in [159.9136, 177.9242, 195.9348, 213.9453, 231.9559,
249.9665]:
                    if abs(mgf_ele[1] - mgf_array[j] - 159.9136) <= 1e-2:
                        score_Na_2SO3_nH2O += 1


    # 2. Second condition
    score_sum = sum(mgf_ele[-9:])
    if score_sum >= 3:
        if score_sum >= 1:
            for j in range(len(mgf_array)):
                if mgf_array[j] <= 161.0977 and mgf_array[j] >= 161.0945:
                    condition_23 = True
```

```
            if mgf_array[j] <= 219.1414 and mgf_array[j] >= 219.1358 and condition_23 == True and intensity_array[j] == max(intensity_500):
                str_temp = 'gambierones'
                condition_24 = True

            if mgf_array[j] <= 215.1452 and mgf_array[j] >= 215.1408:
                condition_25 = True
            if mgf_array[j] <= 233.1559 and mgf_array[j] >= 233.1513 and condition_25 == True and intensity_array[j] == max(intensity_500):
                condition_26= True
                str_temp = 'gambierones'

            if mgf_array[j] <= 125.0974 and mgf_array[j] >= 125.0948:
                ciguatoxins_temp_score = 1
                if intensity_array[j] == max(intensity_500):
                    ciguatoxins_temp_score = ciguatoxins_temp_score + 1
            if mgf_array[j] <= 155.1083 and mgf_array[j] >= 155.1051:
                ciguatoxins_temp_score = ciguatoxins_temp_score + 1
                if intensity_array[j] == max(intensity_500):
                    ciguatoxins_temp_score = ciguatoxins_temp_score + 1
            if mgf_array[j] <= 447.2786 and mgf_array[j] >= 447.2696 and ciguatoxins_temp_score >= 3:
                condition_27= True
                str_temp = 'ciguatoxins'

            if mgf_array[j] <= 171.1033 and mgf_array[j] >= 171.0999:
                condition_28 = True
            if mgf_array[j] <= 445.2630 and mgf_array[j] >= 445.2540:
                condition_29 = True
            if mgf_array[j] <= 463.2736 and mgf_array[j] >= 463.2644 and condition_28 == True and condition_29 == True:
                str_temp = 'ciguatoxins'
    else:
        str_temp = ''
```

```
        mgf_ele_2 = mgf_ele[1:4] + mgf_ele[6:]
        mgf_ele_2.extend([score_sum, str_temp])

        # 3. known toxin annotation
        # mgf_ele_2 -> list[0:pemss, 1:rtinminutes, 2:charge, 3:score_1, 4:score_2, 5:score_3, 6:score_4, 7:score_5, 8:score_6, 9:score_4, 10:score_5, 11:score_6, 12:score_sum, 13:str, 14:library_hit_name, 15:library_hit_formula, 16:library_hit_adduct]
        if mgf_ele_2[12] >= 1:
            mgf_new_2.append(mgf_ele_2)
            for j in range(len(toxins_list)):
                if abs(mgf_new_2[-1][0] - toxins_list[j][2]) / toxins_list[j][2] <= 1 * 1e-5:
                    mgf_new_2[-1].extend([toxins_list[j][0], toxins_list[j][1], toxins_list[j][2], toxins_list[j][3]])

with open('xiaowan_test.csv', 'w', newline='') as comb_00:
    writer = csv.writer(comb_00)
    writer.writerow(['m/z', 'RT', 'charge', 'score_nH2O', 'score_SO3_nH2O', 'score_2SO3_nH2O', 'score_NH3_nH2O', 'score_NH3_SO3_nH2O', 'score_NH3_2SO3_nH2O', 'score_Na_nH2O', 'score_Na_SO3_nH2O', 'score_Na_2SO3_nH2O', 'total_score', 'type', 'library_hit_name', 'library_hit_formula', 'library_hit_adduct', 'library_precusor_type'])
    for comb_row in mgf_new_2:
        writer.writerow(comb_row)

plt.figure()
for comb_row in mgf_new_2:
    plt.scatter(comb_row[1], comb_row[0], c='green', alpha=0.5)
    plt.scatter(comb_row[1], comb_row[0], marker='D', c='red', alpha=0.5)
    if len(comb_row) == 17:
        plt.scatter(comb_row[1], comb_row[0], s=6**2, marker='.', c='black', alpha=0.9)
        plt.annotate(text=comb_row[14], xy=(comb_row[1], comb_row[0]), xytext=(comb_row[1], comb_row[0]+100), color='b', fontsize=6, arrowprops=dict(facecolor='black', arrowstyle='->'))
```

```
y_start, y_end = plt.ylim()
plt.ylim(700, y_end)
plt.title('xiaowan_test')
plt.xlabel('RT')
plt.ylabel('M/Z')
plt.show()
```
